# Programmable multispecific DNA-origami-based T-cell engagers

**DOI:** 10.1038/s41565-023-01471-7

**Published:** 2023-08-17

**Authors:** Klaus F. Wagenbauer, Nhi Pham, Adrian Gottschlich, Benjamin Kick, Viktorija Kozina, Christopher Frank, Daniela Trninic, Pierre Stömmer, Ruth Grünmeier, Emanuele Carlini, Christina Angeliki Tsiverioti, Sebastian Kobold, Jonas J. Funke, Hendrik Dietz

**Affiliations:** 1 Department of Biosciences, School of Natural Sciences, Technical University of Munich, Garching, Germany; 2 Munich Institute of Biomedical Engineering, Technical University of Munich, Garching, Germany; 3 Division of Clinical Pharmacology, University Hospital, LMU Munich, Member of the German Center for Lung Research (DZL), Munich, Germany; 4 Department of Medicine III, University Hospital, LMU Munich, Munich, Germany; 5 German Cancer Consortium (DKTK), Partner Site Munich, Munich, Germany; 6 Einheit für Klinische Pharmakologie (EKLiP), Helmholtz Munich, Research Center for Environmental Health (HMGU), Neuherberg, Germany

**Keywords:** Nanostructures, Drug delivery, DNA nanomachines, Nanoparticles

## Abstract

Multispecific antibodies have emerged as versatile therapeutic agents, and therefore, approaches to optimize and streamline their design and assembly are needed. Here we report on the modular and programmable assembly of IgG antibodies, F(ab) and scFv fragments on DNA origami nanocarriers. We screened 105 distinct quadruplet antibody variants in vitro for the ability to activate T cells in the presence of target cells. T-cell engagers were identified, which in vitro showed the specific and efficient T-cell-mediated lysis of five distinct target cell lines. We used these T-cell engagers to target and lyse tumour cells in vivo in a xenograft mouse tumour model. Our approach enables the rapid generation, screening and testing of bi- and multispecific antibodies to facilitate preclinical pharmaceutical development from in vitro discovery to in vivo proof of concept.

## Abstract (web summary)

A synthetic nanocarrier based on DNA origami chassis offers control over valency, orientation and spatial arrangement of antibodies for simultaneously engaging immune signalling pathways, checkpoint inhibition and targeted co-stimulation in anticancer immunotherapy in vivo.

## Main

Programmable self-assembly with DNA origami enables fabricating discrete nanoscale objects with structurally well-defined two-dimensional and three-dimensional shapes from DNA molecules^1–5^, including nanoscale devices^6–8^, functional materials^9,10^ and higher-order objects^11,12^. DNA origami objects are addressable and can be modified with various biomolecules in a site-specific fashion^13,14^. Previous studies have demonstrated the attachment of antibodies to DNA origami objects^15,16^, the binding of antibody-conjugated DNA origami objects to cell surfaces^14,17,18^ and the modulation of T-cell function^19,20^. Recent developments such as the cost-efficient mass production of DNA origami raw materials and stabilization approaches for in vivo application^21–23^ may enable the clinical translation of diverse therapeutical concepts such as DNA origami biomedical nanorobots^10,24^.

In parallel to the advances in DNA nanotechnology, cancer immunotherapies have contributed to a paradigm shift in oncological treatment landscapes^25^. In particular, T-cell-centred immunotherapies (for example, immune-checkpoint-inhibiting antibodies) are now established in clinical practice in various cancer entities^26^. In addition, in B-cell-derived haematological malignancies (such as acute lymphoblastic leukaemia (ALL) or B-cell lymphomas), T-cell-engaging antibodies have led to clinical responses even in otherwise treatment-refractory patients^27^. The Federal Drug Agency (FDA) and European Medicine Agency (EMA) have granted approval for blinatumomab, a CD19-CD3-bispecific T-cell engager (BiTe), prolonging the overall survival of patients.

In B-cell malignancies, using B-cell-associated antigens such as CD19 and CD20 has proven feasible, efficacious and manageable from a safety perspective, partly owing to established clinical treatments to manage induced B-cell aplasia^28^. However, this is unique to B-cell-targeting agents and cannot be expected in other diseases. In solid cancers, tumour-associated antigens are often co-expressed on vital epithelial tissues, creating the risk for severe on-target off-tumour toxicities^29^. Increasing cell-type specificity, for example, by the simultaneous targeting of multiple-tumour-associated antigens has the potential to minimize the risk for severe on-target off-tumour toxicities^30^. In addition, targeting more than one antigen on a target cell may prove beneficial in preventing antigen-negative relapse, as sequential or simultaneous multiple targeting will enhance therapeutic pressure and counter the development of negative variants.

Thus, multispecific molecules targeting cancer vulnerabilities are needed to leverage immune cell potential in oncology. A large number of drug candidates are currently in preclinical and clinical development, with the focus shifting from bispecific antibodies and BiTe formats (four on the market and more than 100 in clinical development) towards formats with increased specificities or enhanced pharmacokinetic properties (eight candidates in clinical development)^31–33^. Various approaches have been developed to produce multispecific antibodies, most of which rely on fusing engineered antibody domains^34^. Although these approaches support controlling the degree of valency, the spatial geometric arrangements of the domains are restricted by the structural constraints of the protein scaffold used.

Here we used programmable self-assembly with DNA origami to create a synthetic antibody carrier platform called programmable T-cell engager (PTE). PTEs offer desirable properties for T-cell engagement, including the capability to modularly position antibodies (IgG, F(ab) or scFv) with control over valency, orientation and spatial arrangement. We provide the proof of concept of specific T-cell engaging in vitro and validate the PTE functionality in leukaemia models in vivo.

## Assembly and screening of IgG-based PTEs

**Fig. 1:**
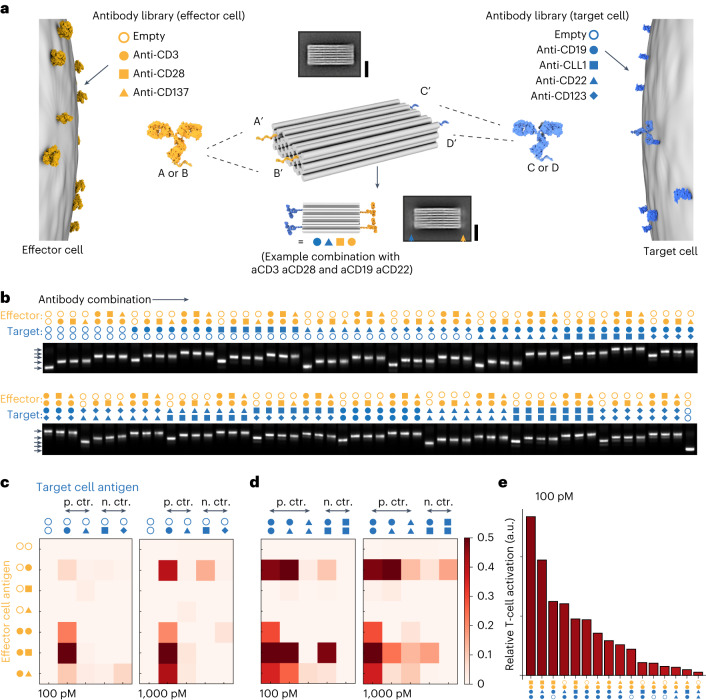
Production and functional screening of 105 unique antibody combinations on a DNA chassis. **a**, Schematic of a multispecific antibody chassis variant library created from a set of antibody–DNA conjugates. The symbols indicate the antibody, and the colour indicates the engaged cell type. Antibodies are covalently tagged with DNA handles with the sequences A, B, C or D, depending on the library, and the sequences are complementary to DNA handles on the chassis (centre). The chassis carries four DNA handles. Antibody chassis variants are produced by mixing the respective antibodies from the libraries with the DNA chassis. Variants are named by their antibody combination (the centre bottom shows an example combination). Two reference-free class averages calculated from single-particle TEM micrographs. Scale bar, 20 nm. The top average shows the platform without antibodies and the bottom average image shows the platform with four IgG antibodies, indicated by the orange and blue arrow heads pointing to the blurred additional signal in the average image. **b**, Montage of laser-scanned images of agarose gels on which 105 variants were electrophoresed that were incubated with different antibody combinations (as indicated by the symbols). The first and last lanes show a reference 5 MDa DNA origami object. **c**,**d**, T-cell activation was measured by using NFAT-luciferase Jurkat cell line in co-cultures with human ALL cell line NALM-6 in the presence of the indicated combinations (**c**,**d**). Relative T-cell activation (normalized to variants without target cell antibodies) of different variants for 100 pM and 1,000 pM DNA chassis concentrations. The icons in orange and blue indicate the respective antibodies used in the combination. **e**, Relative T-cell activation of the variants sorted for maximum activation according to the values in **c** and **d** at 100 pM. Source data

The ability to place IgG antibodies in a user-defined fashion on a DNA origami carrier is the prerequisite for building more complex multivalent configurations. We tested and optimized methods to meet these requirements in auxiliary experiments (Supplementary Notes 1 and 2 and Supplementary Figs. 1–3), including demonstrating multivalent cell binding and testing for cell internalization (Supplementary Note 3 and Supplementary Figs. 4 and 5). Building on these optimized methods, we created a tetravalent antibody carrier featuring four distinct antibody attachment sites (Fig. 1a). For each attachment site on the DNA origami chassis, we created a library of DNA-tagged antibodies carrying a sequence-complementary single-stranded DNA tag. To induce the activation of effector T cells, we chose anti-CD3, anti-CD28 and anti-CD137 antibodies (Fig. 1a, left). To mediate binding to the target cells, we chose antibodies against the known antigens CD19, CLL-1, CD22 and CD123. We prepared antibody–DNA conjugates and then assembled 105 unique antibody combinations. We validated the assembly of these PTEs via gel electrophoretic mobility analysis (Fig. 1b) and quantified the yield of fully assembled tetravalent combinations for each variant. The assembly yield varied between from 85% (variant 2× anti-CD123 2× anti-CD3) to 97% (variant 2× anti-CD19 2× anti-CD3).

To study PTE-induced T-cell activation, we used a nuclear factor of activated T cells (NFAT)-luciferase reporter assay^35^. We co-cultured CD19^+^, CD22^+^, CD123^−^ and CLL-1^–^ NALM-6 ALL cells with NFAT-luciferase-transduced Jurkat cells in the presence or absence of PTEs (Fig. 1c,d). Bivalent aCD3 IgG antibodies can crosslink T-cell receptors causing T-cell activation in the absence of target cells. We, therefore, subtracted the background signal generated by the PTEs that carried only T-cell antibodies (Supplementary Fig. 6). Here aCD19 × aCD3 constructs lead to the strongest T-cell activation, whereas aCD22 × aCD3 constructs induced weak T-cell activation (Fig. 1c). These results are in line with the differing densities of CD19 and CD22 molecules on NALM-6 cells^36^. Also, aCLL-1, aCD123 × aCD3 constructs did not cause target-cell-induced T-cell activation, proving the specificity of the PTEs. The inclusion of additional T-cell-activating antibodies (aCD28, aCD137) resulted in the increased activation of T cells compared with the single aCD19 × aCD3 variant, with the aCD19 × aCD3-aCD28 PTEs showing the strongest T-cell activation (Fig. 1c). Again, these results are compliant with the literature^37^.

Next, we analysed the dual-tumour-targeting constructs for their ability to activate T cells (Fig. 1d). We observed increased activation signals for two-target variants with a single activation of T cells via CD3 compared with single-target variants (Fig. 1d). The addition of a co-stimulatory domain to the dual-targeting variants further enhanced the signal. We observed the strongest activation for variants consisting of aCD3, aCD28 and two aCD19 antibodies or one aCD19 antibody and one aCD22 antibody (Fig. 1e). The aCLL-1 control constructs did not induce the activation of T cells.

## In vitro characterization of F(ab)-based PTEs

**Fig. 2:**
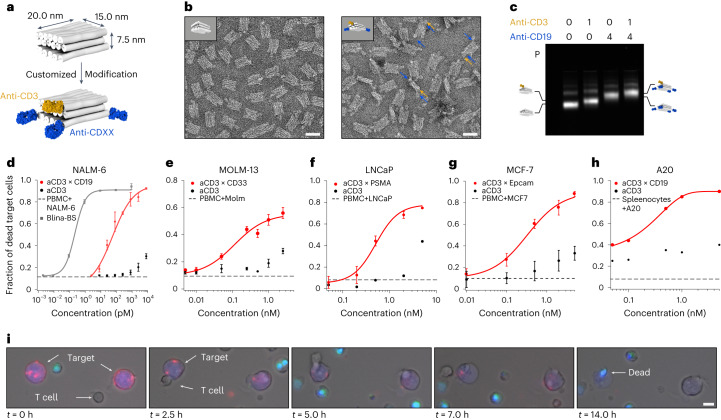
Programmable T-cell-mediated killing of target cells. **a**, Schematic of a multispecific brick-shaped antibody carrier (chassis) with dimensions of 10.0 × 15.0 × 7.5 nm^3^. The grey cylinders represent DNA double helices, and the F(ab) fragments are coloured in orange (anti-CD3) and blue (representing a F(ab) fragment for antigens located on the target cells). **b**, Negatively stained TEM image of the small chassis (left) and negatively stained TEM image of the small chassis with 1× anti-CD3 and 2× anti-CD19 F(ab) fragments (right). Scale bar, 25 nm. The arrows in blue and yellow highlight the attached F(ab) fragments as an example. **c**, Laser-scanned image of an agarose gel on which different samples were electrophoresed. The samples were prepared with different F(ab) fragment combinations (as indicated by the numbers). P, pocket; icons highlight the different antibody chassis variants; 0-0, reference for the migration of platform only. **d**–**h**, Cytotoxic T-cell-mediated target cell lysis assays. Fraction of dead target cells after 24 h as a function of PTE concentration in the assay (Supplementary Information). Effector (PBMC) and target cell ratio was chosen as 5:1. Red dots, multispecific T-cell-engaging variant with anti-CD3 and at least two target-specific F(ab) fragments for the respective cell line. Black dots, monospecific controls. Solid lines, Hill fit to the data. Dashed line, PBMC and target cells without PTE after 24 h. Grey squares, blinatumomab-biosimiliar (Blina-BS). The error bars to the data are standard deviations to the mean of three biological replicates. **i**, Live-cell fluorescence microscopy over 24 h of a mixture containing A20 cells (stained with CellTrace CSFE; blue) and splenocytes in a 1:5 ratio, and a variant (1× mu anti-CD3–4× mu anti-CD19) carrying a fluorescent tag (cyanine-5). A live–dead stain was used for the visualization of dead cells (SYTOX Orange; cyan). Scale bar, 2.5 µm. Source data

Protein-based bispecific antibodies can mediate the potent T-cell-directed lysis of tumour cells^38^. Our approach should allow us to assemble bispecific variants with similar capabilities. However, the size of the antibody and the arrangement of the paratopes are essential factors that can influence the efficacy of target cell killing^39^ (Supplementary Fig. 7). In this context, full-sized IgGs present limitations, such as Fc-domain-mediated binding to immune cells, which can cause undesired cell interactions^40^, and crosslinking receptors through their two paratopes, leading to non-specific target cell lysis (Supplementary Fig. 8). With these aspects in mind, we designed a smaller antibody carrier chassis (20.0 × 15.0 × 7.5 nm^3^) that can display an anti-CD3 F(ab) fragment for T-cell binding and up to four F(ab) fragments to recognize the target cell antigens (Fig. 2a,b and Supplementary Fig. 9). We placed the F(ab) fragments at the corners of the DNA origami chassis to realize a paratope-to-paratope distance of approximately 7.5 nm, which is comparable with reported cell–cell distances in immunological synapses^41^. The antibody attachment concepts established with the larger chassis were directly transferable to the small chassis and enabled fabricating multispecific variants with high yields (>98%), as seen by agarose gel electrophoresis and transmission electron microscopy (TEM) (Fig. 2b,c and Supplementary Fig. 10). To orient the F(ab) fragments on the DNA origami chassis, we site-specifically coupled the adapter DNA strands to one of the thiol groups of its disulfide bond, located opposite the paratope (Supplementary Fig. 11).

Using the smaller chassis, we prepared five bispecific PTE variants targeting four known human-tumour-associated antigens, namely, huCD19 (PTE-2×19-3), huCD33 (PTE-2×33-3), huEpCAM (PTE-2×EpCAM-3) and huPSMA (PTE-2×PSMA-3) or murine CD19 (mPTE-2×mCD19-3) (Fig. 2d–h). First, we tested if PTE-2×19-3 constructs induced the lysis of CD19^+^ NALM-6 cells in co-cultures with peripheral blood mononuclear cells (PBMC). Using flow cytometry, we observed the concentration-dependent lysis of the NALM-6 target cells after 24 h of co-culture. The lysis efficiency was higher than 90% at concentrations above 500 pM with a calculated potency of approximately 100 pM (EC_50_ value; Supplementary Fig. 12). In contrast, the monospecific controls (small chassis with one ahuCD3 (PTE-3) or small chassis with ahuCD19 only (PTE-19)) induced minimal target cell lysis, corroborating the specificity of the designed constructs (Fig. 2d). In parallel, we also monitored the activation of CD8^+^ T cells by analysing the CD69 expression (Supplementary Fig. 13b). Again, only the bispecific construct activated the T cells, whereas PTE-3 only showed low background activation at high concentrations. The lysis efficacy and T-cell activation efficiency of our small chassis variant at 1 nM concentration and above were comparable with an huCD19 × huCD3 BiTe (blinatumomab-biosimilar (Blina-BS), InvivoGen). Blina-BS showed a superior lysis efficacy at concentrations below 0.1 nM relative to our compounds. We attribute the potency differences primarily to different affinities of the antibody paratopes used in our construct relative to those in the BiTe molecule. To test the transferability of the concept to other antigens, we tested the PTE variants against huCD33, huEpCAM, huPSMA and muCD19 in cytotoxic T-cell assays, with tumour cells expressing the respective antigens. To this end, PTE-2×33-3 were tested in co-cultures with acute myeloid leukaemia cell line CD33^+^ MOLM-13, whereas PTE-2×PSMA-3 or PTE-2×EpCAM-3 were analysed in co-cultures with PSMA^+^ LNCaP prostate cancer or EpCAM^+^ MCF-7 breast cancer cells, respectively (Fig. 2e–g). All the PTE variants induced the dose-dependent lysis of target cells with high lysis efficacy in the low nanomolar range. To evaluate the transferability of our approach to murine surrogate molecules, mPTE-3×mCD19-3 were tested in the co-cultures of murine splenocytes with mCD19^+^ A20 cells (Fig. 2h). Using live-cell imaging, we directly observed the specific T-cell-mediated lysis of A20 cells for the bispecific molecule (Fig. 2i).

Finally, we used the smaller F(ab)-based chassis to evaluate our initial findings with the larger IgG-based chassis. To study how the target-cell-binding valency impacts the lysis efficacy of our smaller chassis, we assembled and tested the PTE variants with up to four ahuCD19 F(ab) fragments and one ahuCD3 F(ab) fragment in cytotoxic T-cell assays (Supplementary Fig. 14). The potency increased substantially from one to two aCD19 F(ab) fragments (1,900 pM versus 97 pM). Using more than two aCD19 F(ab) fragments did not improve the potency.

## Biodistribution of PTEs

To ensure PTE stability under physiological low-ionic-strength conditions and achieve protection against nuclease degradation, PTEs were coated with PEG-oligolysine^22^. We validated the successful assembly of PTEs in combination with PEG-oligolysine coating via mass photometry (Supplementary Fig. 15). These PTEs showed storage stability at 4 °C over at least three months with no sign of degradation or loss of functionality in T-cell-mediated lysis assays (Supplementary Fig. 16). Incubation in fetal bovine serum (FBS) (100% serum concentration) for four hours followed by a 24 h T-cell-mediated lysis assay (50% serum concentration) of NALM-6 cells showed comparable results to samples incubated with heat-inactivated FBS (Supplementary Fig. 17). In contrast, PTE samples without a coating showed a substantial decrease in lysis efficiency. We also analysed the composition of the solvent regarding impurities such as endotoxins that may cause immunological side effects in vivo. All the administered samples were below 36 EU ml^–1^ for a daily dose of 100 µl, satisfying the standard residual concentration of endotoxins for in vivo applications (Supplementary Methods)^42^.

**Fig. 3:**
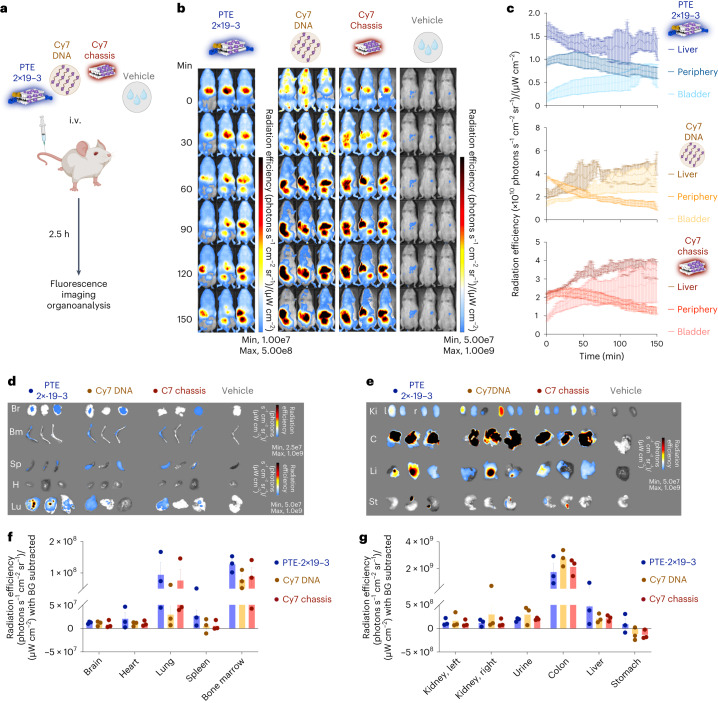
In vivo characterization of DNA-origami-based T-cell engager. **a**, Schematic of the experimental procedure. NSG mice were administered the highlighted samples carrying a Cy7 dye via a tail-vein injection. Distribution of the administered sample was then continuously measured using an in vivo imaging platform (IVIS). Colour scheme, bispecific PTE with Cy7 (midnight; PTE-2×19-3), DNA oligo with Cy7 (mocha; Cy7 DNA), DNA origami chassis with Cy7 (dark red; Cy7 chassis) and vehicle (grey; PBS). **b**, IVIS fluorescent images of NSG mice over time. Left to right, samples as indicated in **a**. **c**, Fluorescence intensity of the bispecific (bluish), Cy7 DNA (brownish) and Cy7 chassis (reddish) samples as a function of time across different organs/regions. Data are mean ± standard error of mean (s.e.m.) from three independent measurements of *n* = 3 mice. **d**,**e**, IVIS fluorescence images of different organs and excreta extracted from *n* = 3 NSG mice. **f**,**g**, Fluorescence intensity in different organs and excreta. The background fluorescence of each organ was subtracted. Data are mean ± s.e.m. from *n* = 3 mice. Source data

We used in vivo time-resolved fluorescence imaging to measure the distribution of PTEs in tumour-free immunodeficient NOD-SCID IL-2Ry-null (NSG) mice. Mice received one intravenous (i.v.) injection (15 pmol) of either phosphate-buffered saline (PBS), DNA chassis or PTE-2×19-3, with fluorescent Cy7 dyes incorporated into the DNA chassis or a mixture of 20 Cy7-modified DNA strands only (here termed Cy7-strand) (Fig. 3a). After injection of the samples, we measured Cy7 fluorescence in the anaesthetized state every 30 s for a duration of 2.5 h (Fig. 3b). Mice treated with the Cy7-dye-containing samples (DNA chassis, PTE-2×19-3 and Cy7-strand) had an increased fluorescence radiance signal compared with the vehicle control.

Five minutes after i.v. injection, the Cy7 signal was delocalized across the mice with a slight accumulation at the liver for all the samples. The fluorescence signal then gradually localized to the liver and to the bladder regions. Accumulation in the bladder region was observed more quickly in the mice that received Cy7-strands or DNA chassis controls compared with PTE-2×19-3-treated mice. We analysed the Cy7 intensity of the liver, bladder and periphery versus time for each variant (Fig. 3c). Overall, traces indicate that the Cy7-strands and DNA chassis controls accumulate in the bladder and the liver regions, whereas PTE-2×19-3 mainly localized in the liver region. Mice given the Cy7 control treatment cleared out faster at the periphery than those administered PTE-2×19-3. We analysed the distribution of the samples in the harvested organs at the end of the experiment (Fig. 3d–g). In accordance with the in vivo imaging results, the strongest Cy7 signal was detected in the colon, liver and kidneys (Fig. 3e,g). However, the Cy7 signal was also measurable in the brain, heart, lungs, spleen and bone marrow (Fig. 3d,f). These results are consistent with previous studies^43,44^ and indicate that DNA-origami-based PTEs readily distribute in living animals and are primarily eliminated through the biliary and renal excretory systems.

## In vivo characterization of PTEs

**Fig. 4:**
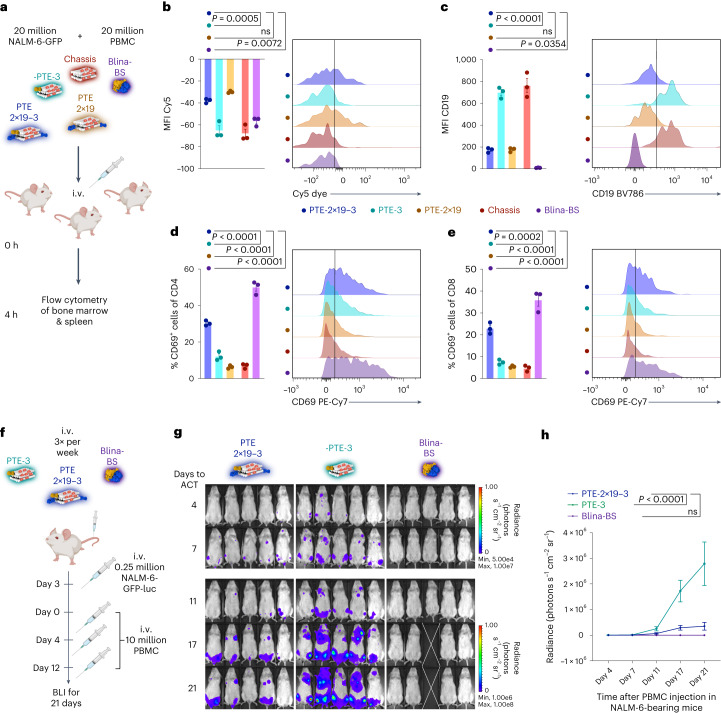
Investigation of functionality of DNA-origami-based T-cell engagers in vivo. **a**, Treatment schedule of binding and activation trials. Experimental procedure to measure the in vivo binding and activation behaviour on tumour (NALM-6) and T cells. Colour scheme: midnight blue, PTE-2×19-3; teal, PTE-3; mocha, PTE-2×19; red, chassis; violet, Blina-BS. Doses were between 10 and 15 pmol. NALM-6-GFP-luc, PBMC and samples were intravenously administered at the indicated time points. Three mice were injected per group. After 4 h, the mice were sacrificed and sample distribution in the bone marrow was analysed using flow cytometry. **b**,**c**, Mean fluorescence intensity (MFI) measurements (left) and representative histograms (right) of flow cytometric measurements of Cy5 fluorescence (**b**) or CD19-BV785 fluorescence (**c**) on NALM-6-GFP-luc cells (indicating Cy5-labelled PTE bound to the tumour cells). **d**,**e**, Measurement of CD69 fluorescence on either CD4^+^ (**d**) or CD8^+^ (**e**) T cells. In **b**–**e**, data are mean ± s.e.m. from *n* = 3 mice. **f**, Schematic of the long-term treatment with PTEs in vivo. **g**, Luminescence images of mice injected with luciferin to visualize NALM-6-GFP-luc tumour cells. The white cross indicates the mouse that was censored due to non-tumour-related toxicities. **h**, Quantification of bioluminescence measurements. For **g** and **h**, *n* = 5 mice per group; for **h**, mean ± s.e.m. is shown. For all the panels, statistical significance was calculated using ordinary one-way or two-way analysis of variance with Tukey’s multiple comparisons correction. Source data

To target T cells against tumour cells in living organisms, PTEs must specifically bind to and remain bound to the target cells until a T cell is recruited. To investigate these processes, we intravenously injected NSG mice first with NALM-6-GFP-luciferase (luc) target cells, then with PBMC effector cells and finally with a single dose of vehicle (PBS), Cy5-modified PTE variant (PTE-2×19-3, PTE-3 and PTE-2×19) or Blina-BS (Fig. 4a). After 4 h, the mice were sacrificed and cells from the bone marrow were analysed using flow cytometry (Fig. 4b–e). GFP^+^ NALM-6 target cells showed an increased Cy5 fluorescence signal only in mice receiving PTE variants that carry ahuCD19 F(ab) fragments (PTE-2×19-3 and PTE-2×19) (Fig. 4b). In addition, we probed the CD19 antigen occupancy by staining the cells with an anti-CD19 antibody before flow cytometric analysis. NALM-6 cells from the vehicle and PTE-3 samples had an increased CD19 signal, indicating the accessibility of the CD19 antigen (Fig. 4c). In contrast, the signal of the anti-CD19 antibody was reduced in mice treated with PTE-2×19-3, PTE-2×19 or Blina-BS (Fig. 4c), indicating that CD19 epitopes are occupied by either PTEs (76% occupied) or Blina-BS (99% occupied), respectively. Overall, these results suggest that PTEs specifically bind to the target cells in vivo and remain bound for several hours (>4 h). As demonstrated in vitro, by crosslinking effector cells and target cells, T-cell engagers recruit and activate the T cells. To validate this mechanism in vivo, we quantified T-cell activation by measuring the expression of CD69 on the transferred T cells as a marker for early T-cell activation (Fig. 4d,e). Both Blina-BS and PTE-2×19-3 induced a significant increase in CD69 expression on CD4^+^ or CD8^+^ T cells compared with the control constructs (Fig. 4d,e). Our results, therefore, indicate that PTEs bind NALM-6 target cells and activate the T cells in vivo.

Next, we studied whether PTEs can also control the tumour outgrowth of NALM-6 target cells in vivo. To this end, we first determined the optimal PTE dose. We administered 20 million NALM-6-GFP-luc cells intravenously and then treated the mice with different concentrations of PTE-2×19-3 (300, 100 and 30 pmol) or control samples (PTE-3, vehicle and Blina-BS) of equivalent doses (Supplementary Fig. 19a). PBMC were intravenously injected on the days of treatment. Bioluminescence (BLI) measurements after six days revealed a significant reduced tumour burden in mice that received PTE-2×19-3 compared with PTE-3- or vehicle-treated mice (Supplementary Fig. 19b,c). Flow cytometric analysis of the bone marrow confirmed the reduced tumour burden for PTE-2×19-3-treated mice and revealed a dose-dependent tumour reduction (Supplementary Fig. 19c). To determine the optimal anti-CD3 clone, we compared the T-cell activation of PTE variants with OKT3 and UCHT1 CD3 binders (Supplementary Fig. 19d). Again, NALM-6-GFP-luc cells were intravenously administered and we injected PBMC and different concentrations of a UCHT1-based 2×19-3 PTE (300 and 30 pmol) or OKT3-based 2×19-3 PTE (30 pmol) or control samples (vehicle and Blina-BS). Flow cytometric analysis of the bone marrow revealed the activation of T cells in all mice that received PTE-2×19-3, with minimal differences between the different CD3 binders (Supplementary Fig. 19e,f). However, mice treated with UCHT1-based PTEs exhibited dose-dependent T-cell depletion compared with mice treated with OKT3-based PTEs (Supplementary Fig. 20).

We then investigated whether OKT3-based PTEs can control tumour outgrowth for an extended duration. We again intravenously injected NALM-6-GFP-luc tumour cells into NSG mice. After three days, mice repeatedly received PBMC and PTE-2×19-3 or PTE-3 and Blina-BS as negative or positive controls, respectively. Tumour growth was subsequently monitored with BLI for a total of 21 days (Fig. 4f). PTE-2×19-3 or Blina-BS treatment led to reduced tumour growth in the treated mice, compared with mice that received PTE-3-negative control samples (Fig. 4g,h). In summary, our studies demonstrate the functionality of PTEs built from DNA origami chassis in vivo.

## Conclusion

DNA origami allows the precise spatial arrangement of biomolecules, and when combined with antibodies, this approach represents an exciting avenue for developing biomedical nanodevices. Here we created a programmable DNA origami chassis that allows the positioning of multiple antibodies and demonstrated the functionality both in vitro and in vivo. By optimizing the attachment strategy as well as the purification and stabilization methods, we demonstrated the ability to assemble and screen a large number of variants in parallel. For example, we assembled 105 multispecific antibody variants to identify and rank antibody combinations for T-cell activation. Such PTEs can target multiple copies of the same antigen and also sets of distinct antigens, allowing for programming more advanced cells or antigen recognition.

The ability to display custom sets of antibodies on DNA origami chassis allowed us to design PTEs that display antibodies against tumour cells and T cells, thereby directing T cells to specifically lyse tumour cells. Although our PTEs and previously developed protein-based aCD19-aCD3 antibodies have comparable efficacies, the PTEs at present require higher concentrations to achieve the same biological effect^45^. The potency differences may be attributed to, for example, different binding modes of the antibody clones used, not-yet-optimal placement of F(ab) fragments on the DNA origami chassis with respect to redirecting T-cell lysis and cellular internalization of the DNA origami PTEs competing with target cell recruitment and lysis^46,47^. The approach may be extended in the future to simultaneously engage several signalling pathways for improved T-cell activation in one molecule. For example, we have also tested combining ahuCD3 with ahuCD28 antibodies (Fig. 1 and Supplementary Fig. 21). The addition of ahuCD28 caused a substantially increased T-cell activation signal for IgG-based PTEs, but only slightly enhanced the potency in our in vitro lysis assays with F(ab)-based PTEs. CD28 co-stimulation may be more beneficial for other tumour models, where the microenvironment and the exhaustion of T cells are important factors for tumour lysis efficacy.

PTEs functioned in vivo and distributed well within animals^22,48^. We showed that our PTEs specifically bind to target cells and recruit T cells in vivo, thus demonstrating the mechanism of action within living animals. In efficacy experiments, our PTEs control tumour outgrowth, demonstrating the applicability of DNA-origami-based therapeutics for cancer treatment.

Given the DNA origami technologies’ modularity, adjustability and high degree of addressability, we expect that a wide range of complex and even logic-gated chassis^10^ for immunotherapy can be engineered. Such chassis have the potential to help overcome important challenges currently faced in the field, including discriminating healthy cells from tumour cells based on surface markers by detecting patterns of multiple antigens rather than on single targets. Additionally, our DNA origami chassis enables the simultaneous engagement of several hallmark signalling pathways for improved T-cell activation in one molecule, including checkpoint inhibition but also targeted co-stimulation. We believe our results will enable the clinical application of DNA nanotechnologies and highlight the potential of DNA-origami-based biomolecular engineering strategies for medical applications.

## Methods

All the experiments and experimental conditions described throughout this study comply with the ethical regulations set forth by the institutional review board of the medical faculty of the Ludwig-Maximilians-Universität and the Regierung von Oberbayern (approval of animal experiments).

### Chemicals, antibodies and cell lines

Unless otherwise mentioned, chemicals used within this work were purchased from Sigma-Aldrich and all the IgG antibodies, RPMI, PBS, FBS and penicillin–streptomycin were purchased from Thermo Fisher.

The human cell lines Jurkat (T-cell leukaemia, DSMZ, no. ACC-282), NALM-6 (B-cell precursor leukaemia, DSMZ, no. ACC-128), MCF-7 (breast adenocarcinoma, DSMZ, no. ACC-115) and Molm-13 (acute myeloid leukaemia, DSMZ, no. ACC 554) were obtained from DMSZ. Human cell line LNCaP (metastatic lesion of prostate adenocarcinoma, CLS, 300265) was obtained from CLS. All the cell lines were stored in liquid nitrogen. Successful cell-line authentication was done via polymerase chain reaction for Jurkat, NALM-6, LNCap and MCF-7.

Jurkat, NALM-6 and Molm-13 cells were grown in RPMI 1640 medium, supplemented with 10% FBS, penicillin (200 U ml^–1^), streptomycin (200 µg ml^–1^) and with or without additional 20 mM l-glutamine for Jurkat and NALM-6, respectively. The MCF-7 cells were grown in high-glucose (25 mM d-glucose) Dulbecco’s modified Eagle’s medium medium, supplemented with 5% FBS, penicillin (200 U ml^–1^), streptomycin (200 µg ml^–1^) and 4 mM l-glutamine and passaged using Trypsin-EDTA (0.05%). Both SK-BR-3 and LNCaP cells were grown in RPMI 1640 medium, supplemented with 20% FBS, penicillin (200 U ml^–1^), streptomycin (200 µg ml^–1^) and 2 mM l-glutamine and passaged using TrypLE Select Enzyme.

The murine A20 cell line was stored in liquid nitrogen and grown in RPMI 1640, supplemented with 10% FBS, penicillin (200 U ml^–1^), streptomycin (200 µg ml^–1^), 200 µM l-glutamine, 25 mM d-glucose, 10 mM HEPES and 1 mM sodium pyruvate.

The cells were cultivated in T-75 cell culture flasks at 37 °C and 5% CO_2_. The cells were maintained according to the instructions from DSMZ and used for flow cytometric and cell-based assays up to a maximum of 15 passages. This cell line was used as a murine B-cell lymphoma model.

For in vivo models, NALM-6 tumour cells were lentivirally transduced with a pCDH-EF1a-eFly-eGFP plasmid as previously described^49,50^. Short tandem repeat profiling was used to verify the origin of this cell line.

For flow cytometric experiments, the following antibodies were used as purchased.

**Table:** 

Antibody	Company
CD8a monoclonal antibody (RPA-T8)	Thermo Fisher
CD69 monoclonal antibody (FN50)	Thermo Fisher
PerCP-eFluor 710 CD4 monoclonal antibody (RPA-T4), PE	Thermo Fisher
CD25 monoclonal antibody (PC61.5), PE-eFluor 610	Thermo Fisher

### Folding of DNA origami objects (chassis)

The reaction mixtures contained scaffold DNA at a concentration of 50 nM and oligonucleotide strands at 200 nM each. The reaction buffer included 5 mM Tris, 1 mM EDTA, 5 mM NaCl (pH 8) and 20 mM MgCl_2_. The reaction mixtures were subjected to a thermal annealing ramp using Tetrad (MJ Research, now Bio-Rad) thermal cycling devices. Oligonucleotides were obtained from IDT. DNA scaffolds were produced in-house according to another work^51^. The table below shows the folding ramps used to assemble the objects described in the manuscript.

**Table:** 

Object	Scaffold type	Highest temperature for 30 min (°C)	Ramp	Incubation temperature (°C)
Medium brick	8,064	65	55 to 50; 1 °C/1 h	25
Small brick	1,033	65	56 to 54; 1 °C/2 h	25

### Gel electrophoresis of PTEs

Folded DNA nanostructures were electrophoresed on 1.5% to 3.5% agarose gels containing 0.5× TBE and MgCl_2_ at different concentrations for around 2 h at 70 V bias voltage in a gel box immersed in a water bath, unless specified otherwise. The electrophoresed agarose gels were scanned using a Typhoon FLA 9500 laser scanner (GE Healthcare) at 50 µm per pixel resolution. The resulting 16-bit TIFF images were analysed using ImageJ v. 1.440. For each lane that contained the sample, a cross-sectional intensity profile was calculated by averaging over grey-scale values within a 50-pixel-wide box. The peak intensities of the monomers and higher-ordered bands were determined in the target band. These intensity values have been used for further analysis.

### Purification, enrichment and in vitro stabilization of PTEs

After the folding reaction, all the reaction products were purified using PEG precipitation^52^. For concentrating DNA origami objects (chassis or PTEs), PEG precipitation or ultrafiltration was used. All the procedures were performed as previously described^53^. Concentrations of DNA origami objects were analysed with a Nanodrop 8000 instrument (Thermo Fisher). Before using the objects in cell culture assays, the objects were stabilized for use in low-ionic-strength buffers and the presence of nucleases. To that end, we used the protocol from another work^22^ and coated all our structures with K10-PEG oligolysine, purchased from Alamanda Polymers.

### Antibody–DNA conjugation, antibody digestion and attachment to DNA origami objects

Conjugation of full-sized IgG antibodies: oligonucleotides modified with 5’- or a 3’-thiol modification were purchased, HPLC purified and dried (Biomers). The oligos were dissolved in PBS (100 mM NaPi, 150 mM NaCl, pH 7.2) with 5 mM TCEP and incubated for 1 h at RT. After purification, 10 nmol of the reduced thiol oligo was mixed with 10 equivalents of sulfosuccinimidyl 4-(*N*-maleimidomethyl) cyclohexane-1-carboxylate (Sulfo-SMCC; dissolved in ddH_2_O) (Thermo Fisher) for 15 min. After purification, including buffer change to PBS (pH 8), 100 µg of antibody in PBS (pH 8) was added. The conjugate was subsequently purified by ion exchange chromatography (proFIRE, Dynamic Biosensors) using a NaCl gradient of 150–1,000 mM in PBS (pH 7.2). Purified oligo–antibody conjugates were analysed by sodium dodecyl sulfate–polyacrylamide gel electrophoresis and agarose gel electrophoresis.

Preparation of Fab fragments: full-sized IgGs were digested and purified using the Pierce Fab Preparation Kit (Thermo Fisher, 44985) according to the supplier’s manual. Briefly, IgGs were incubated with papain beads and purified from Fc fragments using Protein-A affinity beads. Fab generation was checked using sodium dodecyl sulfate–polyacrylamide gel electrophoresis.

Conjugation of Fab fragments: to avoid an orientation where the F(ab)’s paratope points towards the DNA origami chassis, we relied on a site-specific conjugation method similar to another work^54^. In brief, maleimide-modified DNA was purchased from Biomers or prepared by mixing amine-modified DNA with an SMCC crosslinker and subsequent ultracentrifugation (10k filters, Amicon). Fab fragments, which were produced using the papaine digestion of IgG (Pierce Fab Preparation Kit, Thermo Fisher, 44985) were reduced with 5 mM TCEP for 30 min. Excess TCEP was removed using ultracentrifugation (10k filters, Amicon), and mixed with maleimide-modified DNA strands in 50 mM HEPES with 200 mM NaCl at pH 6.7. Reactions were performed overnight at room temperature. This conjugation technique results in single- and double-labelled Fab fragments. Single-labelled F(ab)–DNA conjugates were purified using ion exchange chromatography.

Conjugation of scFv: maleimide-modified DNA was purchased from Biomers or prepared by mixing amine-modified DNA with SMCC crosslinker. scFv contained a free N-terminal cysteine and were expressed and purchased from Genscript or Icosagen. The reaction was carried out as described for the Fab fragments.

Attachment of antibody–DNA conjugates to DNA origami objects: antibody–DNA conjugates and DNA origami objects with the corresponding binding sites were incubated in equimolar ratios for 1 h at 37 °C.

### Negative-stain TEM

#### Preparation, acquisition and data processing

Purified reaction products were adsorbed on glow-discharged Cu400 TEM grids (Science Services) and stained using a 2% aqueous uranyl formate solution containing 25 mM sodium hydroxide. The samples were incubated for 30 s at 20–25 mM Mg^2+^. Magnifications between ×10,000 to ×30,000 were used for acquiring the data.

Imaging was performed on different microscopes.

**Table:** 

Microscope	Operating voltage (kV)	Camera	Objects
Philips CM 100	100	AMT 4 megapixel charge-coupled device camera	Bricks S, M
FEI Tecnai 120	120	TemCam F416 (4k × 4k)	Bricks S, M

TEM micrographs used in the figures were high-pass filtered to remove long-range staining gradients and the contrast was auto-levelled (Adobe Photoshop CS6).

### Fluorescence microscopy

Fluorescence microscopy experiments were obtained on a Thermo Fisher CX7 confocal microscope with an on-stage incubator. Incubation conditions for time-resolved measurements were identical to the cell culture conditions used for the respective cell lines. The samples were incubated on 96-well ibidi plates (89626).

### Cell-based assays

#### Cell surface binding experiments

For flow cytometric experiments, the cells were grown to a cell density of 1.5–2.0 × 10^6^ cells ml^−1^ in T-75 cell culture flasks. The cells were centrifuged for 5 min at 160×*g* and washed with ice-cold PBS, twice. All the flow cytometric experiments were executed at a cell density of 2 × 10^7^ cells ml^−1^ in PBS or medium. The sample (chassis or PTE) was added at a final concentration of 1 nM and incubated for the different time points. Before flow cytometric analysis, the cells were centrifuged for 5 min at 500×*g* and resuspended to a final cell concentration of 2 × 10^6^ cells ml^−1^ in PBS. Flow cytometric analysis was performed on a Cytoflex (Beckman Coulter) or Attune Nxt (Thermo Fisher), measuring the fluorescent intensity by excitation at 640 nm and band-pass filter detection at 660/20 nm. The single cells were gated based on the forward scatter versus side scatter. For each measurement, fluorescent intensities of 50,000 individual cells were analysed with an in-house MATLAB (R2021b) script.

#### T-cell activation assay

The expression of interleukin 2, as an indicator for T-cell activation, was analysed using T-Cell Activation Bioassays (Promega, J1655)^35^. The experiment was performed according to the supplier’s instructions. Briefly, CD19-expressing target cells (NALM-6) were added to 96-well microtiter plates at a final concentration of 5 × 10^5^ cells ml^−1^. Then, a serial dilution of different samples (in RPMI 1640 medium) was added. In the end, the genetically modified TCR/CD3 effector cells were added at a final concentration of 1.3 × 10^6^ cells ml^−1^. The reaction mixture was incubated for 6 h at 37 °C and 5% CO_2_. The genetically modified effector cells (Jurkat-NFAT) intercellularly express a luciferase if the interleukin 2 promoter is activated. By the addition of the Bio-Glo reagent, which includes a substrate for the luciferase, the luminescence signal is a direct proportional signal for the activation of the TCR/CD3 effector cells, which was analysed in a microtiter plate reader (Clariostar Plus, BMG). Data were normalized and the background signal was corrected.

#### Internalization assay

The internalization assay was performed according to another work^55^. Each DNA origami brick (chassis) carried a fluorescence internalization probe (FIP) comprising a protruding sequence with a terminal-attached Cy5 dye (Supplementary Fig. 4, right inset). This FIP can be quenched using a quenching strand with a complementary sequence to the FIP and an attached Black Hole Quencher-2. On hybridization, Black Hole Quencher-2 quenches the fluorescence of the FIP. Since the quencher strand can only reach the chassis on the cell surface, and not the internalized chassis, the amount of quenched signal is proportional to the amount of surface-exposed chassis.

NALM-6 cells (1 × 10^7^ cells ml^–1^) were incubated with 1 nM chassis for 1 h at 37 °C in the cell culture medium and then washed to remove the excess chassis. After washing, the cells were resuspended in the cell culture medium and incubated at 37 °C. At each time point, a measurement consists of taking a sample and incubating it for 10 min at 4 °C on ice. Half of the sample is incubated without a quencher strand and the other half is incubated with a 100 nM quencher strand. Both samples were incubated for 10 min on ice to allow for quencher hybridization (if added) and to stop internalization. The fraction of the internalized chassis with two anti-CD19 antibodies was calculated from the median fluorescence F as follows.

$$\begin{array}{l}{\mathrm{{Fraction}\,{internalized}}}=\\\frac{{F}_{{{\mathrm{with}}}\,{{\mathrm{antibodies}}},\,{{\mathrm{with}}}\,{\mathrm{{quencher}}}}-{F}_{{{\mathrm{without}}}\,{{\mathrm{antibodies}}},\,{{\mathrm{with}}}\,{{\mathrm{quencher}}}}}{{F}_{{{\mathrm{with}}}\,{{\mathrm{antibodies}}},\,{{\mathrm{without}}}\,{{\mathrm{quencher}}}}-{F}_{{{\mathrm{without}}}\,{{\mathrm{antibodies}}},\,{{\mathrm{without}}}\,{{\mathrm{quencher}}}}}\end{array}$$

#### Cytotoxic T-cell-killing assay of liquid tumour cells

##### Preparation of target cells

Target cells (for example, NALM-6) were fluorescently stained using CellTrace CSFE Cell Proliferation Kit (Thermo Fisher, C34554) according to the manufacturer’s protocol.

##### Preparation of PBMC (effector)

For the cytotoxic T-cell-killing assay, we used frozen human PBMC (STEMCELL, 70025.1 or CTL, CTL-UP1). We handled the PBMC cells according to the supplier’s instruction.

##### Assay

Here 2 × 10^5^ CSFE-stained target cells per millilitre were incubated with 1 × 10^6^ PBMC ml^–1^ in the cell culture medium at 37 °C (5% CO_2_) and the PTEs in different concentrations or without an additional recruiter. Cell fluorescence and scattering intensity were determined using an Attune Nxt flow cytometry with a Cytkick Max autosampler (Thermo Fisher). Residual contaminations, such as salts or endotoxins may cause the non-specific lysis of target cells. These effects are particularly pronounced at high PTE concentrations and depend on the cell type.

#### Cytotoxic T-cell-killing assay of solid tumour cells

Preparation of target cells: target cells (for example, MCF-7) were seeded 24 h in advance to from a confluent monolayer.

Preparation of PBMC (effector): for the cytotoxic T-cell-killing assay, we used frozen human PBMC (STEMCELL, 70025.1 or CTL, CTL-UP1). We handled the PBMC cells according to the supplier’s instruction.

Assay: confluent target cells were incubated with PBMC (E:T 2:1 or 4:1) in cell culture medium at 37 °C (5% CO_2_) and the PTEs in different concentrations or without an additional recruiter. The fraction of alive target cells was determined by quantifying the amount of ATP after 48 h via Bio-Glo Cell Titer System (Promega) of samples with and without PTEs. Residual contaminations, such as salts or endotoxins, may cause the non-specific lysis of target cells. These effects are particularly pronounced at high PTE concentrations and depend on the cell type.

### In vivo animal models

#### Endotoxin determination of in vivo ready constructs

The endotoxin concentration was measured with a Charles River nexgen-PTS150 V10.2.3 instrument. We used cartridges with a range between 5.00 and 0.05 EU ml^–1^. The samples were diluted 25-fold to fit into the sensitive range of the cartridges. The endotoxin threshold level for mouse studies was set to 36 EU ml^–1^. This value is in accordance with the specifications given by the FDA^42^.

**Table:** 

Sample	EU measured	EU in sample (25-fold)	QC passed
1-0 (origami)	0.068	1.7	Yes
0-2 (origami)	0.068	1.7	Yes
1-2 (origami)	0.083	2.0	Yes

#### In vivo mouse experiments

Approval for all the animal experiments was granted by the local regulatory authorities (Regierung von Oberbayern).

NOD.Cg-Prkdc^scid^ Il2rg^tm1WjI/SzJ^ (NSG) were purchased from Janvier or bred in-house. NSG mice carry a mutation in the Pkrdc DNA repair gene, associated with severe combined immunodeficiency, leading to a T- and B-cell deficiency. The complete null allele of the IL-2 receptor gamma chain (ll2rg) abrogates the cytokine signalling of critical homoeostatic cytokines such as IL-2, IL-4, IL-7, IL-9 and IL-15, preventing the development of functional NK cells. Finally, the NOD background further compromises the innate branch of the immune system (reduced functionality of dendritic cells and macrophages). In general, these highly immunodeficient mice support stable and reproducible engraftment of human tumour and T cells in mice and are currently regarded as the state-of-the-art model for human xenograft models in mice^56–58^.

All the animals were housed in specific pathogen-free facilities.

BLI and fluorescence imaging were carried out using the in vivo imaging platform Lumina X5 (IVIS, PerkinElmer), as previously described^59^. In brief, mice were anaesthetized with a 1.5–2.5% isoflurane–oxygen mixture for all the live-imaging procedures. For BLI, the substrate (Xenolight d-luciferin potassium salt, PerkinElmer) was intraperitoneally injected according to the manufacturer’s instructions. For organ analysis, the background fluorescence of each organ was subtracted.

NALM-6-luc^+^-GFP^+^ xenograft models were established by i.v. tail-vein injection.

No data points (mice) were excluded in the animal studies.

### Flow cytometry for in vivo experiments

Flow cytometric data were generated using a BD LSRFortessa II, a Beckman Coulter CytoFLEX LX or a Thermo Fisher Attune Nxt with an autoloader. Flow cytometric analysis of organs was conducted as previously reported^60^. Single-cell suspensions of harvested organs were stained with human anti-CD3 BV711 (clone, OKT3), anti-CD4 PerCP-Cy5.5 (clone, OKT4), anti-CD8 PE (clone, HIT8a), anti-CD19 BV786 (clone, HIB19) and anti-CD69 PE-Cy7 (clone, FN50) or mouse anti-CD45 pacific blue (clone, 30-F11) antibodies (Biolegend). Fixable viability dye (eFluor 780, eBioscience) was used to exclude the dead cells. The maximum tumour burden permitted by the local regulatory authorities was not exceeded.

#### Software and statistical analyses

Flow cytometric data were analysed using FlowJo v. 10.3 to v. 10.8.1 software. Quantifying bioluminescence and fluorescence intensities was done using Living Image 4.4 (PerkinElmer). Statistical analysis was carried out with GraphPad Prism v. 9.4.0. Power calculations (for in vivo experiments) were carried using G*Power 3.1 with given alpha, power and effect size.

### Statistics and reproducibility

Statistics and reproducibility are stated in the legend of the figures, for example, biological or technical replicates. Agarose gel images shown in the figures are representative examples of experiments that yielded the same or similar results. For TEM analysis: the total number of similarly conducted TEM analysis of samples prepared following the same protocols varied between experiments—in case of replicates, reproducibility was observed.

### Reporting summary

Further information on research design is available in the Nature Portfolio Reporting Summary linked to this article.

## Online content

Any methods, additional references, Nature Portfolio reporting summaries, source data, extended data, supplementary information, acknowledgements, peer review information; details of author contributions and competing interests; and statements of data and code availability are available at 10.1038/s41565-023-01471-7.

### Supplementary information

Supplementary Information Supplementary Notes 1–3, Figs. 1–21, Table 1, Methods and references.

Reporting Summary

Supplementary Data Statistical source data for all the relevant supplementary figures.

### Source data

Source Data Figs. 1 and 2 Source Data Figs. 1 and 2 - Unprocessed gels and EM data.

Source Data Figs. 1–4 Statistical source data.

## Data Availability

All data that support the findings of this study are available within the Article and its Supplementary Information, and available from the corresponding authors on request. Source data are provided with this paper.
