# Supplementary material for: Programmable multispecific DNA-origami-based T-cell engagers
Source: Nat Nanotechnol. 2023 Aug 17;18(11):1319–26. doi: 10.1038/s41565-023-01471-7 (PMC10656288; doi:10.1038/s41565-023-01471-7)

nature nanotechnology

Article https://doi.org/10.1038/s41565-023-01471-7

Programmable multispecific DNA-origami-based T-cell engagers

In the format provided by the authors and unedited

Table of content

Materials and Methods

Supplementary Text 1 to 3

Figs. S1 to S21

Table S1

References

Supplementary Source Data

Materials and Methods

All experiments and experimental conditions described throughout this study comply with the ethical regulations set forth by the institutional review board of the physics department of the Technical University Munich, the institutional review board of the medical faculty of the Ludwig-Maximilians-Universität and the Regierung von Oberbayern (approval of animal experiments).

1. Chemicals, antibodies, and cell lines

Unless otherwise mentioned, chemicals used within this work were purchased from Sigma Aldrich and all IgG antibodies, RPMI, PBS, FBS, and penicillin streptomycin were purchased from ThermoFisher.

The human cell lines Jurkat (T cell leukemia, DSMZ, No. ACC-282), NALM-6 (B cell precursor leukemia, DSMZ, No. ACC-128), MCF-7 (breast adenocarcinoma, DSMZ, No. ACC-115), Molm-13 (acute myeloid leukemia, DSMZ, No. ACC 554) were obtained from the DMSZ. Human cell line LNCaP (metastatic lesion of prostate adenocarcinoma, CLS, 300265) was obtained from CLS. All cell lines were stored in liquid nitrogen. Successful cell line authentication was done via PCR for Jurkat, NALM-6, LNCap and MCF-7.
Jurkat, NALM-6 and Molm-13 cells were grown in RPMI 1640 medium, supplemented with 10% FBS, penicillin (200 U/mL), streptomycin (200 µg/mL), and with or without additional 20 mM L-glutamin for Jurkat and NALM-6, respectively. The MCF-7 cells were grown in high glucose (25 mM D-glucose) DMEM medium, supplemented with 5% FBS, penicillin (200 U/mL), streptomycin (200 µg/mL), and 4 mM L-glutamine and passaged using Trypsin-EDTA (0,05%). Both SK-BR-3 and LNCaP cells were grown in RPMI 1640 medium, supplemented with 20% FBS, penicillin (200 U/mL), streptomycin (200 µg/mL), and 2 mM L-glutamine and passaged using TrypLE Select Enzyme.
The murine A20 cell line was stored in liquid nitrogen and grown in RPMI 1640, supplemented with 10% FBS, penicillin (200 U/mL), streptomycin (200 µg/mL), 200 µM L-glutamin, 25mM D-glucose, 10mM Hepes, 1mM Sodium Pyruvate.
The cells were cultivated in T-75 cell culture flasks at 37 °C and 5% CO_2. The cells were maintained according to the instructions from DSMZ and used for flow cytometry and cell-based assays up to a maximum of 15 passages. This cell line was used as a murine B-cell lymphoma model.
For *in vivo* models, NALM-6 tumor cells were lentivirally transduced with a pCDH-EF1a-eFly-eGFP plasmid as previously described [1,2]. STR profiling was used to verify the origin of this cell line.

Antibody	Clon	Vendor	ID
CD3	UTCH1	ThermoFisher	16-0038-85
CD3	OKT3	Biozol	BE0001-2
CD3	OKT3	Icosagen	Custom - scFv
CD19	HIB19	ThermoFisher	16-0199-85
CD123	6H6	ThermoFisher	14-1239-37
CD22	eBio4KB128 (4KB128)	ThermoFisher	14-0229-82
CD33	WM-53	ThermoFisher	14-0338-37
CD28	CD28.2	ThermoFisher	16-0289-85
CD137	5G11	ThermoFisher	14-9056-82
muCD19	1D3	Biozol	BE0150
muCD3 F(ab)2	145-2C11	Biozol	711721J1
EpCAM	VU-1D9	ThermoFisher	MA5-12153
PSMA	GCP-05	ThermoFisher	MA1-10335

Table S1: Antibodies used to modify DNA-Origami bricks.

For flow cytometer experiments the following antibodies were used as purchased.

CD8a Monoclonal Antibody (RPA-T8)	ThermoFisher
CD69 Monoclonal Antibody (FN50)	ThermoFisher
PerCP-eFluor™ 710<br>CD4 Monoclonal Antibody (RPA-T4), PE	ThermoFisher
CD25 Monoclonal Antibody (PC61.5), PE-eFluor™ 610	ThermoFisher

2. Folding of DNA-origami objects (chassis)

The reaction mixtures contained scaffold DNA at a concentration of 50 nM and oligonucleotide strands at 200 nM each. The reaction buffer included 5 mM TRIS, 1 mM EDTA, 5 mM NaCl (pH 8) and 20 mM $MgCl_2$. The reaction mixtures were subjected to a thermal annealing ramp using TETRAD (MJ Research, now Biorad) thermal cycling devices. Oligonucleotides were obtained from IDT. DNA scaffolds were produced in-house according to Engelhardt *et al.* [3]. See table below for folding ramps used to assemble the objects described in the manuscript.

**Object**	**Scaffold type**	**Highest temperature for 30 min (°C)**	**Ramp**	**Incubation temperature (°C)**
Medium-brick	8064	65	55 to 50;<br>1°C/1h	25
Small-brick	1033	65	56 to 54<br>1°C/2h	25

3. Gel electrophoresis of PTEs

Folded DNA-nanostructures were electrophoresed on 1.5% to 3.5% agarose gels containing 0.5× TBE and $MgCl_2$ at different concentrations for around 2 h at 70 V bias voltage in a gel box immersed in a water bath if not otherwise specified. The electrophoresed agarose gels

were scanned using a Typhoon FLA 9500 laser scanner (GE Healthcare) at 50 µm/px resolution. The resulting 16-bit tif-images were analyzed using ImageJ 1.440. For each lane that contained sample, a cross-sectional intensity profile was calculated by averaging over greyscale values within a 50-pixel wide box. The peak intensities of monomers and higher-ordered bands were determined in the target band. These intensity values have been used for further analysis.

4. Purification, enrichment, and in vitro stabilization of PTE

After the folding reaction, all reaction products were purified using PEG-precipitation [4]. For concentrating DNA origami objects (chassis or PTEs), PEG precipitation or ultrafiltration was used. All procedures were performed as previously described [5]. Concentrations of DNA-origami objects were analyzed with a Nanodrop 8000 (Thermo Fisher). Before using the objects in cell-culture assays, the objects were stabilized for the use in low-ionic strength buffers and the presence of nucleases. To that end, we used the protocol by Ponnuswamy *et al.* [6] and coated all our structures with K10-PEG oligolysine, purchased from Alamanda Polymers (USA).

5. DNA-Antibody conjugation, antibody digestion, and attachment to DNA-Origami objects

<u>Conjugation of full-sized IgG antibodies</u>: Oligonucleotides modified with a 5' or a 3' thiol modification were purchased HPLC purified and dried by Biomers (Germany). The oligos were dissolved in PBS (100 mM NaPi, 150 mM NaCl, pH 7.2) with 5 mM TCEP and incubated for 1h at RT. After purification, 10 nmol of the reduced thiol oligo was mixed with 10 equivalents of Sulfo-SMCC (sulfosuccinimidyl 4-(N-maleimidomethyl) cyclohexane-1-carboxylate, Thermo, dissolved in ddH_2O) for 15 min. After purification, including buffer change to PBS (pH 8), 100 µg of antibody in PBS (pH 8) was added. The conjugate was subsequently purified by ion exchange chromatography (proFIRE, Dynamic Biosensors) using a NaCl gradient of 150-1000 mM in PBS (pH 7.2). Purified oligo-antibody conjugates were analyzed by SDS-PAGE and agarose gel electrophoresis.

Preparation of Fab fragments: Full-sized IgGs were digested and purified using the Pierce™ Fab Preparation Kit (ThermoFisher, 44985) according to the supplier's manual. Briefly, IgGs were incubated with papain beads and purified from Fc fragments using Protein-A affinity beads. Fab generation was checked using sdsPAGE.

<u>Conjugation of Fab fragments:</u> To avoid an orientation where the F(ab)'s paratope point towards the DNA-origami chassis, we relied on a site-specific conjugation method similar to Humphreys *et al.* [7]. In brief, maleimide-modified DNA was purchased from Biomers or prepared by mixing amine-modified DNA with an SMCC crosslinker and subsequent ultra-centrifiugation (10k amicon). Fab fragments, which where produces using papaine digestion of IgG (Pierce™ Fab Preparation Kit ThermoFisher, 44985) were reduced with 5 mM TCEP for 30 min. Excess TCEP was removed using ultra-centrifugation (Amicon 10k filters), and mixed with maleimide-modified DNA strands in 50 mM HEPES with 200 mM NaCl at pH 6.7. Reactions were performed over night at room temperature. This conjugation technique results in single- and double-labeled Fab fragments. Single-labelled F(ab)-DNA conjugates were purified using ion exchange chromatography.

<u>Conjugation of scFv:</u> Maleimide-modified DNA was purchased from Biomers or prepared by mixing amine-modified DNA with SMCC crosslinker. scFv contained an N-terminal free

cysteine and were expressed and purchased from Genscript or Icosagen. The reaction was carried out as described for the Fab fragments.

Attachment of antibody-DNA conjugates to DNA-origami objects: Antibody-DNA conjugates and DNA-origami objects with the corresponding binding sites were incubated in equimolar ratios for 1h at 37°C.

6. Negative-stain transmission electron microscopy (TEM): Preparation, acquisition, and data processing

Purified reaction products were adsorbed on glow-discharged Cu400 TEM grids (Science Services) and stained using a 2% aqueous uranyl formate solution containing 25 mM sodium hydroxide. Samples were incubated for 30 s at 20-25 mM Mg^{2+}. Magnifications between 10.000x to 30.000x were used for acquiring the data.

Imaging was performed on different microscopes see table below.

Microscope	Operating voltage (kV)	Camera	Objects
Philips CM 100	100	AMT 4 Megapixel CCD camera	Bricks S, M
FEI Tecnai 120	120	TemCam-F416 (4k × 4k)	Bricks S, M

TEM micrographs used in the figures were high-pass filtered to remove long-range staining gradients and the contrast was auto-leveled (Adobe Photoshop CS6).

7. Fluorescence microscopy (FM)

FM experiments were obtained on a ThermoFisher CX7 confocal microscope with an onstage incubator. Incubation conditions for time-resolved measurements were identical to the cell culture conditions used for the respective cell lines. Samples were incubated on 96-well ibidi plates (89626).

8. Cell-based assays

Cell surface binding experiments

For flow cytometry experiments, the cells were grown to a cell density of 1.5-2 $*10^6$ cells ml^{-1} in T-75 cell culture flasks. The cells were centrifuged for 5 min at 160 rcf and washed with ice-cold PBS, twice. All flow cytometry experiments were executed at a cell density of $2*10^7$ cells ml^{-1} in PBS or medium. The sample (Chassis or PTE) was added at a final concentration of 1 nM and incubated for the different time points. Before flow cytometry analysis, the cells were centrifuged for 5 min at 500 rcf and resuspended to a final cell concentration of $2*10^6$ cell ml^{-1} in PBS. Flow cytometric analysis was performed on a Cytoflex (Beckman Coulter) or Attune Nxt (ThermoFisher), measuring the fluorescent intensity by excitation at 640 nm and a bandpass detection filter at 660/20nm. The single cells were gated based on the forward scatter versus the side scatter. For each measurement, fluorescent intensities of 50,000 individual cells were analyzed with an in-house MATLAB script.

T cell activation assay

The expression of interleukin 2, as an indicator for T-cell activation, was analyzed using a T-Cell Activation Bioassays (Promega, J1655)[8]. The experiment was performed according to the instruction of the supplier. Briefly, CD19-expressing target cells (NALM-6) were added to 96 well microtiter plates at a final concentration of $5*10^5$ cells ml^{-1}. Then a serial dilution of the different samples (in RPMI 1640 medium) was added. In the end, the genetically modified TCR/CD3 effector cells were added at a final concentration of $1.3*10^6$ cells ml^{-1}. The reaction mixture was incubated for 6 h at 37 °C and 5% CO_2. The genetically modified effector cells (Jurkat-NFAT) express a luciferase intercellularly if the interleukin 2 promoter is activated. By the addition of Bio-Glo reagent, which includes a substrate for the luciferase, the luminescence signal is a direct proportional signal for activation of the TCR/CD3 effector cells, which was analyzed in a microtiter plate reader (Clariostar Plus, BMG). Data were normalized and the background signal was corrected.

Internalization assay

The internalization assay was performed according to Liu *et al.* [9]. Each DNA-origami brick (chassis) carried a fluorescence internalization probe (FIP) comprising a protruding sequence with a terminal attached Cy5 dye (see inset on the right in Fig. S4). This FIP can be quenched using a quenching strand with a complementary sequence to the FIP and an attached BHQ2 (Black Hole Quencher®-2). Upon hybridization, the BHQ2 quenches the fluorescence of the FIP. Since the quencher strand can only reach chassis on the cell surface, and not internalized chassis, the amount of quenched signal is proportional to the amount of surface exposed chassis.

NALM6 cells (1x10^7 cells/ml) were incubated with 1 nM chassis for 1 h at 37°C in cell-culture medium and then washed to remove excess chassis. After washing, the cells were resuspended in cell-culture medium and incubated at 37°C. At each time point, a measurement consists of taking a sample and incubating it for 10 min at 4°C on ice. Half of the sample is incubated without a quencher strand and the other half is incubated with a 100 nM quencher strand. Both samples were incubated for 10 min on ice to allow for quencher hybridization (if added) and to stop internalization. The fraction of internalized chassis with two anti-CD19 antibodies was calculated from the median fluorescence F as follows

$$Fraction\ internalized = \frac{F_{with\ antibodies,with\ quencher} - F_{without\ antibodies,with\ quencher}}{F_{with\ antibodies,without\ quencher} - F_{without\ antibodies,without\ quencher}}$$

Cytotoxic T-cell killing assay of liquid tumor cells

Preparation of target cells: Target cells (e.g. NALM-6) were fluorescently stained using CellTrace CSFE Cell Proliferation Kit (Thermo Fisher, C34554) according to the manufacturer's protocol.

Preparation of PBMC (Effector): For the cytotoxic T cell killing assay, we used frozen human Peripheral Blood mononuclear cells (PBMC, Stemcell, 70025.1). We handled the PBMC cells according to the supplier's instruction

Assay: 2x10^5 CSFE stained target cells per ml were incubated with 1x10^6 PBMC/ml in the cell-culture medium at 37°C (5% CO_2) and the programmable T-cell engagers in different concentrations or without an additional recruiter. Cell fluorescence and scattering intensity was determined using an Attune Nxt flow cytometry with a Cytkick Max autosampler (ThermoFisher). Residual contaminations, such as salts or endotoxins may cause nonspecific

lysis of target cells. These effects are particularly pronounced at high PTE concentrations and depend on the cell type.

Cytotoxic T-cell killing assay of solid tumor cells

Preparation of target cells: Target cells (e.g. MCF7) were seeded 24h in advance to from a confluent monolayes.

Preparation of PBMC (Effector): For the cytotoxic T cell killing assay, we used frozen human Peripheral Blood mononuclear cells (PBMC, Stemcell, 70025.1). We handled the PBMC cells according to the supplier's instruction

Assay: Confluent target cells were incubated with PBMC (E:T 2:1 or 4:1) in cell-culture medium at 37°C (5% CO_2) and the programmable T-cell engagers in different concentrations or without an additional recruiter. Fraction of alive target cells was determined by quantifying the amount of ATP after 48h via Bio-Glo Cell Titer System (Promega) of samples with and without PTEs. Residual contaminations, such as salts or endotoxins may cause nonspecific lysis of target cells. These effects are particularly pronounced at high PTE concentrations and depend on the cell type.

9. *In-vivo* animal models

Endotoxin determination of in-vivo ready constructs

The endotoxin concentration was measured with a Charles River nexgen PTS150 V10.2.3 instrument. We used cartridges with a range between 5-0,05 EU/ml. Samples were diluted 25-fold to fit into the sensitive range of the cartridges. Endotoxin threshold level for mouse studies was set to 36 EU/ml. This value is an accordance with the specifications given by the FDA [10].

Sample	EU measured	EU in sample (25fold)	QC passed
1-0 (origami)	0,068	1,7	Yes
0-2 (origami)	0,068	1,7	Yes
1-2 (origami)	0,083	2,0	Yes

In-vivo mouse experiments

Approval for all animal experiments was granted by the local regulatory authorities (Regierung von Oberbayern).

NOD.Cg-Prkdc^{scid} Il2rg$^{tm1Wjl/SzJ}$ (NSG) were purchased from Janvier (St Berthevin, France) or bred in-house. NSG mice carry a mutation in the Pkrdc DNA repair gene, associated with the severe combined deficiency syndrom (SCID), leading to a T and B cell deficiency. The complete null allele of the IL-2 receptor gamma chain (Il2rg) abbrogates cytokine signalling of critical homeostatic cytokines such as IL-2, IL-4, IL-7, IL-9 and IL-15, preventing the development of functional NK cells. Finally, the NOD background further compromises the innate branch of the immune system (reduced functionality of dendritic cells and macrophages). In general, these highly immunodeficient mice support stable and reproducible engraftment of human tumor and T cells in mice and are currently regarded as the state-of-the-art model for human xenograft models in mice. [11, 12, 13]

All animals were housed in specific-pathogen-free facilities.

Bioluminescence (BLI) and fluorescence imaging (FLI) was carried out using the *in vivo* imaging platform Lumina X5 (IVIS, PerkinElmer, USA), as previously described [14]. In brief, mice

were anesthetized with a 1.5 – 2.5 % isoflurane-oxygen mixture for all live imaging procedures. For BLI, the substrate (Xenolight D Luciferin potassium salt, PerkinElmer, USA) was injected i.p. according to the manufacturer's instructions. For organ analysis, the background fluorescence of each organ was subtracted.
NALM-6-Luc^{+}-GFP^{+} xenograft models were established by intravenous tail vein injection.
No data points (mice) were excluded in the animal studies.

10. Flow cytometry for *in vivo* experiments

Flow cytometric data was generated using a BD LSR Fortessa II, a Beckman Coulter CytoFLEX LX or a Thermo Fisher Attune Nxt with an autoloader. Flow cytometric analysis of organs was conducted as previously reported [15]. Single-cell suspensions of harvested organs were stained with human anti-CD3 BV711 (clone: OKT3), anti-CD4 PerCP-Cy5.5 (clone: OKT4), anti-CD8 PE (clone: HIT8a), anti-CD19 BV786 (clone: HIB19) and anti-CD69 PE-Cy7 (clone: FN50) or mouse anti-CD45 pacific blue (clone: 30-F11) antibodies (Biolegend, USA). Fixable viability dye (eFluor™ 780, eBioscience, USA) was used to exclude dead cells. The maximum tumor burden permitted by the local regulatory authorities was not exceeded.

Software and statistical analysis

Flow cytometric data were analyzed using FlowJo V10.3 to V10.8.1 software. Quantifying bioluminescence and fluorescence intensities was done using Living Image 4.4 (PerkinElmer, USA). Statistical analysis was carried out with GraphPad Prism V.9.4.0 (San Diego, CA, USA). Power calculations (for *in vivo* experiments) were carried using G*Power 3.1 with given alpha, power and effect size.

11. Statistics and Reproducibility

Statistics and reproducibility are stated in the legend of the figures e.g., biological, or technical replicates. Agarose gel images shown in the figures are representative examples of experiments that yielded the same or similar results. For TEM analysis: the total number of similarly conducted TEM analysis of samples prepared following the same protocols varied between experiments – in case of replicates, reproducibility was observed.

12. Data availability statement

All data and source data that support the findings of this study are available within the paper and its Supplementary Information, and from the corresponding author on request.

Supplementary Text 1:

Antibody position determines cell-binding affinities

A robust and reliable strategy to rationally position IgG antibodies (e.g., anti-CD19 and anti-CD3) at designed sites on a DNA-origami object is the prerequisite for building more complex multivalent configurations. To this end, we covalently modified IgG antibodies with a 25-base NHS-ester activated single-stranded DNA handle (A), where the NHS-ester reacts preferably to lysine residues on the surface of the antibody (Fig. S1a), and purified the DNA-antibody conjugates using ion-exchange chromatography (IEX). By addressing lysines on the surface of the antibody random labeling of the IgG is occurs. Moreover several lysins of one IgG can be modified at once. IEX chromatography allows to separate single-labelled IgGs from IgGs that have more than one DNA strand and from free DNA. As an antibody carrier chassis, we used a previously described and used brick-like DNA-origami object [16]. We placed single-stranded DNA handles (A') on the surface of the DNA brick at different positions. These protruding DNA handles serve as antibody-attachment sites (Fig. S1b) and may be included by the designer in the folding reaction. Finally, a brick-antibody construct is self-assembled by relying on DNA-DNA hybridization of the protruding strand A' with the complementary antibody-conjugated strand A. By using antibody-modifier strands where the NHS-ester modification is at the 5' or 3' end, the antibody can be attached in a distal or in a proximal orientation, respectively (Fig. S1c).

We tested five different antibody positions on the chassis to investigate the influence of the position on the cell-binding behavior. The positions are chosen such that a broad range of 3D accessibility is covered, ranging from the helical interface at the corner (P1) to the middle of a recess on the bottom side of the brick (P5) (Fig. S1c, and Fig. S2). For each position P1 to P5, we screened the distal and proximal antibody orientations and analyzed the attachment efficiency of DNA-antibody conjugates to the brick-like structure by agarose gel electrophoresis (Fig. S1e). The attachment of a full-size IgG anti-CD3 antibody (~150 kDa) to the ~5 MDa brick in comparison to the unmodified brick object resulted in a noticeable migration difference (Fig. S1e). We could also observe a clear migration difference between the proximal and distal-attachment variants and between the different attachment sites. The differences in migration speeds may be explained by the varying hydrodynamic radius of the antibody-brick objects. Analysis of the bands' cross-sectional profiles showed an overall attachment yield up to 95%* (e.g., P1d or P5p). These results were consistent with the TEM data for the P1 position. Single-particle inspection indicated increased flexibility for the distal attachment strategy (Fig. S1f).

The binding affinity between the anti-CD3 antibody and the CD3 antigen on the cell surface of Jurkat T cells was analyzed for ten different fluorescently-labeled brick variants (Fig. S1e). To this end, we performed flow cytometry experiments of brick-cell reaction mixtures and calculated the median fluorescence for each variant of the gated Jurkat cells (Fig. S1g). We also corrected the binding curves for variations in antibody-attachment yields as observed in gel electrophoresis (by correction of the final concentration of particles carrying an antibody). The brick sample with the anti-CD3 antibody showed increased binding to the CD3-positive cells relative to bricks without the anti-CD3 antibody, indicating specific antibody-antigen mediated binding (Fig. S1g, h). DNA-origami bricks with an antibody in the distal configuration showed an increased binding affinity for all attachment positions compared to bricks with an antibody in the proximal arrangement (Fig. S1h). In addition, the cell labeling efficiency varies considerably with the attachment site when comparing only distal or only proximal configurations. For example, the attachment at the corner of the helical interface leads to the highest cell-binding highest mean fluorescent intensity. In contrast, the attachment at the bottom side inside the recess results in the lowest cell binding (Fig S1i). These observations may be explained by the differences in antibody accessibilities at the attachment sites (P1 to P5) and by the increased accessibility of the distal configuration compared to the proximal

configuration. Thus, the better the antibodies' accessibility, the higher the cell-binding affinity. The differences in binding affinities are also consistent with the variations in the migration speed, as higher accessibility corresponds to a lower migration speed. By fitting the binding curves with a simplified binding model (see Supplementary text 2), we could evaluate the binding constant for infinite incubation times. This allowed us to quantitatively compare the affinities of the different positions, revealing differences of one order magnitude. Next, we positioned multiple antibodies to investigate avidity effects (Fig. S3, S4, and S5). With an increasing number of antibodies (e.g., anti-CD19), we see an increase in the on-rate and a decrease in the off-rate (Supplementary text 3 and Fig. S4). We thus conclude that chassis can bind to the cell surface with more than one antibody simultaneously.

* *In general the attachment yield of the antibody to the chassis is limited by the purity of the antibody-DNA conjugate - especially the amount of free handle-DNA, which could block the binding sites on the chassis. The purity varies from batch to batch and strongly depends on the antibody, its format, and the DNA-sequence. For some IgGs we see more residual free DNA than for others. In the case of F(ab) fragments (Fig. 2) there are almost no free DNA molecules in solution. Thus it is important to purify the DNA-antibody conjugate from free DNA-molecules.*

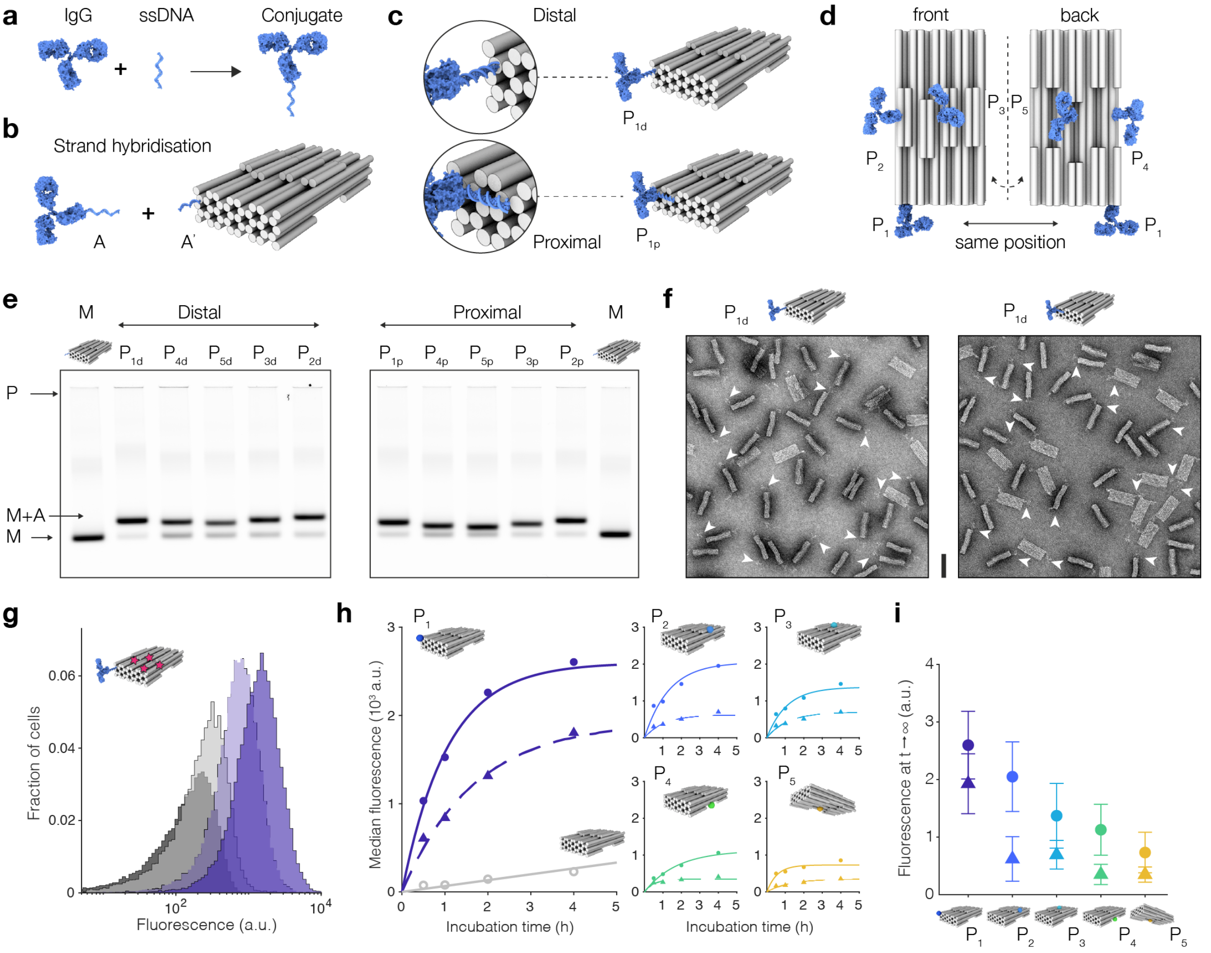

Fig. S1 | Conjugating antibodies to DNA origami chassis and cell binding properties. a to d, Schematics of how IgG antibodies (blue) are attached to sites on a DNA origami brick-like object. **a,** Coupling reaction of ssDNA (blue) to the IgG. Random labeling of the surface lysines on the IgG with DNA – see Supplementary Text 1. **b,** Binding of the antibody-DNA conjugate to the complementary protruding 3'-end sequence (A') on the DNA-origami object. **c,** Schematics of proximal (5' end attached to the IgG) or distal (3' end attached to the IgG)) attachment orientation. **d,** P1 to P5 indicate distinct attachment sites for an anti-CD3 IgG antibody. **e,** Fluorescent laser-

scanned image of a 2% agarose gel on which the unmodified brick (M) as a reference, (left gel) the brick modified with an anti-CD3 antibody in distal attachment at five different attachment sites, and (right gel) the brick modified with an anti-CD3 antibody in proximal attachment at five different attachment sites were electrophoretically separated. P, M+A, and M highlight the pocket of the gel, the DNA origami antibody-conjugate band and the monomer band, respectively. The black arrows indicate the individual bands. **f,** Negatively stained TEM micrographs of the brick-like object modified with an anti-CD3 antibody in distal (left) and proximal (right) attachment. Scale bar, 50 nm. White arrowheads indicate the anti-CD3 IgG antibodies attached to the brick-like object. **g,** Flow cytometry analysis of brick-like structure (fluorescently labeled with 4 Cy5 dyes) binding to CD3 positive T cell leukemia cells (Jurkat). The histograms represent cells only (dark grey), unmodified DNA origami object (grey), anti-CD3 in proximal (light blue) and distal (blue) attachment at position P1 after 2h of incubation. **h,** the Experimentally observed median of fluorescence for different antibody attachment positions (different colors) and configurations (circles: distal; triangles: proximal) determined from histograms as shown in (g) as a function of the incubation time. Solid lines represent fits of a simplified binding model (Supplementary text 2) to the data. The colored sphere in the schematic icons represents the attachment site of anti-CD3 antibody. **i,** Extrapolated saturation intensity as calculated from fits in (h) for proximal (triangle) and distal (circle) attachment at different sites as indicated by the icons on the bottom. Error bars: 95% confidence interval of the fit parameters from h.

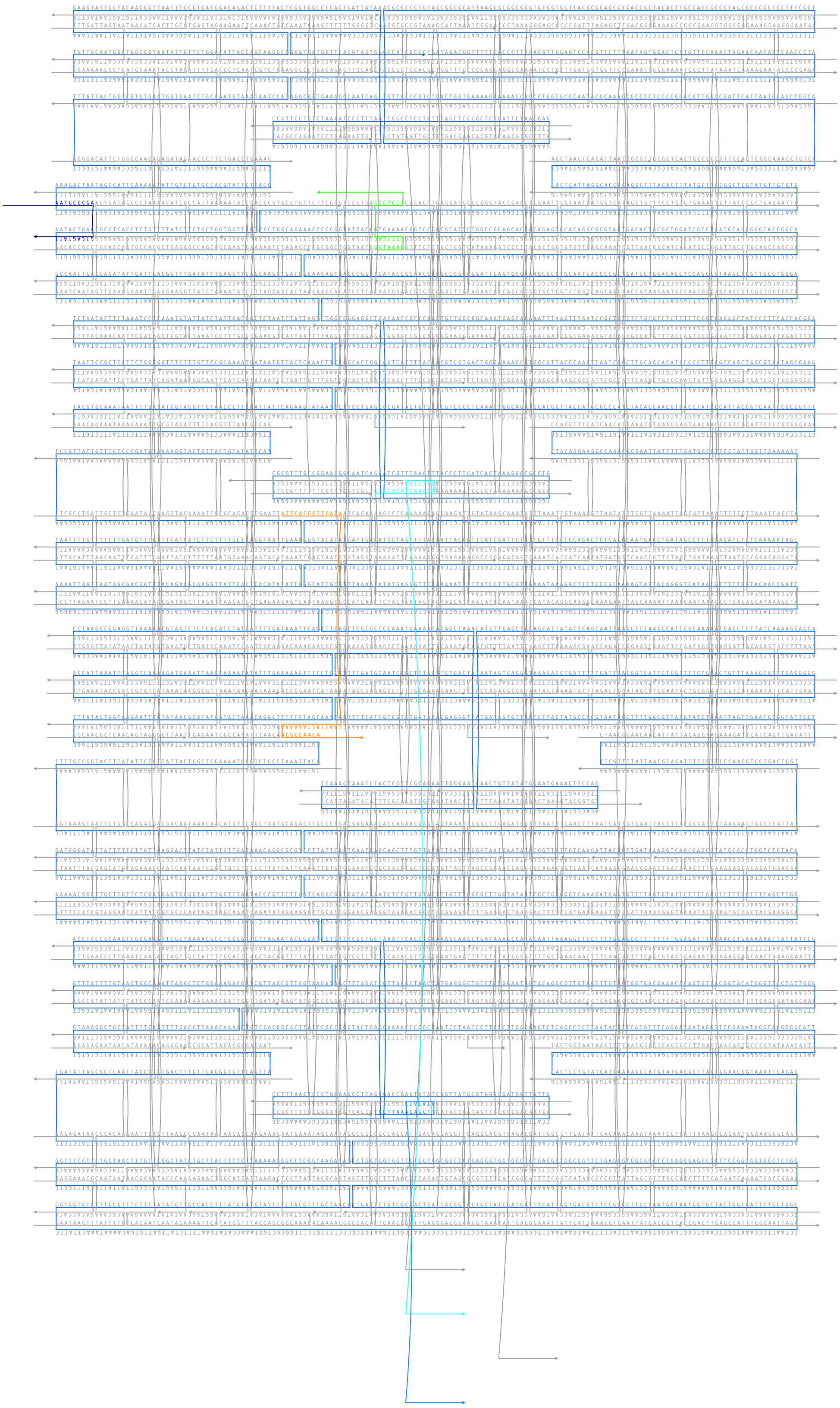

Fig. S2 | Design diagram of the brick prepared using caDNAno [17]. Object is passivated with poly-thymine bases at the helical interfaces. The variant presented here has five different protruding sequences (colored as in Figure 1) at different positions for anti-CD3 attachment in proximal or distal orientation.

Supplementary Text 2:

Fitting binding curves

DNA-origami bricks B, with concentration c_B can bind from solution to surface antigens A, with surface-antigen concentration c_B, forming a brick-antigen complex AB. The rate equations are given by:

$$\dot{c}_A = k_{off}\ c_{AB} - k_{on}\ c_A\ c_B \tag{1}$$

$$\dot{c}_B = k_{off}\ c_{AB} - k_{on}\ c_A\ c_B \tag{2}$$

$$\dot{c}_{AB} = -k_{off}\ c_{AB} + k_{on}\ c_A\ c_B \tag{3}$$

where k_{on} is the rate constant describing the binding of the brick to the antigen and k_{off} is the rate constant describing the unbinding of the brick from the cell. The dot indicates the derivative with respect to the time t.

In order to simply this system of differential equations, we make two assumptions. First, we assume that the concentration of DNA-origami bricks is approximately constant throughout the time frame of an experiment, that is $\dot{c}_B \approx 0$ and $c_B(t) \approx c_B(0)$. This assumption is motivated by the fact that the concentrations of DNA-origami bricks (usually 1 nM) is much higher than the concentration of available antigens ($\sim$ 0.1 nM) $c_B(0) \gg c_A(0)$. Second, we assume that the concentration of free surface antigens is approximately constant throughout the time frame of an experiment, that is $\dot{c}_A \approx 0$ and $c_A(t) \approx c_A(0)$. This approximation is motivated by the fact that the reaction is limited by the slow on-rate of the DNA-origami brick compared to the experimental time frame. We validated this assumption, by adding a fluorescently modified antibody at high concentrations (10-100 nM) to a sample that was previously incubated with a DNA-origami brick (1 nM). This experiment revealed that a large fraction $> 60\%$ of the antigens are available at the end of the experiment.

Using the these two assumptions, the binding term in equation 1 can be assumed to be roughly constant and we replace c_B with $c_B(0)$ and c_A with $c_A(0)$.

$$\dot{c}_{AB} \approx -k_{off}\ c_{AB} + k_{on}\ c_A(0)\ c_B(0)$$

The solution to this differential equation is

$$\dot{c}_{AB}(t) \approx \frac{k_{on}\ c_A(0)\ c_B(0)}{k_{off}}\ \left(1 - e^{-k_{off}\ t}\right)$$

The cell-binding curves $f(t)$ of the DNA-origami brick analyzed using flow cytometry can thus be fitted with $f(t) = c_0(1-\exp(c_1\, t))$, where $c_0 = k_{on}\ c_A(0)\ c_B(0)$ and $c_1 = k_{off}$ are fitting parameters.

For large times , the fitting curve converges to $f(t \to \infty) = c_0 = k_{on}\ c_A(0)\ c_B(0)/k_{off}$, which represents the binding strength multiplied by the brick and antigen concentrations. For small times, we can tailor expand the fitting curves and obtain

$f(t) \approx k_{on}\ c_A(0)\ c_B(0)$, which demonstrates that the initial binding rate depends on the brick concentration, the antigen concentration, and the binding rate constant k_{on}.

The fitted parameters for the screen in Figure 1 are:

Attachment position	attachment strategy	c_0	c_1
1	proximal	1927 ± 520	0.6 ± 0.4
1	distal	2598 ± 588	1 ± 0.6
2	proximal	619 ± 387	0.9 ± 1.7
2	distal	2049 ± 604	1 ± 0.6
3	proximal	693 ± 249	0.9 ± 0.9
3	distal	1372 ± 563	1 ± 1.1
4	proximal	350 ± 175	1.9 ± 3.9
4	distal	1128 ± 442	1 ± 0.5
5	proximal	349 ± 132	0.8 ± 0.9
5	distal	732 ± 352	2 ± 3.4

Fitting unbinding curves

For fitting unbinding curves, we assume that the binding rate vanishes and equation becomes

$$\dot{c}_{AB} \approx -k_{off}\ c_{AB}$$

The solution to this decay is

$$\dot{c}_{AB}(t) = c_{AB}(0)\ e^{-k_{off}\ t}$$

Experimentally, we observed that the unbinding curves did not decay to zero. We hypothesize that this is due to internalization of DNA-origami bricks into cells. TO compensate for this effect, we introduce an offset c_2 and fit the unbinding curves with $f(t) = c_0\ \exp(-c_1\ t) + c_2$.

The fitted binding parameters for the screen in Figure 2 are:

Number of antibodies	c_0	c_1
1	1328 ± 481	2 ± 3.2
2	4898 ± 885	2 ± 0.9
3	6957 ± 1167	1 ± 0.6
4	7385 ± 746	2 ± 0.5

The fitted unbinding parameters for the screen in Figure 2 are:

Number of antibodies	c_2	c_0	c_1
1	492 ± 348	958 ± 468	0.96 ± 1.27
2	1152 ± 2207	3878 ± 2088	0.32 ± 0.34
3	2333 ± 6681	4164 ± 6349	0.28 ± 0.78
4	227 ± 44043	7000 ± 43701	0.09 ± 0.65

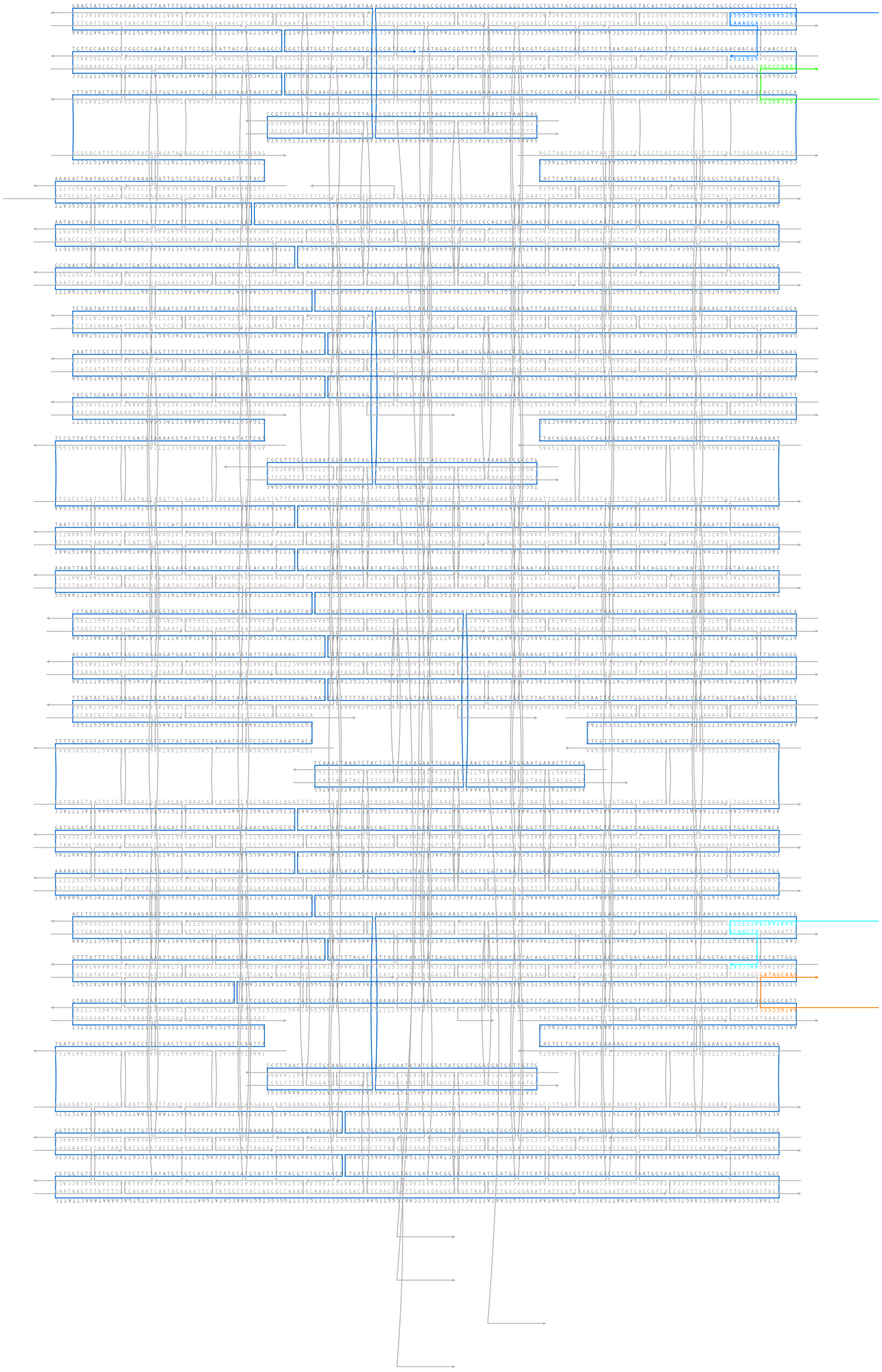

Fig. S3 | Design diagram of a brick variant (chassis) prepared using caDNAno [17]. Object is passivated with poly-thymine bases at the helical interfaces. The variant presented here has four different protruding sequences at the helical interfaces (colored) for multiple anti-CD19 attachments.

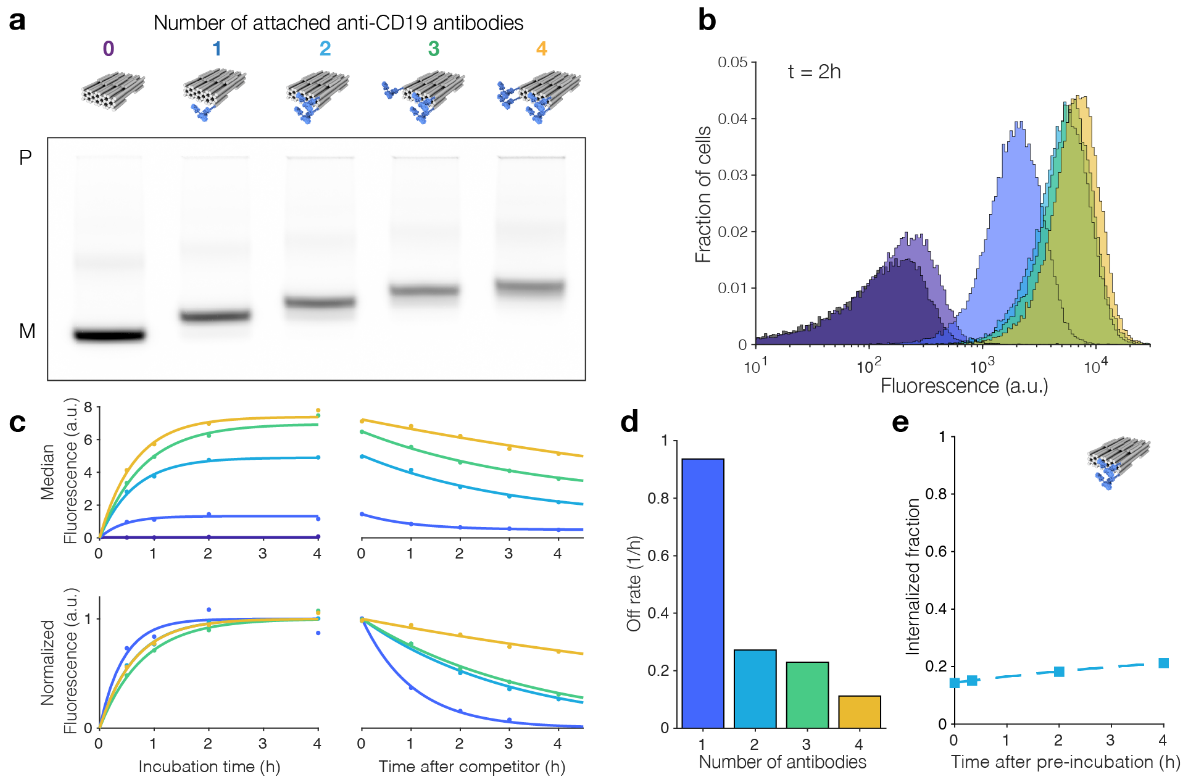

Fig. S4 | Multivalent antibody DNA origami constructs. a, Fluorescent laser-scanned image of a 2% agarose gel on which the unmodified brick (0) as a reference, and bricks with up to 4 attached anti-CD19 antibodies in distal attachment were electrophoretically separated. Colored icons highlight the position and number of anti-CD19 IgG antibodies. P and M highlight the pocket of the gel and the monomer band, respectively. **b,** Flow cytometry analysis of brick-like structure (fluorescently labeled with 4 Cy5 dyes) binding to CD19 positive precursor B cell leukemia cells (NALM-6). The histograms represent the cells only (purple), the unmodified DNA-origami object (dark blue), with one anti-CD19 (blue), two anti-CD19 (light blue), three anti-CD19 (green), and four anti-CD19 (orange) in distal attachment. **c,** Left: experimentally observed median fluorescence (two separate measurements) for different numbers of attached anti-CD19 antibodies (different colors) determined from histograms as shown in (b) as a function of the incubation time. Solid lines represent fits of a simplified binding model to the data. Right: experimentally observed median fluorescence after a competitor (50-fold excess of free anti-CD19 antibody) is added as a function of the incubation time. Solid lines represent fits of an exponential unbinding model to the data. **d,** Calculated off-rate determined from the fits in (c) as a function of the number of attached anti-CD19 antibodies. Inset: normalized fluorescence as a function of the time after the competitor is added. **e,** Internalized fraction of modified DNA origami objects as a function of time after 1h pre-incubation. Icon indicates the double anti-CD19-modified brick.

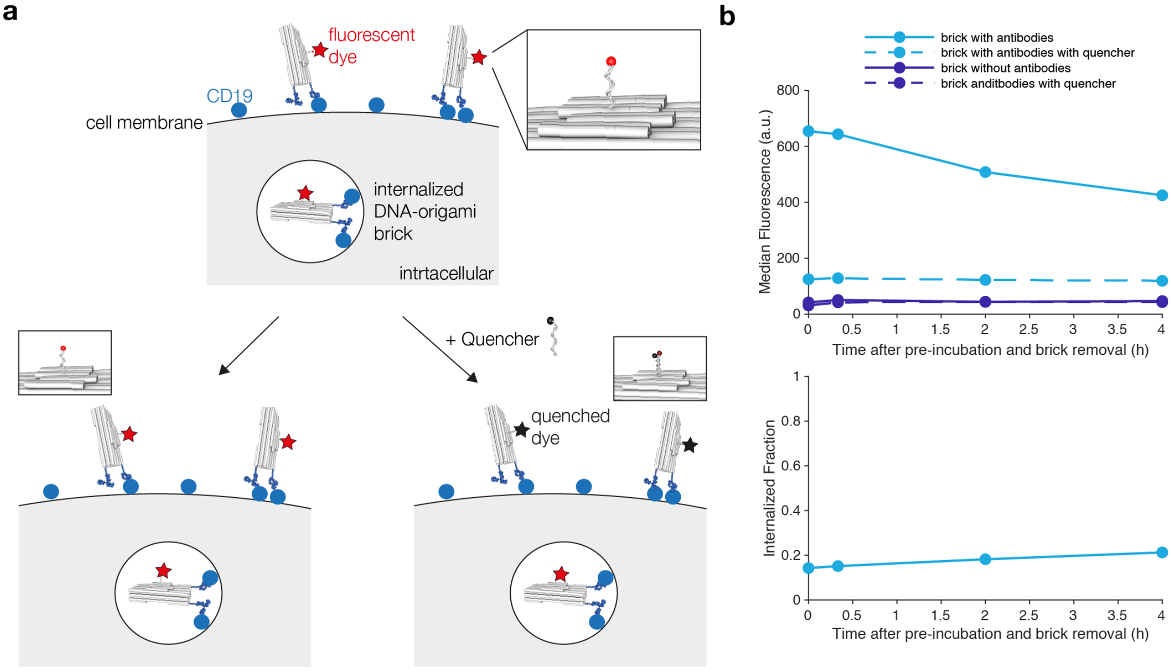

Fig. S5 | Internalization of DNA-origami chassis into NALM-6 cells. a Top: schematic of the DNA-origami bricks (white) with attached anti-CD19 antibodies (blue) that are bound to the cell surface's CD19 antigens and one brick that is internalized in an endosome. The internalization assay was performed according to Liu and Johnston [9]. Each DNA-origami brick carries a fluorescence internalization probe (FIP) comprising a protruding sequence A with a terminal attached Cy5 dye (see inset on the right). Bottom: at each time point, a measurement consists of taking a sample and incubating one half of the sample at 4°C without a quencher strand (left) and one half of the taken sample with 100 nM quencher strand (right, quencher strand is depicted as ssDNA with an attached black sphere). The quencher strand's sequence is complementary to the FIP strand. Upon hybridization, the quencher quenches the fluorescence of the FIP. Since the quencher strand can only reach DNA-bricks on the surface, and not internalized bricks, the amount of quenched signal is proportional to the amount of surface exposed bricks. **b** top: median fluorescence signal from flow cytometry experiments of samples taken at different time points from different brick-cell incubations. Samples were incubated for 1h at 37°C with 1 nM DNA-origami brick and then washed to remove excess DNA-origami brick. Light blue lines: DNA-origami brick with two attached anti-CD19 antibodies; dark blue lines: DNA-origami brick without antibodies. Solid lines are fluorescent signals from samples without quencher and dotted lines are samples where 100 nM quencher was added. Both samples were incubated for 10 min on ice to allow for quencher hybridization (if added) and to stop internalization. Bottom: fraction of internalized DNA-origami bricks with two anti-CD19 antibodies calculated from the median fluorescence F as follows

$$Fraction\ internalized = \frac{F_{with\ antibodies,with\ quencher} - F_{without\ antibodies,with\ quencher}}{F_{with\ antibodies,without\ quencher} - F_{without\ antibodies,without\ quencher}}$$

Supplementary Text 3: Multivalent DNA-origami binders

A standard IgG antibody can bind up to two antigens. In order to exploit multi-valency effects with our constructs, we mounted multiple IgG antibodies on the same DNA-origami brick. Our initial findings showed that the highest binding affinity is achieved at the corner of the DNA brick in a distal configuration. We, therefore, distributed up to four anti-CD19 antibodies to the four corners of the helical interface in a distal configuration (Fig. S3 and S4a). Gel electrophoresis of these constructs revealed defined bands that migrate slower than the unmodified brick band and whose migration speeds decrease with the number of attached antibodies (Fig. S4a). We, therefore, assign these bands to DNA-origami bricks with one to four correctly attached antibodies. We calculated the antibody-attachment yield by cross-sectional profile analysis to 97% for the brick with one antibody and 88% for the brick carrying four anti-CD19 antibodies (Fig. S4a). Occasionally, we also saw lower-molecular weight bands in the gel images below the target lane for brick samples with a designed number of two, three, and four attached antibodies. With the calculated binding efficiency of one antibody, we thus attribute these bands to particles that do not carry all designed antibodies.

We used NALM-6 cells, a suspension cell line derived from a patient with acute lymphoblastic leukemia expressing the antigen CD19, to investigate the binding properties of the multivalent brick constructs. We performed flow cytometry analysis of incubations with NALM-6 cells and fluorescently tagged DNA bricks with up to four anti-CD19 antibodies. The number of bound objects increased with the number of attached antibodies. Brick objects without antibodies caused a slight increase in fluorescence signal compared to the sample containing cells only. The brick variant with one antibody showed, however, a much higher fluorescence signal than the unmodified brick object (1 vs 0, Fig. S4b). By attaching two anti-CD19 antibodies, we again observed an increase in fluorescence signal. This difference gets less for brick variants with three or four attached antibodies (Fig. S4b). After 4h incubation, we observed a ten-fold increase in binding between the variant with one and the variant with four attached antibodies (Fig. S4c, left). It would be expected that the number of antibodies on the DNA-origami brick will increase the binding rate as the effective antibody concentration is larger for objects with more antibodies. It is, therefore, difficult to directly deduce if the brick has bound more than one antigen. To test this multivalent binding, we added free unmodified anti-CD19 antibodies in 50-fold excess over the bricks to each sample (Fig. S4c, right). The free antibody should block detached bricks from re-binding. Thus, allowing us to directly observe the brick detachment process. The rate of brick detachment decreased with the number of anti-CD19 antibodies that were mounted on the brick. In order to quantify the brick's off rate, we fitted an exponential decay model to the unbinding data (Supplementary text 1, Fig. S4d). The highest off-rate was found for the brick carrying only one anti-CD19 antibody, whereas bricks with four antibodies had the slowest off-rate and bricks with two or three antibodies had intermediate off rates. We thus conclude that bricks can bind to the cell surface with more than one antibody. Despite the high off-rate for one attached antibody, we still observed a residual fluorescence signal after four hours of brick removal. This signal might point either toward unspecific interactions with the cell surface or toward cellular uptake of the fluorescently-labeled bricks. To investigate these hypotheses, we performed an internalization assay, as previously described (Fig. S5). Briefly, we included a protruding fluorescence single strand on the brick. The fluorescence is quenched upon hybridization of a complementary strand that carries a quencher molecule. Since the quencher strand cannot penetrate the cell, the amount of quenched fluorescence corresponds to the fraction of brick objects on the cell surface. After four hours, the cells have taken up approximately 20% of the modified bricks (Fig. S4e). The cellular uptake might explain the previously measured residual fluorescent signal.

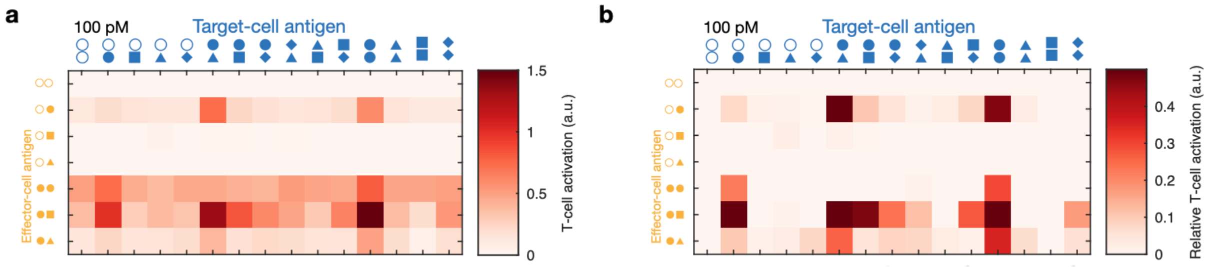

Fig. S6 | Analysis of NFAT T cell activation assay. T cell activation was measured by using NFAT-luciferase Jurkat cell line in co-cultures with human ALL cell lines NALM-6 in the presence or absence of the indicated multi-specific combinations. **a,** Absolute T cell activation signal for variants at 100 pM. Same nomenclature as depicted in Figure 1. **b**, Relative T cell activation signal, obtained by subtracting the signal generated by PTEs that only carried T cell antibodies from the signal generated by PTEs that carry T cell and target-cell antibodies.

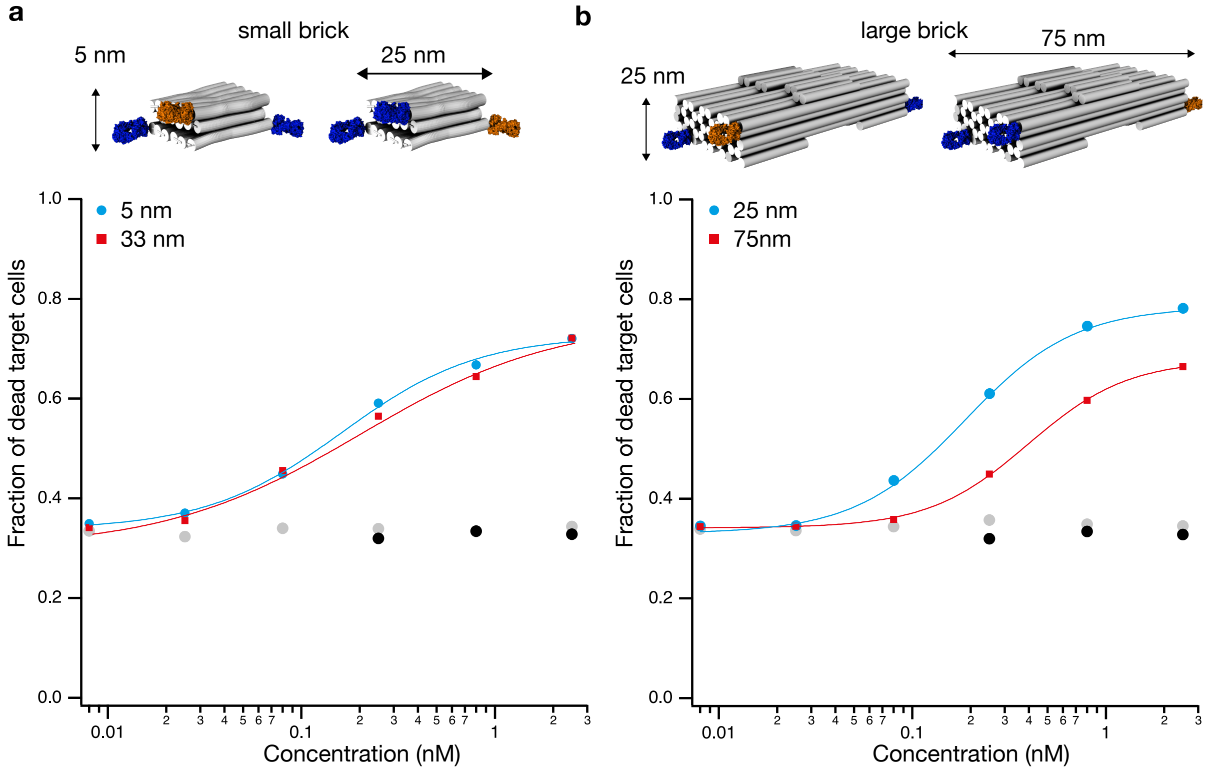

Fig. S7 | Distance dependent T-cell mediated lysis of NALM-6 cells. a, top scheme of the small PTE with 2 aCD19 Fab fragments and one aCD3 Fab fragment mounted at different sites of the brick. aCD3 to aCD19 distance was adjusted to appr. 5 and 33 nm. Low. Concentration-dependent lysis of NALM-6 cells. Grey dots (aCD3 only), black dots (PBMC and NALM-6 cells). Solid lines are Hill-fits to data. **b,** as in a but for the large PTE with different distances between the Fab fragments.

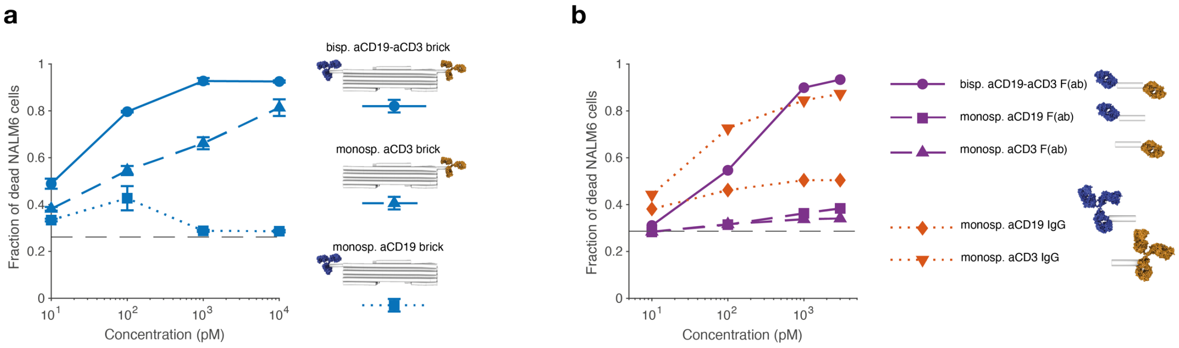

Fig. S8 | Unspecific T cell mediated lysis of full-sized aCD3 IgGs. Fraction of dead NALM6 target cells from cytotoxic T cell mediated target-cell lysis assay after 24 h as a function of the construct concentration in the assay. Effector (Peripheral Blood Mononuclear cells, PBMC) and target cell ratio was chosen to 5:1. **a,** Lysis efficacies for a bispecific brick variant with aCD3 and aCD19 IgGs (solid line, cirlces), for a monospecific brick variants with aCD3 IgG (dashed line, triangle), and for a monospecific brick variant with aCD19 IgG (dotted line, squares). Error bars to the data are standard deviations to the mean of three biological replicates. **b,** Lysis efficacies for a bispecific aCD19-aCD3 construct assembled from two F(ab)-DNA conjugates (purple solid line, cirlces), for monospecific F(ab)-DNA conjugates (purple dashed lines, squares are aCD19 and triangles are aCD3), and for monospecific IgG-DNA conjugates (red dotted lines, diamonds are aCD19 and triangles are aCD3). Black dashed lines indicated fraction of dead NALM6 cells where no construct has been added.

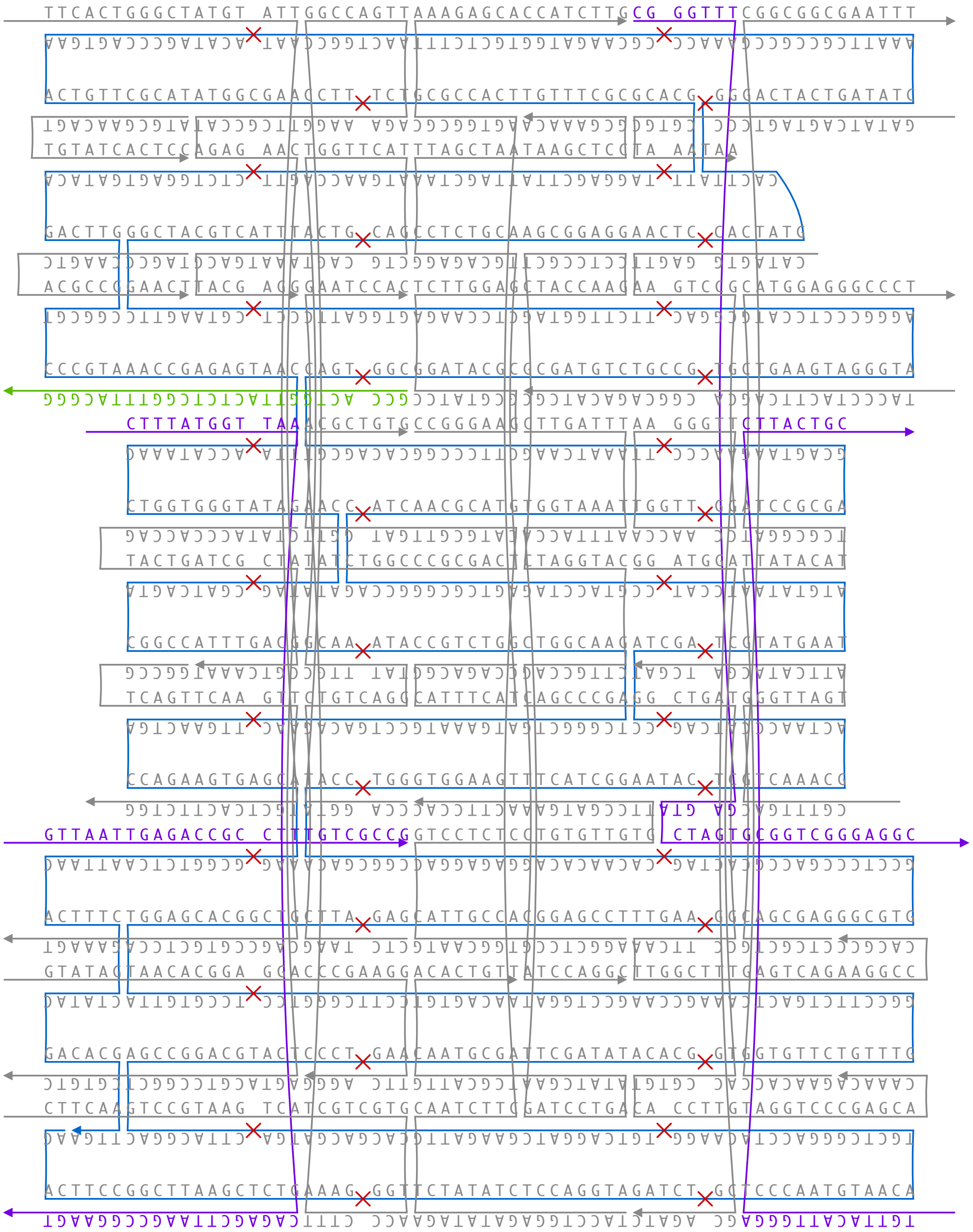

Fig. S9 | Design diagram of small brick variant (chassis) prepared using caDNAno [17]. Object is passivated with poly-thymine bases at the helical interfaces. The variant presented here has one binding site for the T-cell-recruiting antibody (green) and up to four binding-site for the target-cell specific antibody or Fab fragment (violet). This brick variant was used for T-cell mediated killing assays – in vitro and in vivo.

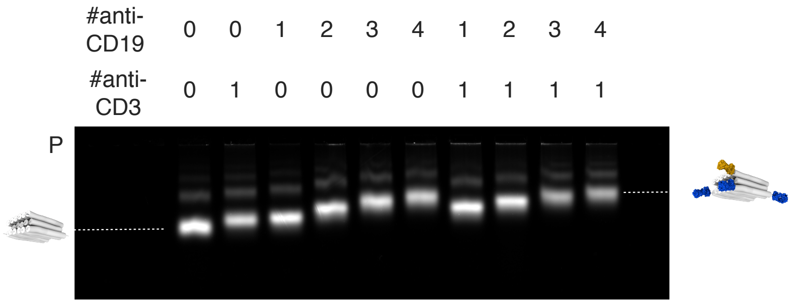

Fig. S10 | Quality control of the PTE. Fluorescent laser-scanned image of a 3.5 % agarose gel on which the chassis (0-0) as a reference (left lane) and different variants of the PTE were electrophoretically separated. The multispecific combination is explained by the table over the gel: the number indicates the numbers of the respective Fab fragments bound to the chassis. P and the icons highlight the pocket of the gel, the DNA-origami antibody-conjugate (with the highlighted number of bound antibodies) band, and the monomer band, respectively. The dashed line indicates the respective bands.

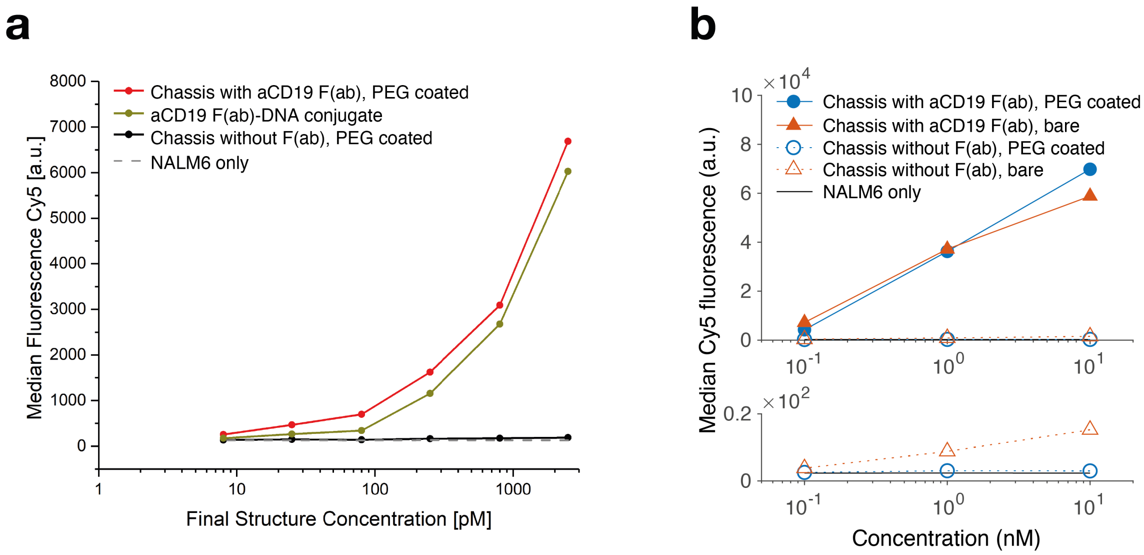

Fig. S11 | Binding affinity of F(ab) is not altered when F(ab) is attached to chassis or when PEG coating is added. Median cy5 fluoresence of NALM6 cells from flow cytometry experiments of NALM6 cell incubated with different cy5-modifed samples. **a,** NALM6 cells were incubated with large brick chassis with one aCD19 F(ab) (red line), without aCD19 F(ab) (black line), or with a F(ab)-DNA conjugate that was hybridized to a complementary Cy5-modified DNA strand (green line). Dashed black line is the fluorescence of NALM6 cells only. **b,** NALM6 cells were incubated with chassis that carried one aCD19 F(ab) fragment (circles) or no F(ab) (triangles) and that were either coateg with K10-PEG-5K oligolysine (blue) or bare (red). Top and botton plots show the same data with different limits of the median Cy5 fluorescence. Black line is the fluorescence of NALM6 cells only.

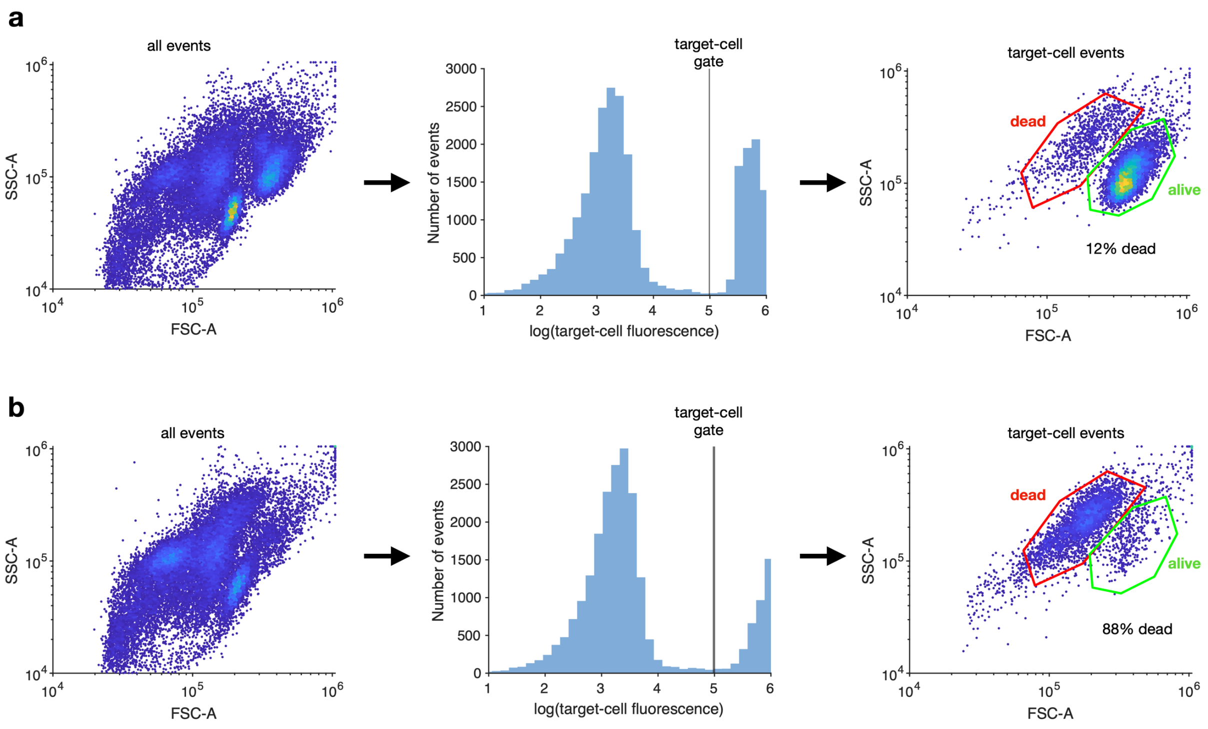

Fig. S12 | Gating strategy for in vitro T-cell mediated lysis assay. NALM6 target cells were stained with CellTrace CSFE and mixed with PBMCs (at 5:1 effector-to-target-cell ratio) and DNA-origami T-cell engagers and incubated at 37°C at 5% CO2. Lysis efficiency was quantified after 24-48 h using flow cytometry. **a,** Exemplary plot of the side- vs. forward-scattering signal of all detected events for a sample containing only target and PBMCs (left). Events corresponding to target cells were gated based on the CSFE-CellTrace fluorescence of the NALM6 target cells (center). Live and dead target cells were selected based on the side vs. forward scattering signal (right, dead-cell population in red, alive-cell population in green). **b,** Same as in a, but for a sample tht contained target cells (NALM6), PBMCs, and 2.5 nM PTE with two anti-CD19 Fabs and one anti-CD3 Fab.

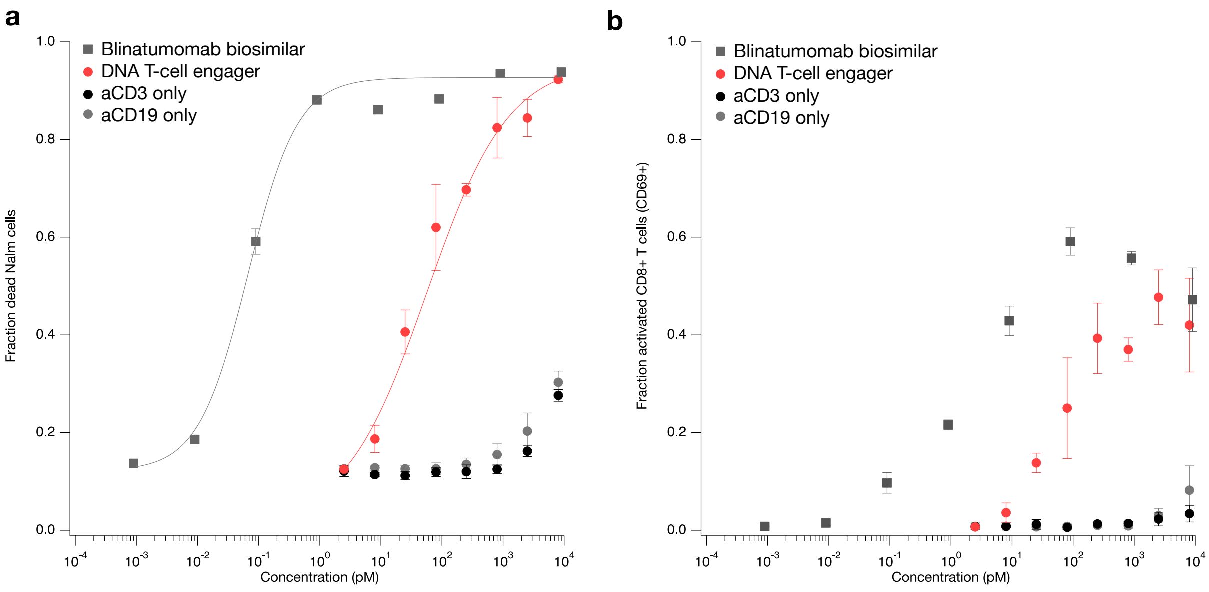

Fig. S13 | Quality control of the in-vivo samples. a, Concentration dependent T-cell (CD8+) mediated lysis of target cells (NALM-6) via PTEs. T-cell-mediated lysis efficiency as a function of the final concentration of the different reactants. red circs indicate the PTE 2x19-3, grey and black circs indicate the PTE -2x19 and PTE-3control objects, respectively. The squares represent the BiTe biosimilar construct Blinatumomab. Solid lines are Hill fits to the respective data sets. **b,** T-cell activation (CD69+) of CD8+ T-cells as a function of the final concentration of the different reactants. For **a, b** Markers are the average of three independent measurements, error bars indicate the mean square deviation from the average lysis efficiency/activation.

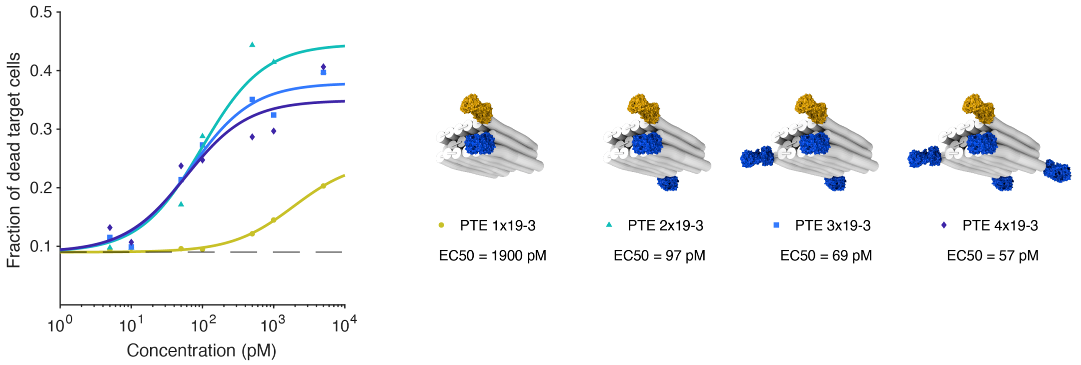

Fig. S14 | Valency screen for F(ab) based chassis. Fraction of dead NALM6 target cells from cytotoxic T cell mediated target-cell lysis assay after 24 h as a function of the construct concentration in the assay. Effector (Peripheral Blood Mononuclear cells, PBMC) and target cell ratio was chosen to 5:1. Lysis efficacies for PTE variants with one aCD3 and one aCD19 F(ab) (green circles), two aCD19 F(ab) (turquise triangles), three aCD19 F(ab) (light blue square), or four aCD19 F(ab) (dark blue diamonds). Data was fitted with Hill-equiation to extract the potency (EC50). Black dashed lines indicates fraction of dead NALM6 cells where no construct has been added.

´

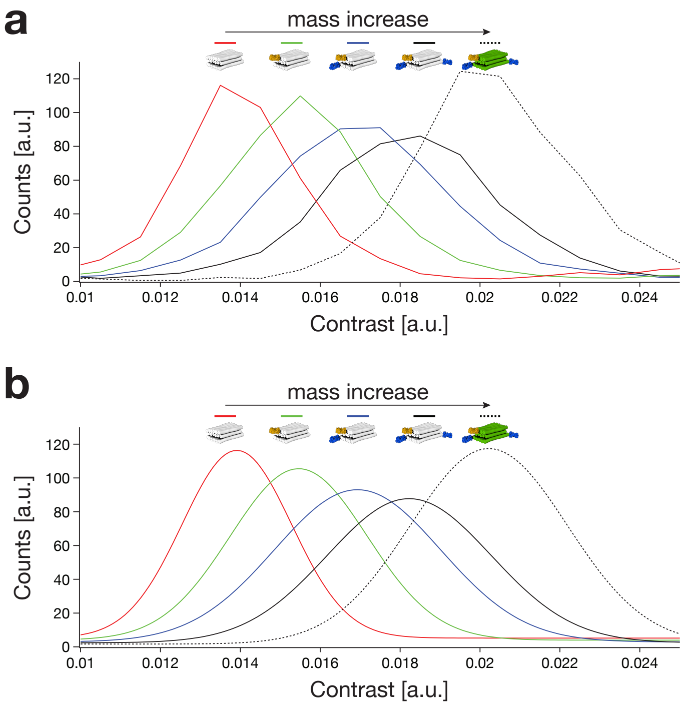

Fig. S15 | Quality control for in-vivo samples via mass spectrometry (ISCAMS) a, Contrast histograms of chassis in 6 mM MgCl2 buffer depending on the number of fab fragments attached (red: no fab fragments, green: 1 fab fragment, blue: 2 fab fragments, black: 3 fab fragments, dashed black: 3 fab fragments with PEG-oligolysine coating). **b,** Gaussian fits of contrast histograms for small brick (red: no fab fragments, green: 1 fab fragment, blue: 2 fab fragments, black: 3 fab fragments, dashed black: 3 fab fragments with PEG-oligolysine coating) [6, 18].

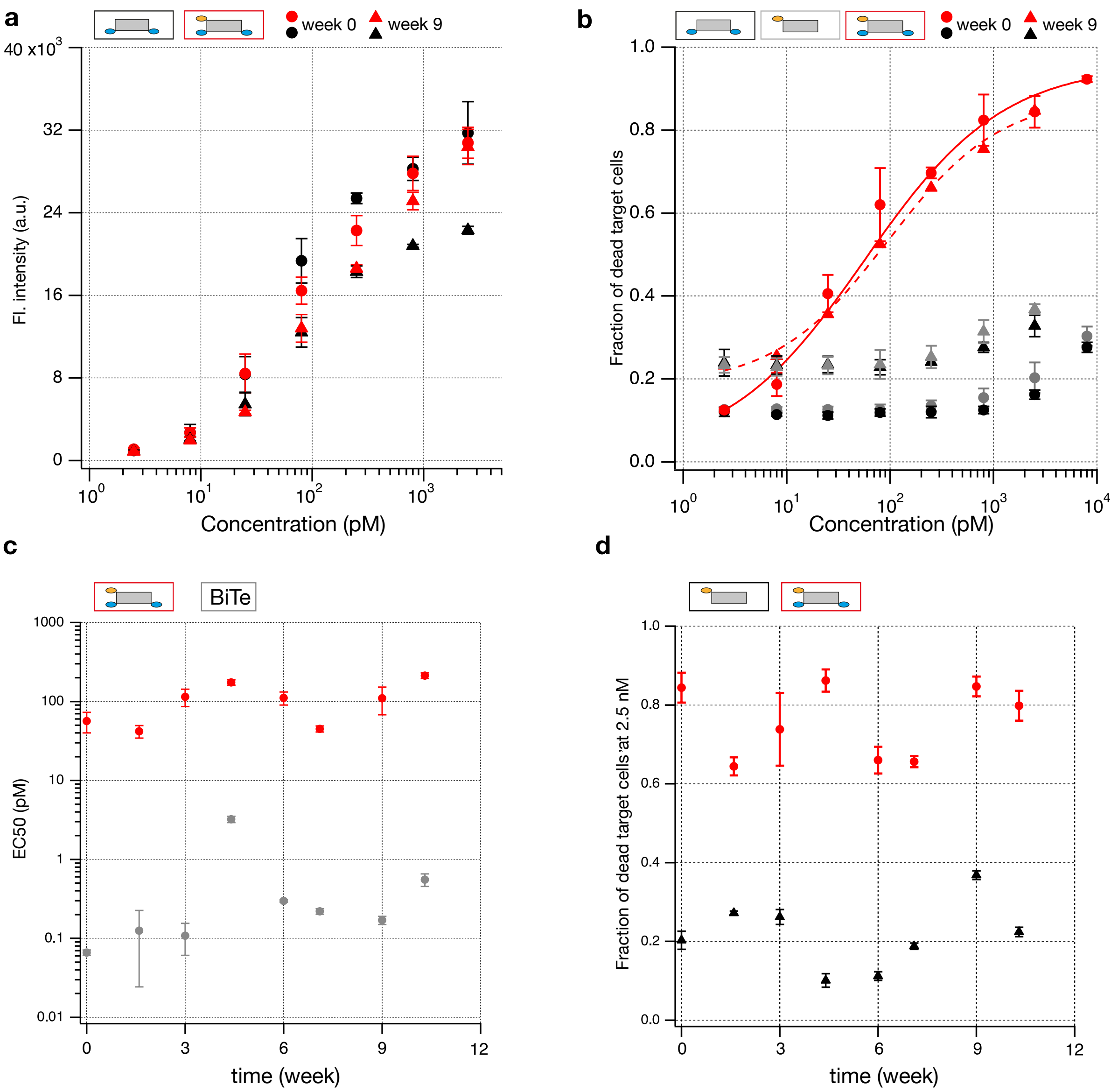

Fig. S16 | Long-term stability of programmable T-cell engagers at 4 °C a, binding behavior of different samples (2xCD19 (black) and 1CD3-2xCD19 (red) Fab fragments on a chassis, respectively) as a function of the concentration. Comparison between a sample prepared at day 0 (dots) and after 9 weeks at 4°C (triangles). **b,** samples as in a, fraction of dead target cells as a function of the concentration. Red lines (solid or dashed) Hill fits to the data for day 0 and after 9 weeks, respectively. **c,** EC50 values as a function of time for the PTE (red) and the Blinatumomab biosimilar (grey). **d,** Fraction of dead target cells (at 2.5 nM) for the PTE-2x19-3 (red) and an PTE-3 control sample (black) as a function of time. For a-d:Error bars are standard deviations to technical triplicates.

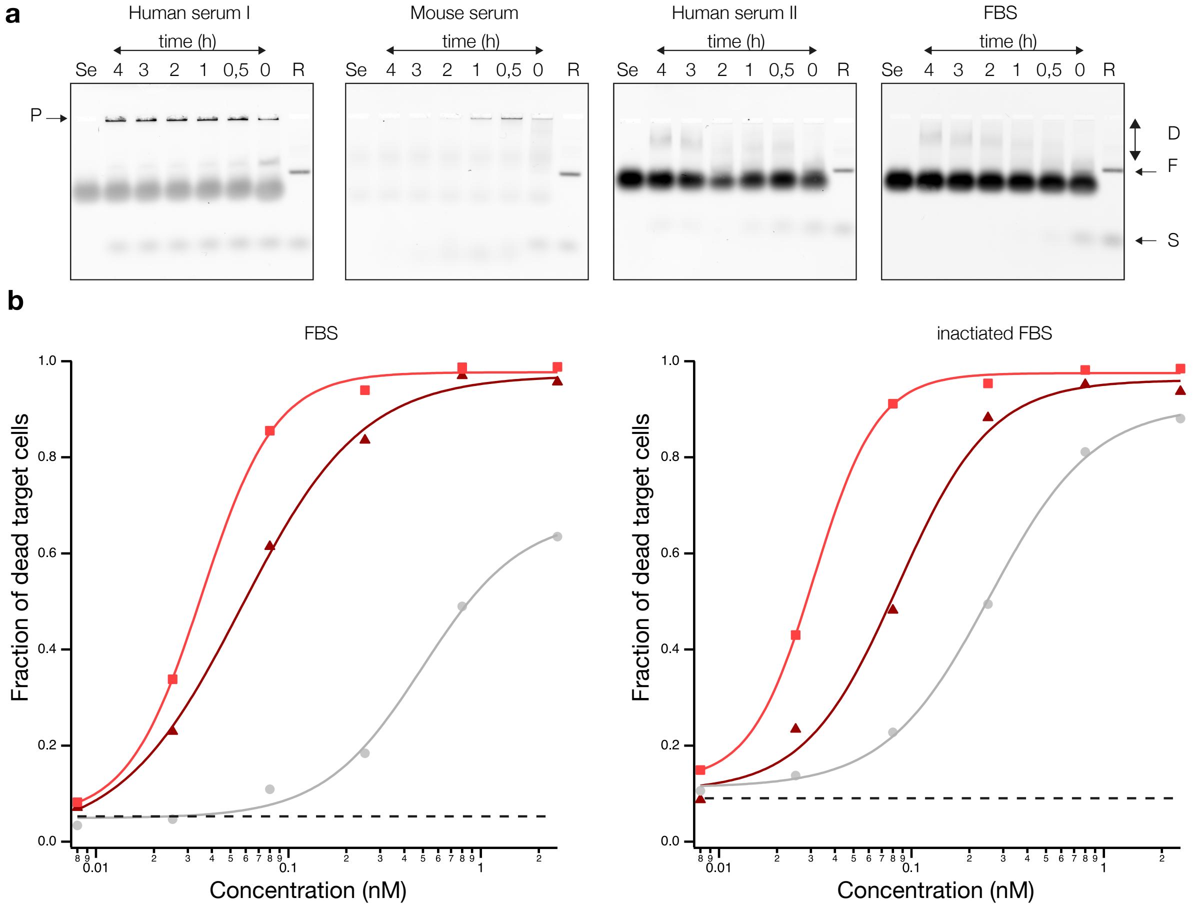

Fig. S17 | Serum stability of PTEs a, Incubation of unmodified and unprotected chassis in different sera. Laser scanned image of samples - Se (serum alone), R (DNA origami incubated for 4 h in PBS) and samples incubated for up to 4 h in the corresponding sera - electrophoretically separated on an agarose gel. Here, P marks the pocket of the gel, F the undamaged structure, S free DNA staples, and D shows the degraded sample spreading as smear after the pocket. Different brightnesses of the bands vary from serum to serum and correlate with their respective autofluorescence. **b,** Proportion of dead target cells as a function of the concentration of the applied molecule. Different coatings (red squares, dark red triangles) were tested for stabilization in FBS or inactive FBS (4 h preincubation) compared to a non-stabilized structure (grey circles). Solid line, Hill fits to the respective data, dashed line target cells incubated with PBMC at an E:T ratio of 5:1.

a

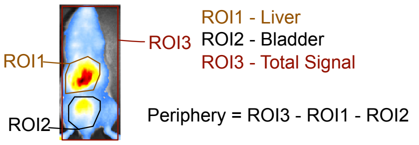

b

Gating Strategy - Flow Cytometry

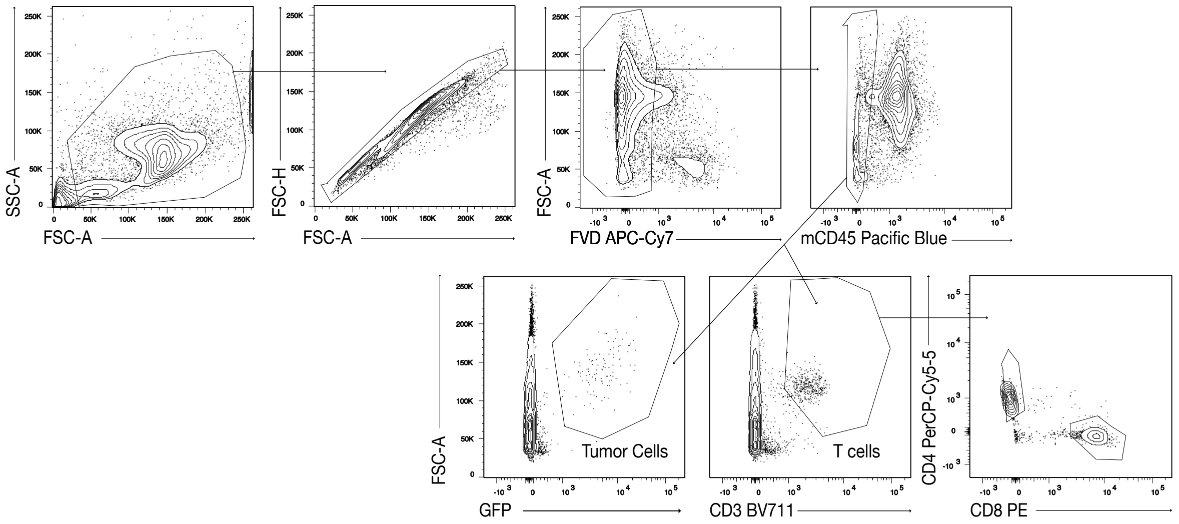

Fig. S18 | Data evaluation strategy a, Analysis of biodistribution data in animals. Region of interest (ROI) is marked for the bladder, liver and the whole animal, respectively. Intensity in the ROI is integrated of all pixels per region. **b,** Workflow for the gating strategy for flow cytometry data.

Supplementary Figure 19

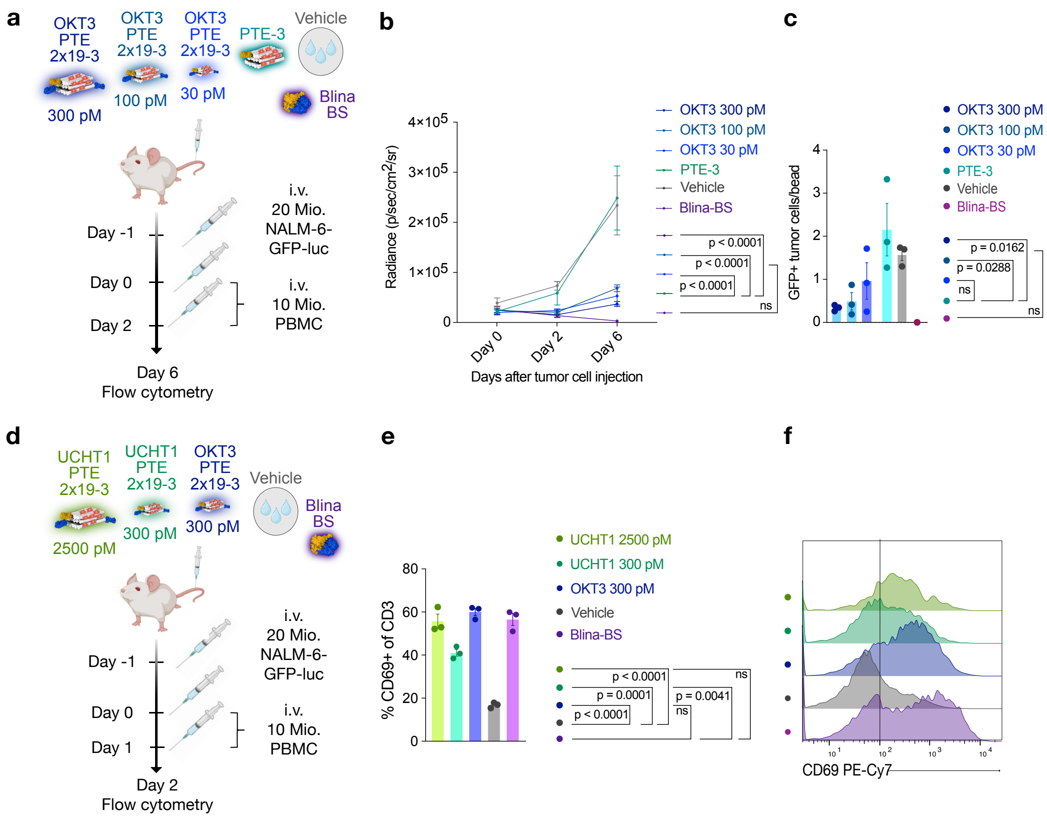

Fig. S19 | Determination of optimal *in vivo* dosage a, treatment schedule of PTE dose titration. Color scheme: blue: PTE-2xCD19-3 300 pM, 100 pM, 30 pM, respectively; teal: PTE-3; vehicle: grey; violet: Blina-BS. NALM-6-GFP-Luc, PBMC and samples were administered intravenously (i.v.) at the indicated time points and numbers in four- to eight-week-old, female NSG mice. N = 3 mice per group. **b**, Quantification of bioluminescence measurements. **c**, flow cytometric analysis of the tumor burden in the bone marrow after 6 days. Tumor cells were identified by gating for GFP+ cells. **b, c,** Data are mean ± s.e.m. from n = 3 mice. **d**, treatment schedule of comparisons between different CD3-binders. Color scheme: green: UCHT1-based 2x19-3 PTE (2500 pM, 300 pM; blue: OKT3-based PTE-2xCD19-3 (300 pM); vehicle: grey; violet: Blina-BS. NALM-6-GFP-Luc, PBMC and samples were administered intravenously (i.v.) at the indicated time points and numbers. N = 3 mice per group. **e**, Measurement of CD69 fluorescence on CD3+ T cells in the bone marrow of treated mice. **e,** Data are mean ± s.e.m. from n = 3 mice. **f**, representative flow cytometric images.

For all panels, statistical significance was calculated using ordinary one-way or two-way ANOVA with Tukey's multiple comparison correction.

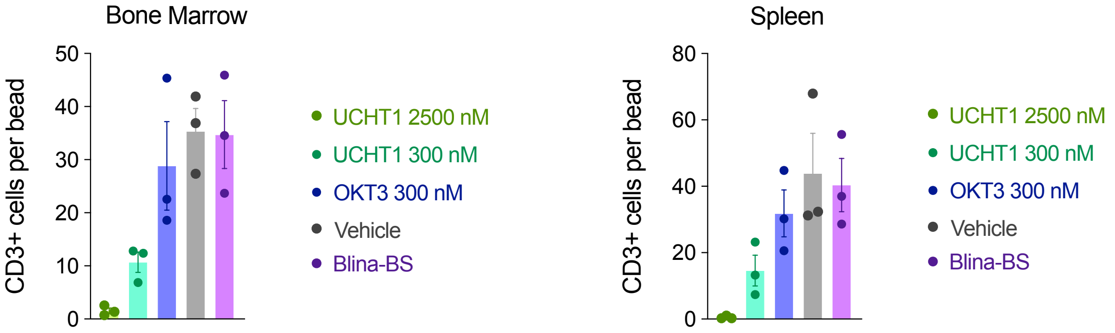

Fig. S20 | T-cell survival: fraction of CD3+ T cells after short term *in-vivo* treatments of four- to eight-week-old, female NSG mice with different PTEs, Blinatumomab, Vehicle in the bone marrow (left) and spleen (right). Different clones of anti CD3 F(ab)s were used for the PTE 2x19-3, namely UCHT1(grenn and cyan) and OKT3 (blue) at different concentrations, as indicated in the legend. n = 3 mice per group. Shown is mean ± s.e.m.

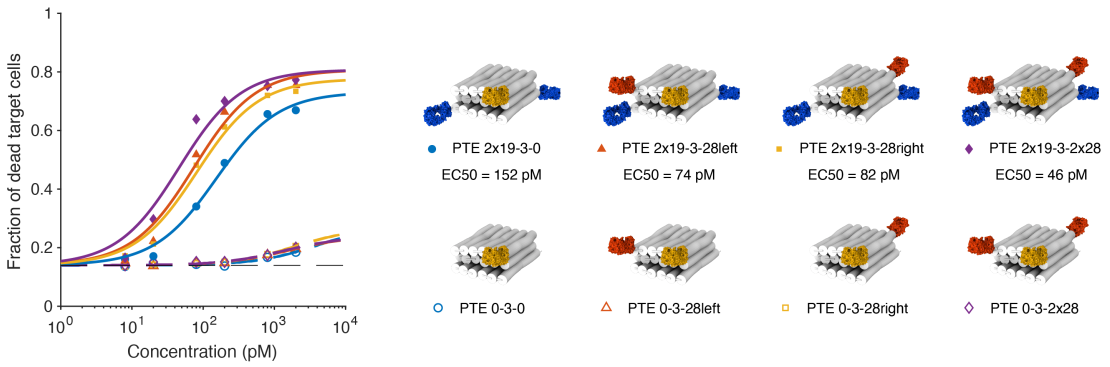

Fig. S21 | CD28-CD3 costimulation in cytotoxic T cell assays with F(ab) based chassis. Fraction of dead NALM6 target cells from cytotoxic T cell mediated target-cell lysis assay after 24 h as a function of the PTE concentration in the assay. Effector (Peripheral Blood Mononuclear cells, PBMC) and target cell ratio was chosen to 5:1. Lysis efficacies for PTE variants without (dashed lines) and with two (solid lines) aCD19 F(ab) fragments. All variants carried an aCD3 F(ab) fragment and various configurations fo aCD28 F(ab) fragments. Data was fitted with Hill-equiation to extract the potency (EC50). Black dashed lines indicates fraction of dead NALM6 cells where no construct has been added.

Supplementary References

1. Sandhöfer N, Metzeler K, Rothenberg M, Herold T, Tiedt S, Groiss V*, et al.* Dual PI3K/mTOR inhibition shows antileukemic activity in MLL-rearranged acute myeloid leukemia. *Leukemia* 2015, **29**(4)**:** 828-838.

2. Karches CH, Benmebarek M-R, Schmidbauer ML, Kurzay M, Klaus R, Geiger M*, et al.* Bispecific Antibodies Enable Synthetic Agonistic Receptor-Transduced T Cells for Tumor ImmunotherapyBispecific Antibodies Enable Transduced T Cells. *Clinical Cancer Research* 2019, **25**(19)**:** 5890-5900.

3. Engelhardt FA, Praetorius F, Wachauf CH, Brüggenthies G, Kohler F, Kick B*, et al.* Custom-size, functional, and durable DNA origami with design-specific scaffolds. *ACS nano* 2019, **13**(5)**:** 5015-5027.

4. Stahl E, Martin TG, Praetorius F, Dietz H. Facile and scalable preparation of pure and dense DNA origami solutions. *Angewandte Chemie* 2014, **53**(47)**:** 12735-12740.

5. Wagenbauer KF, Engelhardt FAS, Stahl EK, Hechtl VK, Stommer P, Seebacher F*, et al.* How we make DNA origami. *Chembiochem : a European journal of chemical biology* 2017.

6. Ponnuswamy N, Bastings MMC, Nathwani B, Ryu JH, Chou LYT, Vinther M*, et al.* Oligolysine-based coating protects DNA nanostructures from low-salt denaturation and nuclease degradation. *Nature Communications* 2017, **8:** 15654.

7. Humphreys DP, Heywood SP, Henry A, Ait-Lhadj L, Antoniw P, Palframan R*, et al.* Alternative antibody Fab′ fragment PEGylation strategies: combination of strong reducing agents, disruption of the interchain disulphide bond and disulphide engineering. *Protein Engineering, Design and Selection* 2007, **20**(5)**:** 227-234.

8. Stecha P, Garvin D, Hartnett J, Fan F, Cong M, Cheng Z-jJ. Improved T Cell Activation Bioassay for Development of Bispecific Antibodies and Engineered T Cell Immunotherapies. Madison (WI): Promega; 2016.

9. Liu H, Johnston AP. A programmable sensor to probe the internalization of proteins and nanoparticles in live cells. *Angewandte Chemie* 2013, **125**(22)**:** 5856-5860.

10. Malyala P, Singh M. Endotoxin limits in formulations for preclinical research. *Journal of pharmaceutical sciences* 2008, **97**(6)**:** 2041-2044.

11. McDermott SP, Eppert K, Lechman ER, Doedens M, Dick JE. Comparison of human cord blood engraftment between immunocompromised mouse strains. *Blood* 2010, **116**(2)**:** 193-200.

12. Shultz LD, Lyons BL, Burzenski LM, Gott B, Chen X, Chaleff S*, et al.* Human lymphoid and myeloid cell development in NOD/LtSz-scid IL2R gamma null mice engrafted with mobilized human hemopoietic stem cells. *J Immunol* 2005, **174**(10)**:** 6477-6489.

13. Lepus CM, Gibson TF, Gerber SA, Kawikova I, Szczepanik M, Hossain J*, et al.* Comparison of human fetal liver, umbilical cord blood, and adult blood hematopoietic stem cell engraftment in NOD-scid/gammac-/-, Balb/c-Rag1-/-gammac-/-, and C.B-17-scid/bg immunodeficient mice. *Hum Immunol* 2009, **70**(10)**:** 790-802.

14. Benmebarek M-R, Cadilha BL, Herrmann M, Lesch S, Schmitt S, Stoiber S*, et al.* A modular and controllable T cell therapy platform for acute myeloid leukemia. *Leukemia* 2021, **35**(8)**:** 2243-2257.

15. Lesch S, Blumenberg V, Stoiber S, Gottschlich A, Ogonek J, Cadilha BL*, et al.* T cells armed with CXC chemokine receptor type 6 enhance adoptive cell therapy for pancreatic tumours. *Nature biomedical engineering* 2021, **5**(11)**:** 1246-1260.

16. Gerling T, Wagenbauer KF, Neuner AM, Dietz H. Dynamic DNA devices and assemblies formed by shape-complementary, non–base pairing 3D components. *Science* 2015, **347**(6229)**:** 1446.

17. Douglas SM, Marblestone AH, Teerapittayanon S, Vazquez A, Church GM, Shih WM. Rapid prototyping of 3D DNA-origami shapes with caDNAno. *Nucleic acids research* 2009, **37**(15)**:** 5001-5006.

18. Bertosin E, Stömmer P, Feigl E, Wenig M, Honemann MN, Dietz H. Cryo-electron microscopy and mass analysis of oligolysine-coated DNA nanostructures. *ACS nano* 2021, **15**(6)**:** 9391-9403.

Supplementary Source Data

Fig. S1 e: Unmodified gels

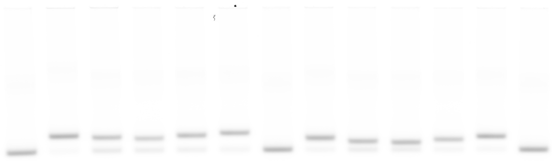

Fig. S1 f: Unmodified TEM-images

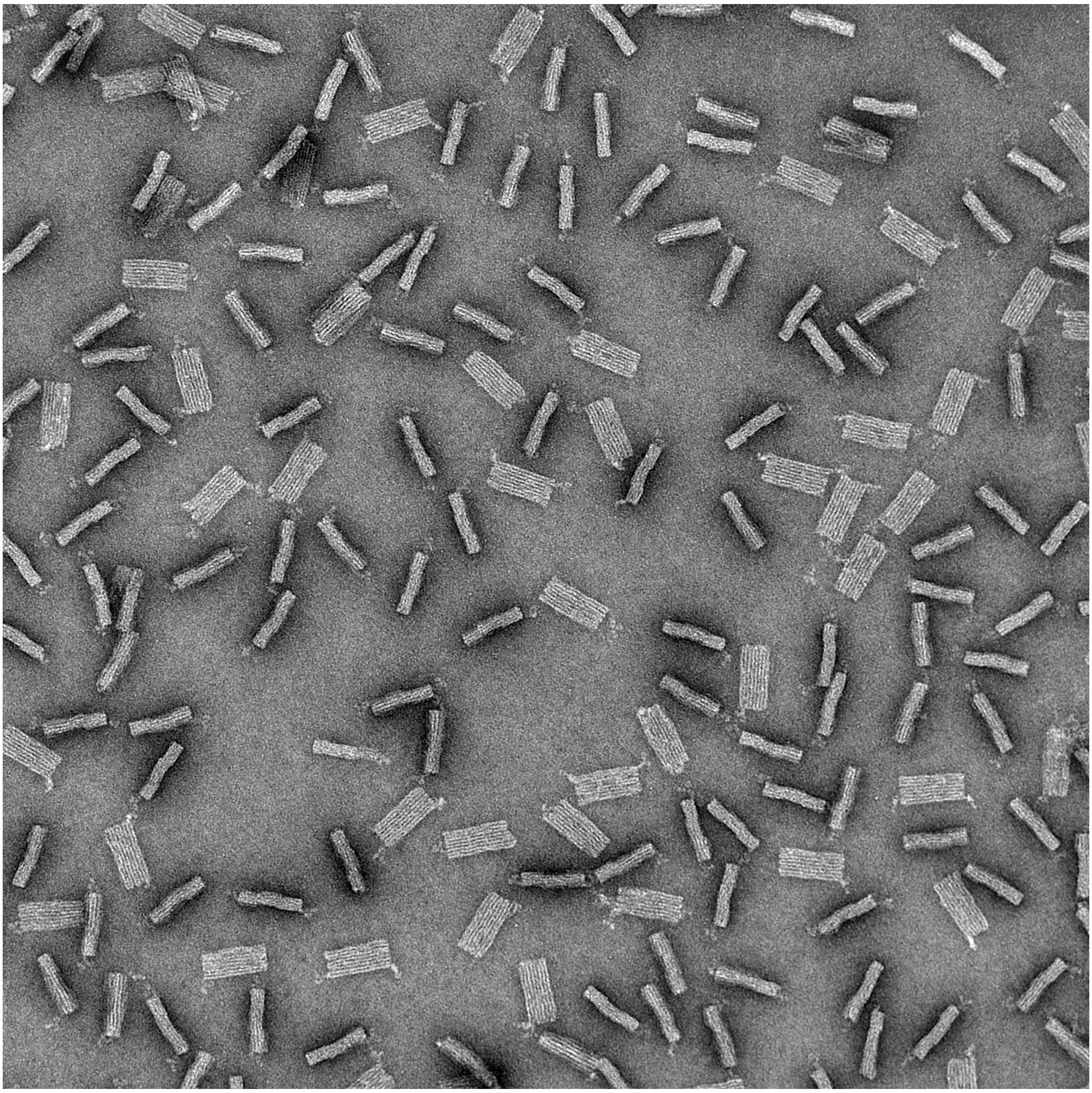

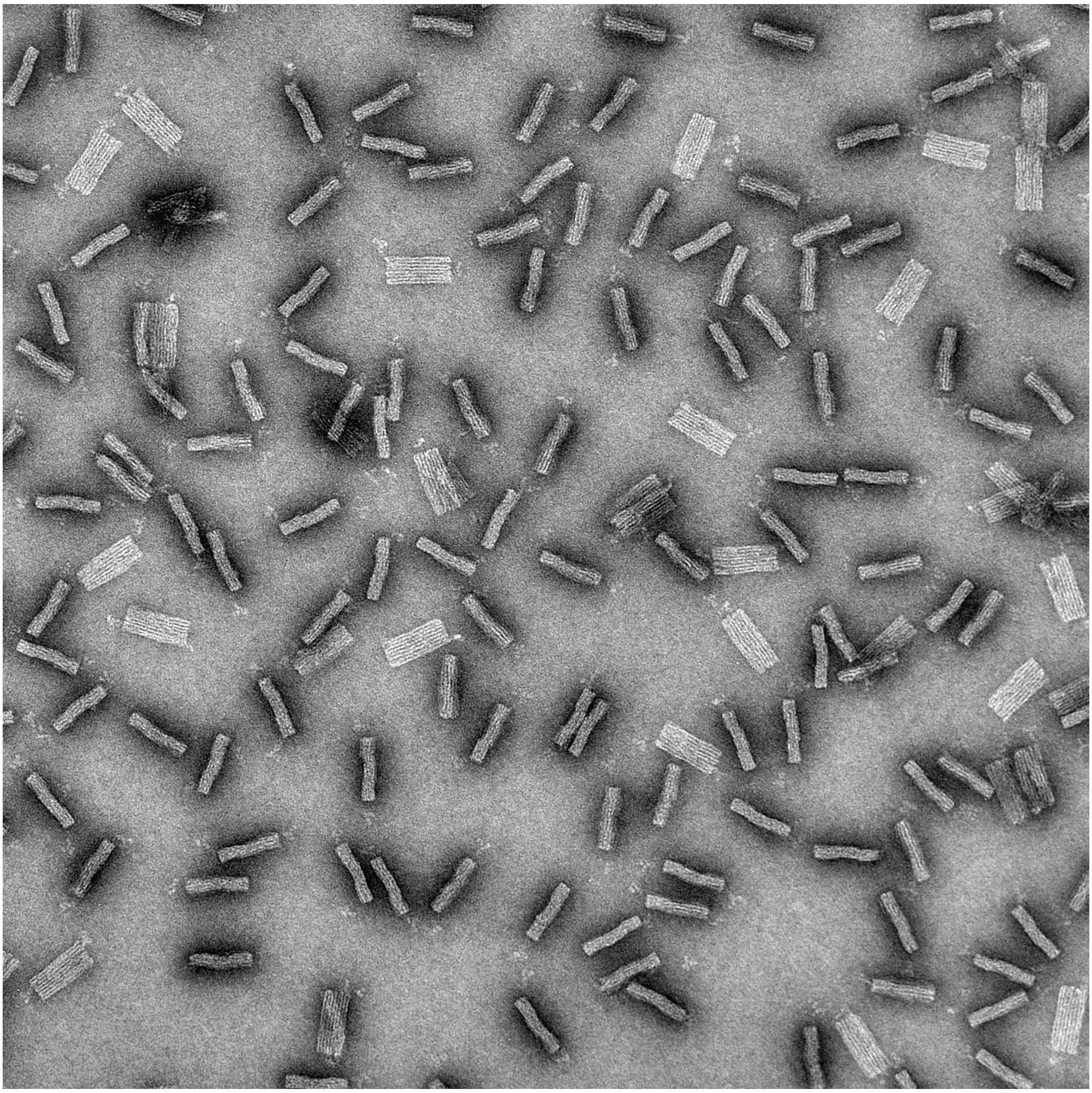

Fig. S4 a: Unmodified gel

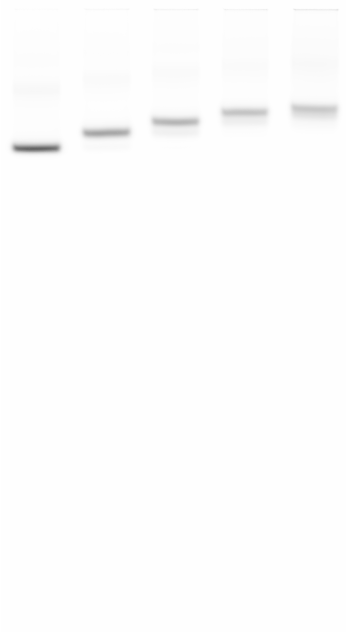

Fig. S10: Unmodified gel

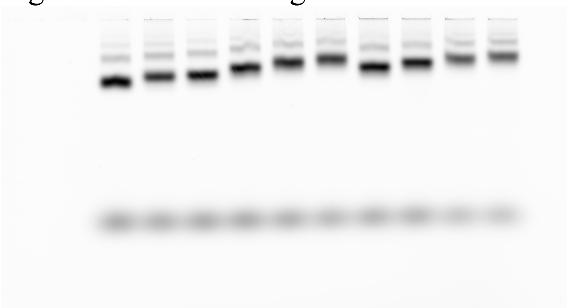

Fig. S17: Unmodified gels

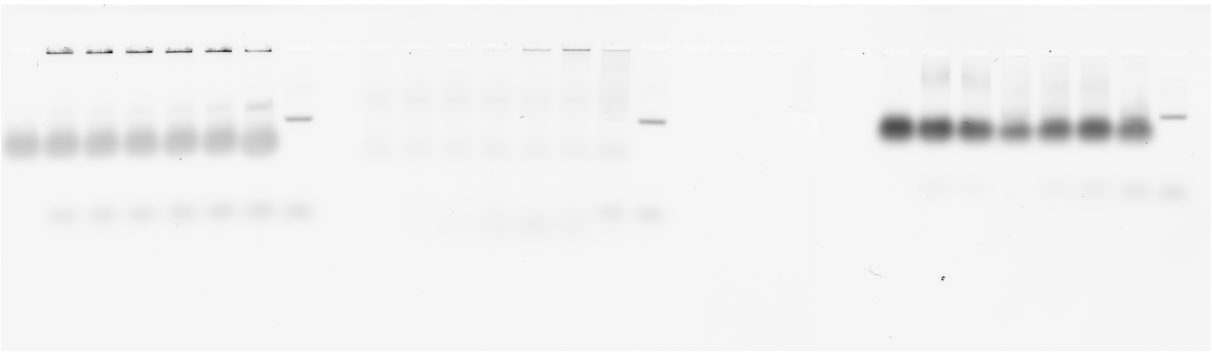

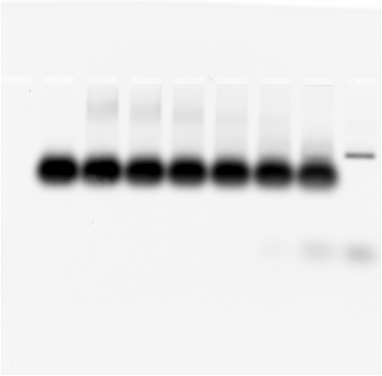

Supplement: Supplementary file 1 — Supplementary Notes 1–3, Figs. 1–21, Table 1, Methods and references. [file 41565_2023_1471_MOESM1_ESM.pdf]
