# Supplementary material for: Programmable multispecific DNA-origami-based T-cell engagers
Source: Nat Nanotechnol. 2023 Aug 17;18(11):1319–26. doi: 10.1038/s41565-023-01471-7 (PMC10656288; doi:10.1038/s41565-023-01471-7)
Supplement: Supplementary file 2 — Reporting Summary [file 41565_2023_1471_MOESM2_ESM.pdf]

nature portfolio

Corresponding author(s): *Double-anonymous peer review submissions: write DAPR and your manuscript number here instead of author names.*

Last updated by author(s): Jun 9, 2023

Reporting Summary

Nature Portfolio wishes to improve the reproducibility of the work that we publish. This form provides structure for consistency and transparency in reporting. For further information on Nature Portfolio policies, see our Editorial Policies and the Editorial Policy Checklist.

Statistics

For all statistical analyses, confirm that the following items are present in the figure legend, table legend, main text, or Methods section.

n/a	Confirmed	
☐	☒	The exact sample size (*n*) for each experimental group/condition, given as a discrete number and unit of measurement
☐	☒	A statement on whether measurements were taken from distinct samples or whether the same sample was measured repeatedly
☐	☒	The statistical test(s) used AND whether they are one- or two-sided<br>*Only common tests should be described solely by name; describe more complex techniques in the Methods section.*
☒	☐	A description of all covariates tested
☒	☐	A description of any assumptions or corrections, such as tests of normality and adjustment for multiple comparisons
☐	☒	A full description of the statistical parameters including central tendency (e.g. means) or other basic estimates (e.g. regression coefficient) AND variation (e.g. standard deviation) or associated estimates of uncertainty (e.g. confidence intervals)
☐	☒	For null hypothesis testing, the test statistic (e.g. *F*, *t*, *r*) with confidence intervals, effect sizes, degrees of freedom and *P* value noted<br>*Give P values as exact values whenever suitable.*
☒	☐	For Bayesian analysis, information on the choice of priors and Markov chain Monte Carlo settings
☒	☐	For hierarchical and complex designs, identification of the appropriate level for tests and full reporting of outcomes
☒	☐	Estimates of effect sizes (e.g. Cohen's *d*, Pearson's *r*), indicating how they were calculated

Our web collection on statistics for biologists contains articles on many of the points above.

Software and code

Policy information about availability of computer code

Data collection	Data were collected on different instruments. List of instruments is included in the methods section in the S.I.<br>Nanodrop 8000 Thermo Fisher, Attune Nxt Thermo Fisher, Typhoon GE, Profire Dynamic Biosensors, Platereader Clariostar BMG Labtech, Charles River NexGen PTS, CX7 Thermo Fisher, BD LSR Fortessa II, a Beckman Coulter CytoFLEX LX, Living Image 4.4 (PerkinElmer, USA)
Data analysis	Data analysis is described in detail in the S.I. Data were analyzed with the following software<br>Matlab 2021b, Igor Pro 7.06, Office 365, Fiji 2.1.0, FlowJo V10.3 to V10.8.1 software, GraphPad Prism V.9.4.0 (San Diego, CA, USA).

For manuscripts utilizing custom algorithms or software that are central to the research but not yet described in published literature, software must be made available to editors and reviewers. We strongly encourage code deposition in a community repository (e.g. GitHub). See the Nature Portfolio guidelines for submitting code & software for further information.

Data

Policy information about availability of data

All manuscripts must include a data availability statement. This statement should provide the following information, where applicable:

- Accession codes, unique identifiers, or web links for publicly available datasets
- A description of any restrictions on data availability
- For clinical datasets or third party data, please ensure that the statement adheres to our policy

All data and source data that support the findings of this study are available within the paper and its Supplementary Information, and from the corresponding author on request.

Research involving human participants, their data, or biological material

Policy information about studies with human participants or human data. See also policy information about sex, gender (identity/presentation), and sexual orientation and race, ethnicity and racism.

Reporting on sex and gender	N.A.
Reporting on race, ethnicity, or other socially relevant groupings	N.A.
Population characteristics	N.A.
Recruitment	N.A.
Ethics oversight	N.A.

Note that full information on the approval of the study protocol must also be provided in the manuscript.

Field-specific reporting

Please select the one below that is the best fit for your research. If you are not sure, read the appropriate sections before making your selection.

☒ Life sciences ☐ Behavioural & social sciences ☐ Ecological, evolutionary & environmental sciences

For a reference copy of the document with all sections, see nature.com/documents/nr-reporting-summary-flat.pdf

Life sciences study design

All studies must disclose on these points even when the disclosure is negative.

Sample size	For animal experiments sample size was determined using the software G*Power 3.1 with given alpha, power and effect size
Data exclusions	No data exclusion was done.
Replication	For TEM and gel electrophoretic analysis replicates were performed in different arangements. Images in the manuscript are examples of such experiments.<br>For in-vitro T-cell mediated lysis experiments in general biological triplicates were done if not otherwise stated and explainded. A20 cell line experiments were done once with Splenocytes due to ethical reasons.<br>T-cell activation experiments were done in technical dublicates.<br>For in vivo experiments, group size and number of independent repetitions are indicated. In vivo experiments were performed once (Fig. 4, Supplementary Figure 19, 20) or three-times (Fig. 3).
Randomization	Randomization was performed for all animal experiments. If tumor sizes showed strong variations, mice were stratified to ensure similiar tumor burden across all treatment groups.
Blinding	All animal experiments were performed blinded.

Reporting for specific materials, systems and methods

We require information from authors about some types of materials, experimental systems and methods used in many studies. Here, indicate whether each material, system or method listed is relevant to your study. If you are not sure if a list item applies to your research, read the appropriate section before selecting a response.

Materials & experimental systems

n/a	Involved in the study
☐	☒ Antibodies
☐	☒ Eukaryotic cell lines
☒	☐ Palaeontology and archaeology
☐	☒ Animals and other organisms
☒	☐ Clinical data
☒	☐ Dual use research of concern
☒	☐ Plants

Methods

n/a	Involved in the study
☒	☐ ChIP-seq
☐	☒ Flow cytometry
☒	☐ MRI-based neuroimaging

Antibodies

Antibodies used

Antibody Clon Vendor ID
CD3 UTCH1 ThermoFisher 16-0038-85
CD3 OKT3 Biozol BE0001-2
CD3 OKT3 Icosagen Custom - scFv
CD19 HIB19 ThermoFisher 16-0199-85
CD123 6H6 ThermoFisher 14-1239-37
CD22 eBio4KB128 (4KB128) ThermoFisher 14-0229-82
CD33 WM-53 ThermoFisher 14-0338-37
CD28 CD28.2 ThermoFisher 16-0289-85
CD137 5G11 ThermoFisher 14-9056-82
muCD19 1D3 Biozol BE0150
muCD3 F(ab)2 145-2C11 Biozol 711721J1
EpCAM VU-1D9 ThermoFisher MA5-12153
PSMA GCP-05 ThermoFisher MA1-10335
CD8a Monoclonal Antibody (RPA-T8) ThermoFisher
CD69 Monoclonal Antibody (FN50), PerCP-eFluor™ 710 ThermoFisher
CD4 Monoclonal Antibody (RPA-T4), PE ThermoFisher
CD25 Monoclonal Antibody (PC61.5), PE-eFluor™ 610 ThermoFisher

Flow cytometry:
TrueStain FcX™ (BioLegend, USA, catalog #422302)
fixable viability dye eFluor™ 780 (eBioscience, USA, catalog #65-0865-18)
anti-murine CD45 - PacBlue (Biolegend, clone: 30-F11, catalog #103126)
anti-human CD69 PE-Cy7 (human – clone: FN50, catalog #310912, Biolegend, USA)

anti-CD3 (Biolegend, BV711 - clone: UCHT1, catalog 300464)
anti-CD4 (Biolegend, human, PerCP-Cy5.5 - clone: OKT4, catalog #317428)
anti-CD8 (Biolegend, human, PE – clone HIT8a, catalog #300908)

Validation

CD3 UTCH1 ThermoFisher 16-0038-85 https://www.thermofisher.com/antibody/product/CD3-Antibody-clone-UCHT1-Monoclonal/16-0038-85
CD3 OKT3 Biozol BE0001-2 https://www.biozol.de/InVivoMab-anti-human-CD3-Clone-OKT-3-Mouse-Monoclonal/BXC-BE0001-2-1MG
CD3 OKT3 Icosagen Custom - scFv
CD19 HIB19 ThermoFisher 16-0199-85 https://www.thermofisher.com/antibody/product/CD19-Antibody-clone-HIB19-Monoclonal/16-0199-85
CD123 6H6 ThermoFisher 14-1239-37 https://www.thermofisher.com/antibody/product/CD123-Antibody-clone-6H6-Monoclonal/14-1239-37
CD22 eBio4KB128 (4KB128) ThermoFisher 14-0229-82 https://www.thermofisher.com/antibody/product/CD22-Antibody-clone-eBio4KB128-4KB128-Monoclonal/14-0229-82
CD33 WM-53 ThermoFisher 14-0338-37 https://www.thermofisher.com/antibody/product/CD33-Antibody-clone-WM-53-WM53-Monoclonal/14-0338-37
CD28 CD28.2 ThermoFisher 16-0289-85 https://www.thermofisher.com/antibody/product/CD28-Antibody-clone-CD28-2-Monoclonal/16-0289-85
CD137 5G11 ThermoFisher 14-9056-82 https://www.thermofisher.com/antibody/product/CD137-Ligand-4-1BB-Ligand-Antibody-clone-5G11-Monoclonal/14-9056-82
mUCD19 1D3 Biozol BE0150 https://www.biozol.de/InVivoMab-anti-mouse-CD19-Clone-1D3-Rat-Monoclonal/BXC-BE0150-5MG
muCD3 F(ab)2 145-2C11 Biozol 711721J1 https://www.biozol.de/CD3-antibody-145-2C11-Clone-145-2C11-Monoclonal/CVL-00114275
EpCAM VU-1D9 ThermoFisher MA5-12153 https://www.thermofisher.com/antibody/product/EpCAM-Antibody-clone-VU-1D9-Monoclonal/MA5-12153
PSMA GCP-05 ThermoFisher MA1-10335 https://www.thermofisher.com/antibody/product/PSMA-Antibody-clone-GCP-05-Monoclonal/MA1-10335
CD8a Monoclonal Antibody (RPA-T8) https://www.thermofisher.com/antibody/product/CD8a-Antibody-clone-RPA-T8-Monoclonal/17-0088-42
CD69 Monoclonal Antibody (FN50), PerCP-eFluor™ 710 https://www.thermofisher.com/antibody/product/CD69-Antibody-clone-FN50-Monoclonal/46-0699-42
CD4 Monoclonal Antibody (RPA-T4), PE https://www.thermofisher.com/antibody/product/CD4-Antibody-clone-RPA-T4-Monoclonal/

MA1-81104
CD25 Monoclonal Antibody (PC61.5), PE-eFluor™ 610 https://www.thermofisher.com/antibody/product/CD25-Antibody-clone-PC61-5-Monoclonal/61-0251-80

TrueStain FcX™ (https://www.biolegend.com/en-us/products/human-trustain-fcx-fc-receptor-blocking-solution-6462?GroupID=BLG2181)
fixable viability dye eFluor™ 780 (eBioscience, USA, catalog #65-0865-18)
anti-murine CD45 - PacBlue (https://www.biolegend.com/en-us/products/pacific-blue-anti-mouse-cd45-antibody-19250?GroupID=GROUP20)
anti-human CD69 PE-Cy7 (https://www.biolegend.com/en-us/products/pe-cyanine7-anti-human-cd69-antibody-1918)

anti-CD3 (ttps://www.biolegend.com/en-us/products/brilliant-violet-711-anti-human-cd3-antibody-12047)
anti-human CD4 PerCP-Cy5.5 (https://www.biolegend.com/ja-jp/products/percp-cyanine5-5-anti-human-cd4-antibody-5011)
anti-human CD8 PE (https://www.biolegend.com/en-ie/products/pe-anti-human-cd8a-antibody-762)

Eukaryotic cell lines

Policy information about cell lines and Sex and Gender in Research

Cell line source(s)

Jurkat https://www.dsmz.de/collection/catalogue/details/culture/ACC-282
NALM-6 https://www.dsmz.de/collection/catalogue/details/culture/ACC-128
MCF-7 https://www.dsmz.de/collection/catalogue/details/culture/ACC-115
Molm-13 https://www.dsmz.de/collection/catalogue/details/culture/ACC-554
LNCaP https://cls.shop/LNCaP/300265
NALM-6 (ATCC, USA)

Authentication

Cell line Authentification was done by vendor and via PCR by Eurofins Genomics.
STR DNA profiling of human cell lines (NALM-6).

Mycoplasma contamination

All cell lines were tested negative for mycoplasma contamination.

Commonly misidentified lines
(See ICLAC register)

No commonly misidentified lines were used.

Animals and other research organisms

Policy information about studies involving animals; ARRIVE guidelines recommended for reporting animal research, and Sex and Gender in Research

Laboratory animals

NSG (NOD.Cg-PrkdcSCIDIl2rgtm1Wjl/SzJ mice, Charles River, Janiver or breeding in the local animal facility "Zentrale Versuchstierhaltung Innenstadt", male or female, 4-12 weeks and >16g body weight at the start of the experiments).
Mice were held in facilities with a 12-hours dark/light cycle including a 30 min twilight phase of 30 minutes at noise levels below 50 dBA. Air velocity was held below 0.2 m/s. Air humidity in the facilities were between 45 – 60% and average temperature was held between 20 to 22 °C.

Wild animals

No wild animals were used in the study.

Reporting on sex

Findings are applicable to both male and female mice.

Field-collected samples

No field collected samples were used in the study.

Ethics oversight

Local regulatory agency (Regierung von Oberbayern)

Note that full information on the approval of the study protocol must also be provided in the manuscript.

Flow Cytometry

Plots

Confirm that:

☒ The axis labels state the marker and fluorochrome used (e.g. CD4-FITC).

☒ The axis scales are clearly visible. Include numbers along axes only for bottom left plot of group (a 'group' is an analysis of identical markers).

☒ All plots are contour plots with outliers or pseudocolor plots.

☒ A numerical value for number of cells or percentage (with statistics) is provided.

Methodology

Sample preparation

Cell surface binding experiments

Sample preparation

For flow cytometry experiments, the cells were grown to a cell density of 1.5-2 *10^6 cells ml-1 in T-75 cell culture flasks. The cells were centrifuged for 5 min at 160 rcf and washed with ice-cold PBS, twice. All flow cytometry experiments were executed at a cell density of 2*10^7 cells ml-1 in PBS or medium. The sample (Chassis or PTE) was added at a final concentration of 1 nM and incubated for the different time points. Before flow cytometry analysis, the cells were centrifuged for 5 min at 500 rcf and resuspended to a final cell concentration of 2*10^6 cell ml-1 in PBS. Flow cytometric analysis was performed on a Cytoflex (Beckman Coulter) or Attune Nxt (ThermoFisher), measuring the fluorescent intensity by excitation at 640 nm and a bandpass detection filter at 660/20nm. The single cells were gated based on the forward scatter versus the side scatter.

Cytotoxic T-cell killing assay of liquid tumor cells:
Assay: 2x10^5 CSFE stained target cells per ml were incubated with 1x10^6 PBMC/ml in the cell-culture medium at 37°C (5% CO2) and the programmable T-cell engagers in different concentrations or without an additional recruiter. Cell fluorescence and scattering intensity was determined using an Attune Nxt flow cytometry with a Cytkick Max autosampler (ThermoFisher).

Flow cytometry for in vivo experiments
Single-cell suspensions of harvested organs were stained with human anti-CD3 BV711 (clone: OKT3), anti-CD4 PerCP-Cy5.5 (clone: OKT4), anti-CD8 PE (clone: HIT8a), anti-CD19 BV786 (clone: HIB19) and anti-CD69 PE-Cy7 (clone: FN50) or mouse anti-CD45 pacific blue (clone: 30-F11) antibodies (Biolegend, USA). Fixable viability dye (eFluor™ 780, eBioscience, USA) was used to exclude dead cells.

TISSUE PREPARATION
- mechanical disintegration
- only for tumor tissue: collagense/DNase digestion (37°C, 30min)
- pass through a cell strainer to get single cell suspensions
- only for brain tissue: brain gradient centrifugation
- wash step with PBS/2%FCS
- FACS staining (4°C, 30min)
- wash step with PBS/2%FCS
- FACS analysis in PBS/2% FCS

CELL CULTURE EXPERIMENTS
- wash T cells with PBS
- FACS staining (4°C, 30min)
- wash with PBS
- FACS analysis

Instrument

BD FACS Aria II (BD bioscience, Germany) - for FACS sorting
FACS Canto II (BD bioscience, Germany)
FACS Fortessa (BD bioscience, Germany)
Beckmann Coulter CytoFLEX
Thermo Fisher Attune Nxt with autoloader.

Software

Flow cytometric data were analyzed using FlowJo V10.3 to V10.8.1 software, BD FACSDiva (BD bioscience, Germany), and with Matlab 2021b. Data was then processed with Matlab 2021b, Excel 365 or GraphPad Prism V.9.4.0.

Cell population abundance

Post-sort purity was >90%

Gating strategy

Binding :
FSC/SSC -> Singlet
-> Cy5+ signal -> Binding of PTE

Killing:
CSFE+ cells -> target cells
FSC/SSC -> Dead/alive cells

Activation
CSFE- cells -> PBMCs
FSC/SCC -> T-cells
CD8+ -> CD69+
CD4+ -> CD69+

Animal experiments:
FSC/SSC -> Singlet -> FVD -> murine CD45 negative
-> GFP+ = Nalm-6 GFP+ tumor cells -
-> Cy5+ : Binding of PTE
-> CD19+: Binding of PTE and Blina-BS

-> CD3+ -> T cells
-> CD4+ -> CD69+
-> CD8+ -> CD69+

☒ Tick this box to confirm that a figure exemplifying the gating strategy is provided in the Supplementary Information.
