# Supplementary material for: Programmable multispecific DNA-origami-based T-cell engagers
Source: Nat Nanotechnol. 2023 Aug 17;18(11):1319–26. doi: 10.1038/s41565-023-01471-7 (PMC10656288; doi:10.1038/s41565-023-01471-7)
Supplement: Supplementary file 5 — Statistical source data. [file 41565_2023_1471_MOESM5_ESM.pdf]

Source Data Figure 1 to 4

Source Data Figure 1a:

Single Particle library of class averages.

Without IgG

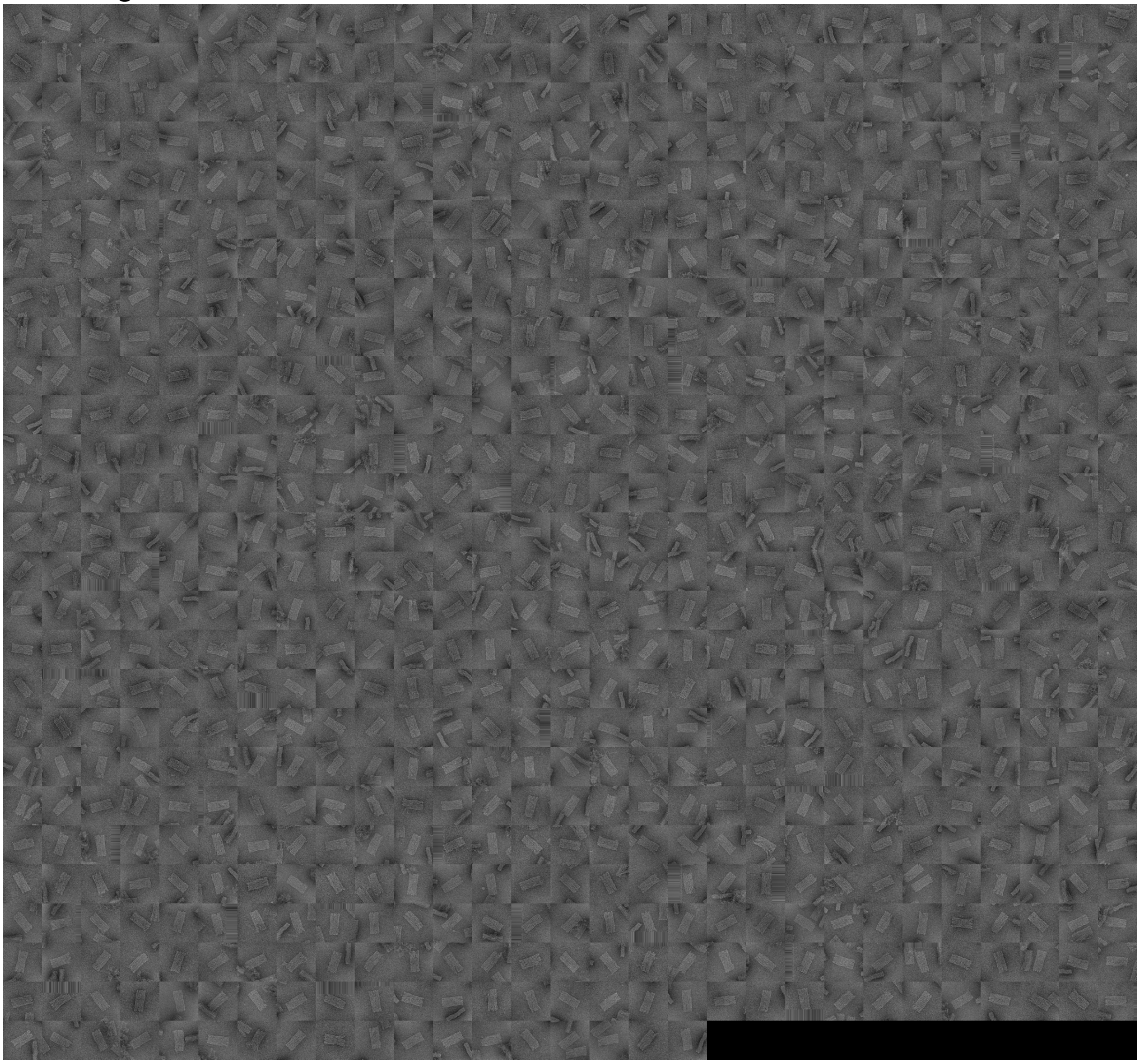

With 4 IgGs bound

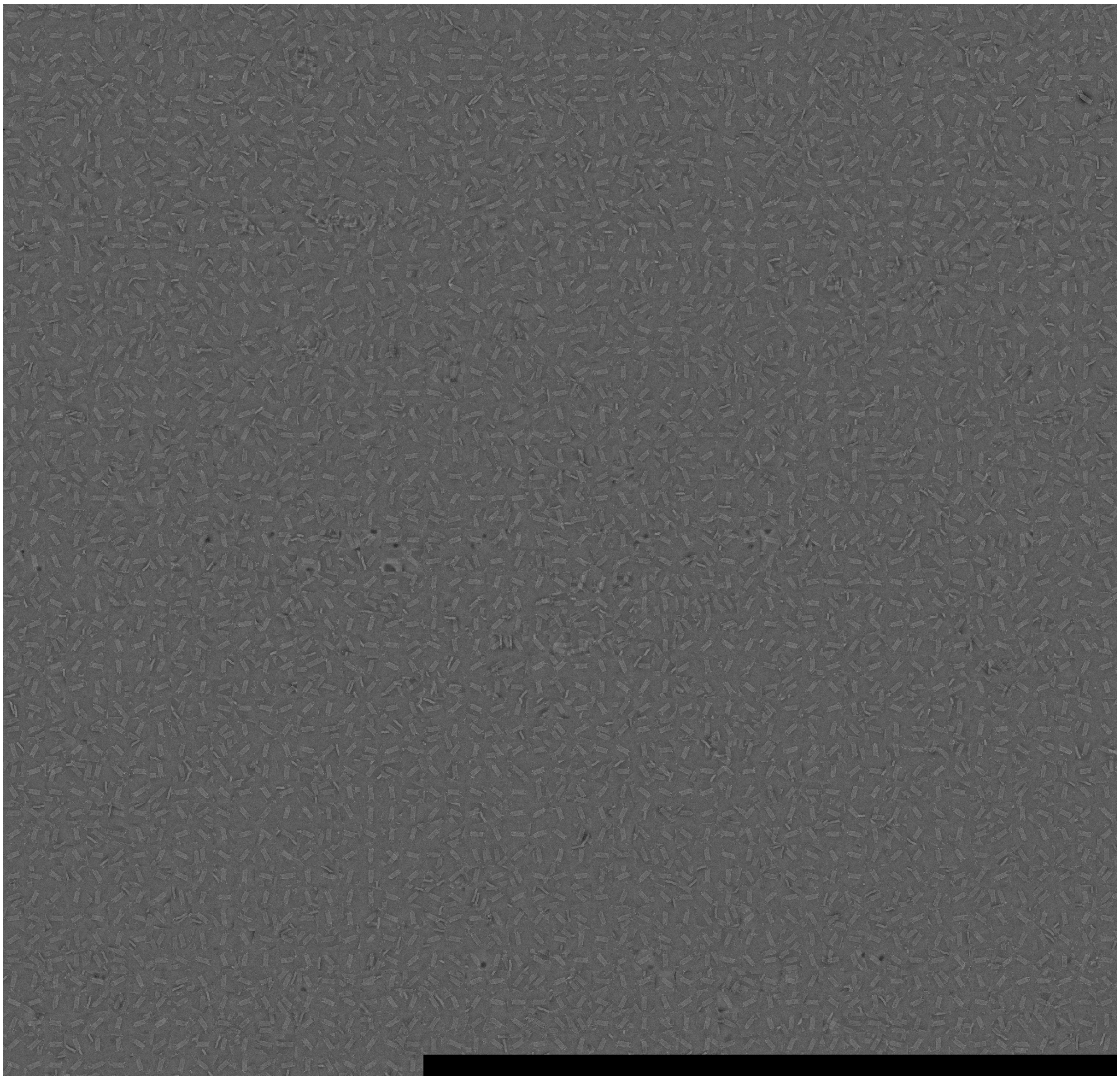

Source Data Figure 1b:
Gel data in the main figure is a merge of 7 individual gels with reference markers (scaffold band only) on each gel for alignment.

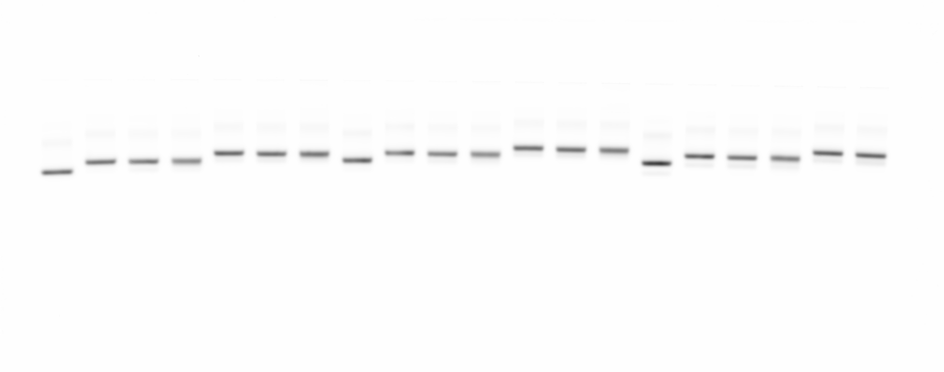

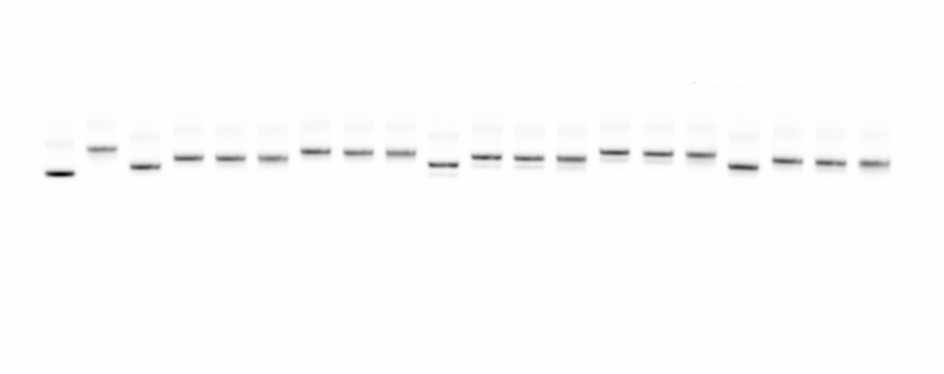

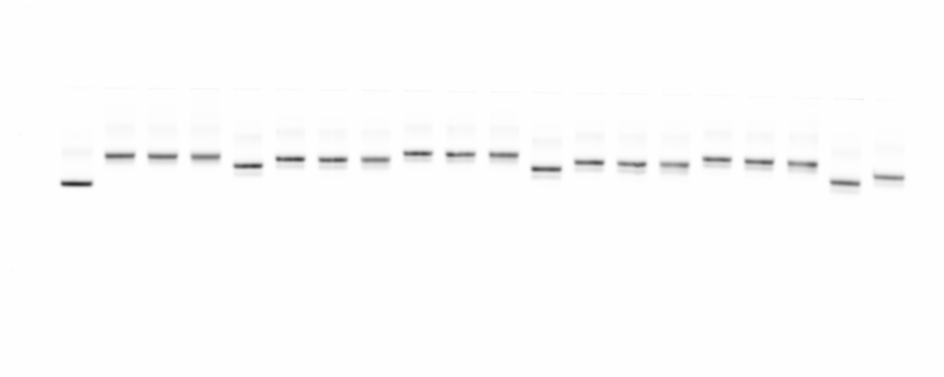

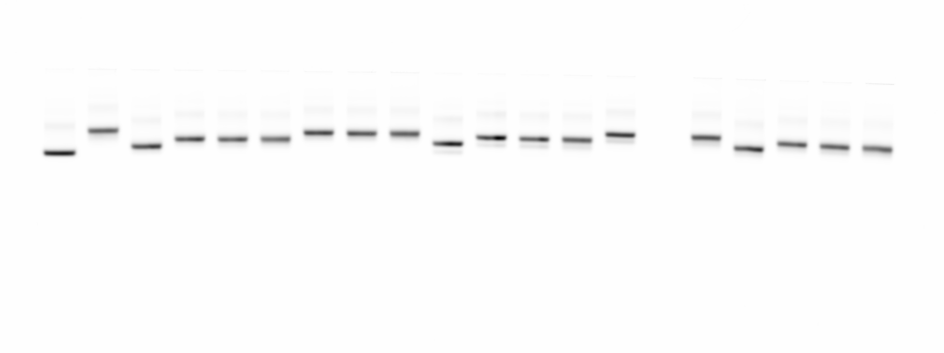

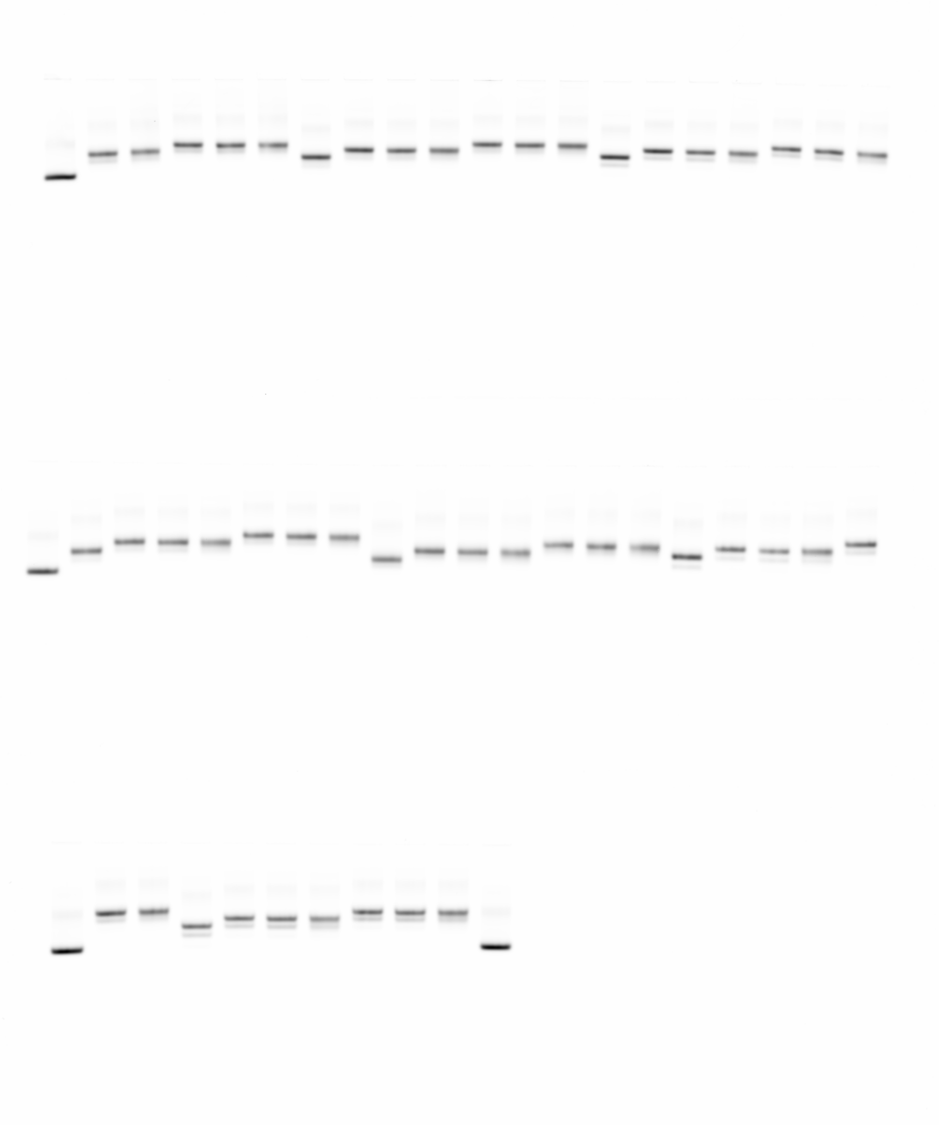

Source Data Figure 2b:
Unprocessed TEM images

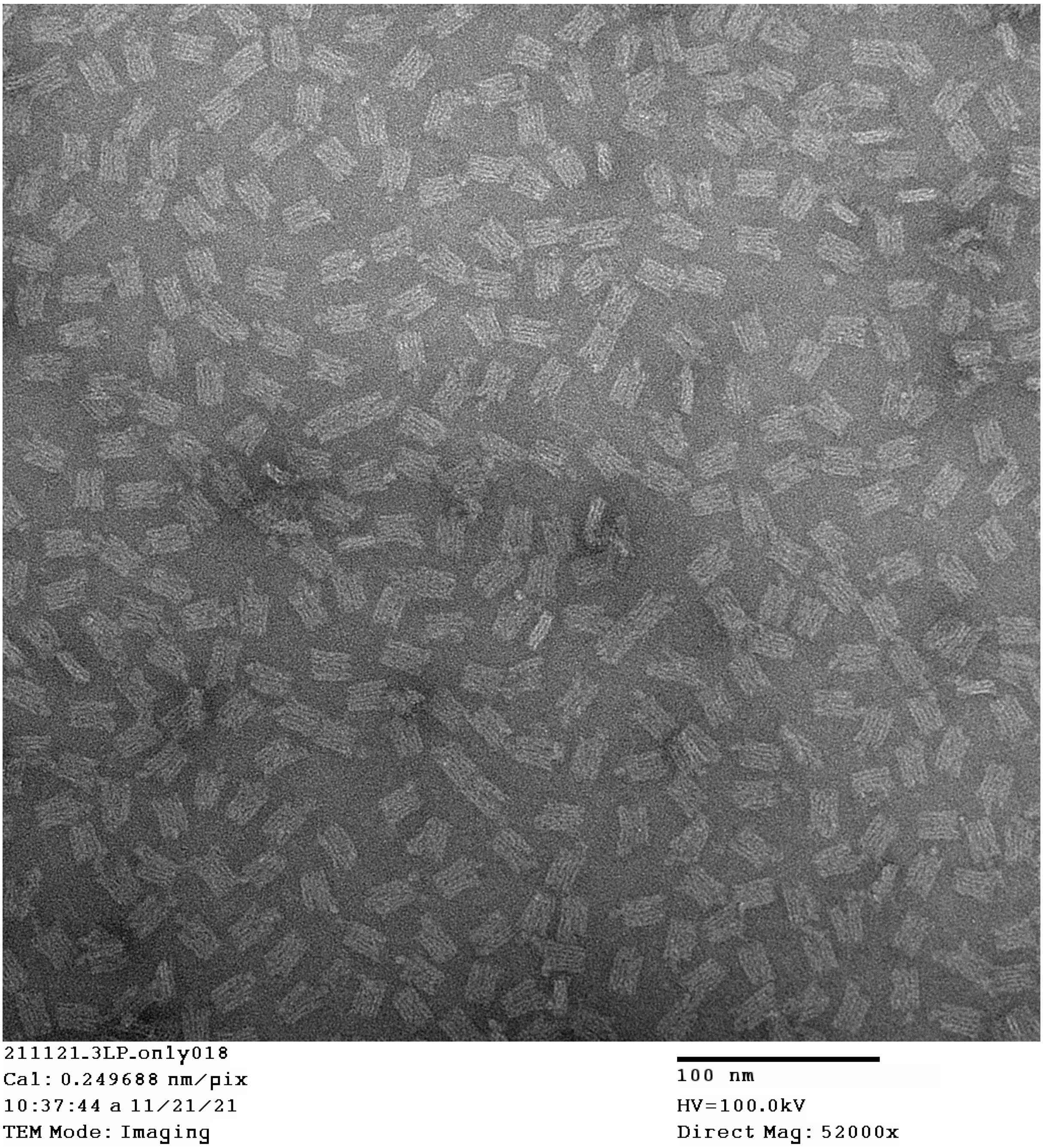

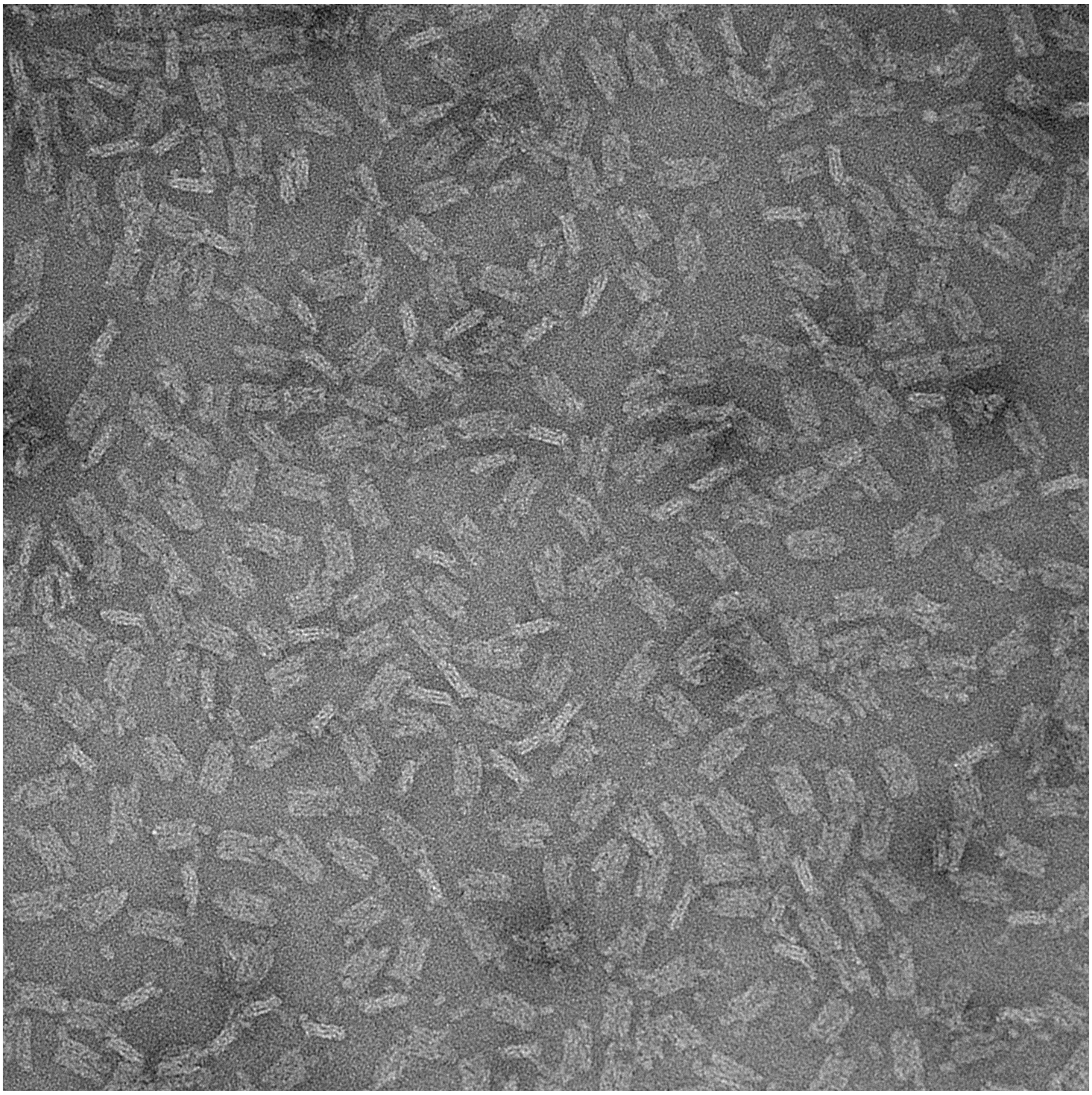

211121_3LP_1_2017
Cal: 0.249688 nm/pix
11:23:18 a 11/21/21
TEM Mode: Imaging

100 nm
Direct Mag: 52000x

Source Data Figure 2c:
Unprocessed gel image
